# Pericentrosomal Redistribution of the Endoplasmic Reticulum Ensures Organelle Symmetric Inheritance and Mitotic Progression

**DOI:** 10.1002/advs.76193

**Published:** 2026-06-22

**Authors:** Xiangyu Xu, Yalin Liu, Rongyi Wang, Wenwen Xu, Hao Shi, Ning Huang, Junlin Teng, Jin Meng, Pengli Zheng, Jianguo Chen

**Affiliations:** ^1^ Key Laboratory of Cell Proliferation and Differentiation of the Ministry of Education College of Life Sciences Peking University Beijing China; ^2^ School of Basic Medical Sciences Capital Medical University Beijing China; ^3^ Institute of Neuroscience Translational Medicine Institute Health Science Center Xi'an Jiaotong University Xi'an China; ^4^ Department of Physiology and Pathophysiology School of Basic Medical Sciences Health Science Center Xi'an Jiaotong University Xi'an China; ^5^ Chinese Institutes For Medical Research Beijing China; ^6^ Center for Life Sciences Academy for Advanced Interdisciplinary Studies Peking University Beijing China; ^7^ Center for Quantitative Biology Academy for Advanced Interdisciplinary Studies Peking University Beijing China

**Keywords:** endoplasmic reticulum, mitosis, organelle inheritance, Rab11 GTPase, Reticulon 4

## Abstract

Symmetric cell division entails the equal distribution of cellular components to daughter cells. However, the mechanisms governing organelle segregation remain elusive. The endoplasmic reticulum (ER), comprising perinuclear sheets and peripheral tubules in interphase, serves as a central hub for sensing cellular states and coordinating other organelle dynamics. Here, we show that upon mitotic entry, the ER undergoes reverse redistribution: tubular ER accumulates around the centrosomes, while sheet‐like ER relocates to the periphery. Mechanistically, the tubular ER protein Reticulon 4 (RTN4) is phosphorylated by cyclin‐dependent kinase 1 (CDK1) during early mitosis. Phosphorylation promotes the interaction between RTN4 and Rab11, leading to the dynein‐dependent enrichment of RTN4 around centrosomes and consequently driving the tubularization of the pericentrosomal ER. RTN4‐mediated mitotic ER reorganization ensures symmetric distribution and inheritance of the ER, further contributing to the symmetric segregation of other organelles and mitotic fidelity. Thus, our study uncovers the mechanism of ER symmetry remodeling during early mitosis and its roles in organelle inheritance and mitotic progression.

## Graphical abstract

Upon mitotic entry, RTN4 relocalizes to the pericentrosomal region, forming a more tubular ER network around centrosomes. CDK1‐mediated phosphorylation of RTN4 increases its interaction with Rab11 GTPase, facilitating dynein‐dependent transport of RTN4 to the pericentrosomal region. RTN4‐mediated ER mitotic reorganization promotes the symmetric distribution and equal inheritance of the ER and other organelles and is essential for proper mitotic progression.

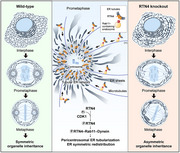

## 1 Introduction

Symmetric mitosis is characterized by the precise partitioning of genetic material and cytoplasmic components, including organelles, between daughter cells. Faithful organelle inheritance is crucial for cell fate determination [1, 2]. However, while the mechanisms of equal chromosome segregation have been extensively elucidated, those ensuring symmetric organelle inheritance remain poorly understood.

In interphase, the interconnected endoplasmic reticulum (ER) network coordinates interorganelle crosstalk [3]; it comprises structurally and functionally distinct compartments, including the peripheral ER tubules, the perinuclear dense sheet‐like ER, and the nuclear envelope [4, 5]. A variety of membrane proteins contribute to shaping the morphology of the peripheral ER. Reticulons (RTNs) and receptor expression‐enhancing proteins (REEPs) stabilize the membrane curvature of the tubular ER through oligomerization and their wedge‐shaped tandem hairpin reticulon homology domains (RHDs) [6]. Coiled‐coil‐containing membrane proteins, including cytoskeleton‐linking membrane protein 63 kDa (CLIMP63), kinectin 1 (KTN1), and P180, are enriched in ER sheets and maintain their structure [7]. The distribution of the interphase ER is asymmetric, driven by the distinct microtubule‐binding specificities of its regulators: CLIMP63 binds to centrosomal microtubules, KTN1 prefers perinuclear polyglutamylated microtubules, and P180 associates with glutamylated microtubules, ultimately resulting in the pericentrosomal enrichment of ER sheets [8, 9].

Upon entering mitosis, the ER network undergoes extensive morphological remodeling [10, 11]. The ER membrane is excluded from the spindle zone during metaphase to prevent interference with chromosome segregation [12, 13, 14, 15, 16, 17]. However, how ER subdomains redistribute after mitotic entry, and in particular how the ER transitions from an asymmetric distribution in interphase to a relatively symmetric distribution around the cell equator, is still unclear. Moreover, although the interphase ER coordinates the dynamics of intracellular organelles [8], it is unknown whether mitotic ER remodeling influences the inheritance of other organelles.

Rab GTPases are key regulators of intracellular membrane trafficking [18]. They recruit effector proteins, including microtubule‐based motors such as cytoplasmic dynein and kinesins, to execute specific membrane trafficking functions [19]. Some endosomes marked by Rab GTPases, such as Rab5 and Rab7, mediate the rapid hitchhiking movement of ER tubules during interphase [20, 21, 22, 23, 24]. Although the role of Rab1A in modulating metaphase ER morphology has been reported [25], it remains to be determined whether Rab GTPases regulate ER partitioning during mitosis.

Here, we show that upon mitotic entry, the ER network assumes a distribution pattern opposite to that in interphase. Tubular ER proteins, especially RTN4, relocalize to the pericentrosomal region, forming a more tubular ER network around the centrosomes in prometaphase. By contrast, the ER membrane distal to the centrosomes becomes relatively flat and lamellar. Cyclin‐dependent kinase 1 (CDK1)‐mediated phosphorylation of RTN4 increases its interaction with Rab11 GTPase, facilitating dynein‐dependent transport of RTN4 to the pericentrosomal region. Mitotic RTN4 further promotes the symmetric distribution and equal inheritance of the ER and other organelles, including lysosomes and mitochondria, and is essential for proper mitotic progression. Thus, our study demonstrates the mechanism of ER symmetry remodeling during early mitosis and its implications for mitotic fidelity.

## 2 Results

### 2.1 ER‐Shaping Proteins Undergo Reverse Distribution Remodeling Upon Mitotic Entry, With RTN4 Enriched Around Centrosomes

**FIGURE 1:**
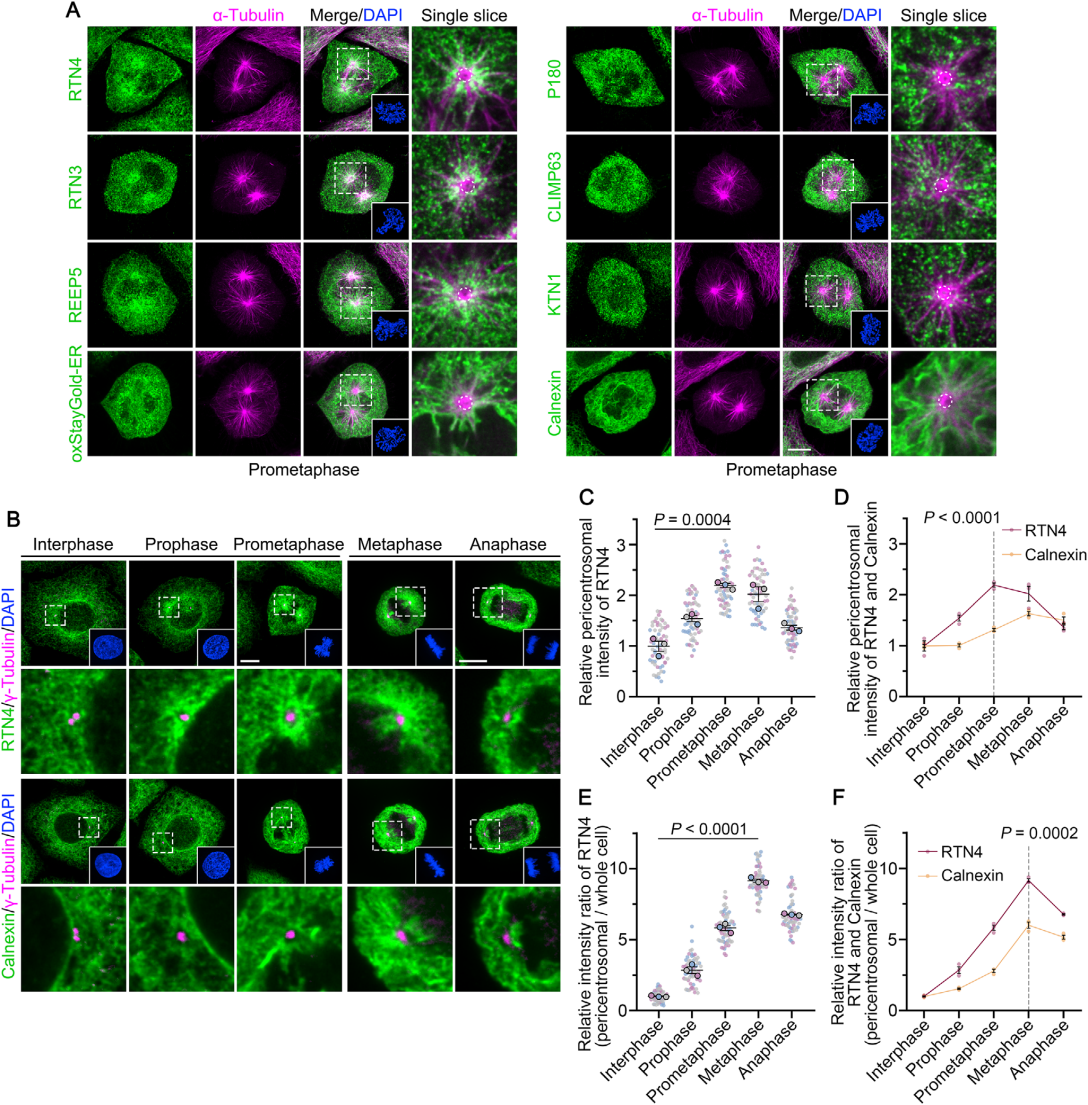
ER‐shaping proteins undergo reverse distribution remodeling during early mitosis, with RTN4 enriched around centrosomes. (A) Representative images of ER protein (green) distributions in prometaphase HeLa cells. For tubular ER proteins (RTN4, RTN3, REEP5), ER sheet proteins (CLIMP63, KTN1, P180), and the ER marker Calnexin, cells were immunolabeled with the indicated antibodies. For pan‐ER labeling, oxStayGold‐KDEL was stably expressed in HeLa cells. Cells were also immunolabeled for α‐tubulin (magenta, microtubules) and stained with DAPI (blue, DNA). Maximal‐intensity projections of *z*‐stacks are shown. Pericentrosomal regions (outlined) are enlarged on the right. Dashed circles outline the positions of the centrosomes. Scale bar, 10 µm. (B) Representative images of HeLa cells at different mitotic stages, immunolabeled for RTN4 (green, rows 1–2) or Calnexin (green, rows 3–4), respectively. Cells were also immunolabeled for γ‐tubulin (magenta, centrosomes) and stained with DAPI (blue). Pericentrosomal regions (outlined) are enlarged. The black line at the top of the image indicates the same magnification across panels. Scale bars, 10 µm. (C–F) Quantification of the pericentrosomal fluorescence intensity (C, D) and the pericentrosomal enrichment ratio (pericentrosomal/the whole cell fluorescence intensity) (E, F) of RTN4 and Calnexin at different stages of mitosis in (B). *n* = 3 independent experiments, with at least 64 cells analyzed per condition. Data points are color‐coded by biological replicates in (C) and (E). Data are presented as mean ± s.e.m. across replicates. Statistical tests were two‐tailed unpaired Student's *t*‐tests. *P* values are shown.

During interphase, tubular ER proteins (represented by RTN4 [6]) extended to the cell periphery, whereas ER‐sheet proteins (represented by CLIMP63 [7]) were mainly enriched near the nucleus and centrosome [8, 9] (Figure S1A). By contrast, upon entry into early mitosis, tubular ER proteins RTN4, RTN3, and REEP5 were enriched around centrosomes, while ER‐sheet proteins including CLIMP63, KTN1, and P180 were distributed away from mitotic centrosomes (Figure 1A). The ER marker Calnexin and oxStayGold‐KDEL [26], which are distributed throughout the ER network [7], showed no obvious preferential distribution in early mitosis (Figure 1A). The localization of these proteins in metaphase was similar to that in prometaphase, with RTN4 most prominently enriched at the spindle poles (Figure S1B). This reverse distribution relative to interphase was also observed in multiple cell types (Figure S1C,D), suggesting that early mitotic remodeling of the ER is a general mechanism.

Given that RTN4 accumulates most prominently around mitotic centrosomes, we examined its temporal dynamics throughout mitosis. During interphase, RTN4 was not enriched around the centrosome; however, it gradually accumulated in the pericentrosomal region after the onset of prophase (Figure 1B). The pericentrosomal intensity of RTN4 peaked at prometaphase, remained high until metaphase, and decreased after the onset of anaphase (Figure 1C–E). Although Calnexin also showed an increased pericentrosomal enrichment ratio in metaphase (Figure 1F), consistent with previous reports of total ER accumulation at spindle poles [13], it displayed no apparent enrichment around centrosomes in prometaphase (Figure 1D). These findings suggest that RTN4 is preferentially recruited to the pericentrosomal region during early mitosis. Live‐cell imaging further confirmed that, upon mitotic entry, RTN4 accumulated more around centrosomes compared to oxStayGold‐KDEL (Figure S1E,F and Video S1). Taken together, these results indicate that ER‐shaping proteins in early mitosis adopt a redistribution pattern opposite to that in interphase, with tubular ER proteins, particularly RTN4, markedly enriched around the mitotic centrosomes.

### 2.2 Mitotic Relocalization of RTN4 Shapes the Pericentrosomal Tubular ER Network and Facilitates Symmetric ER Distribution

**FIGURE 2:**
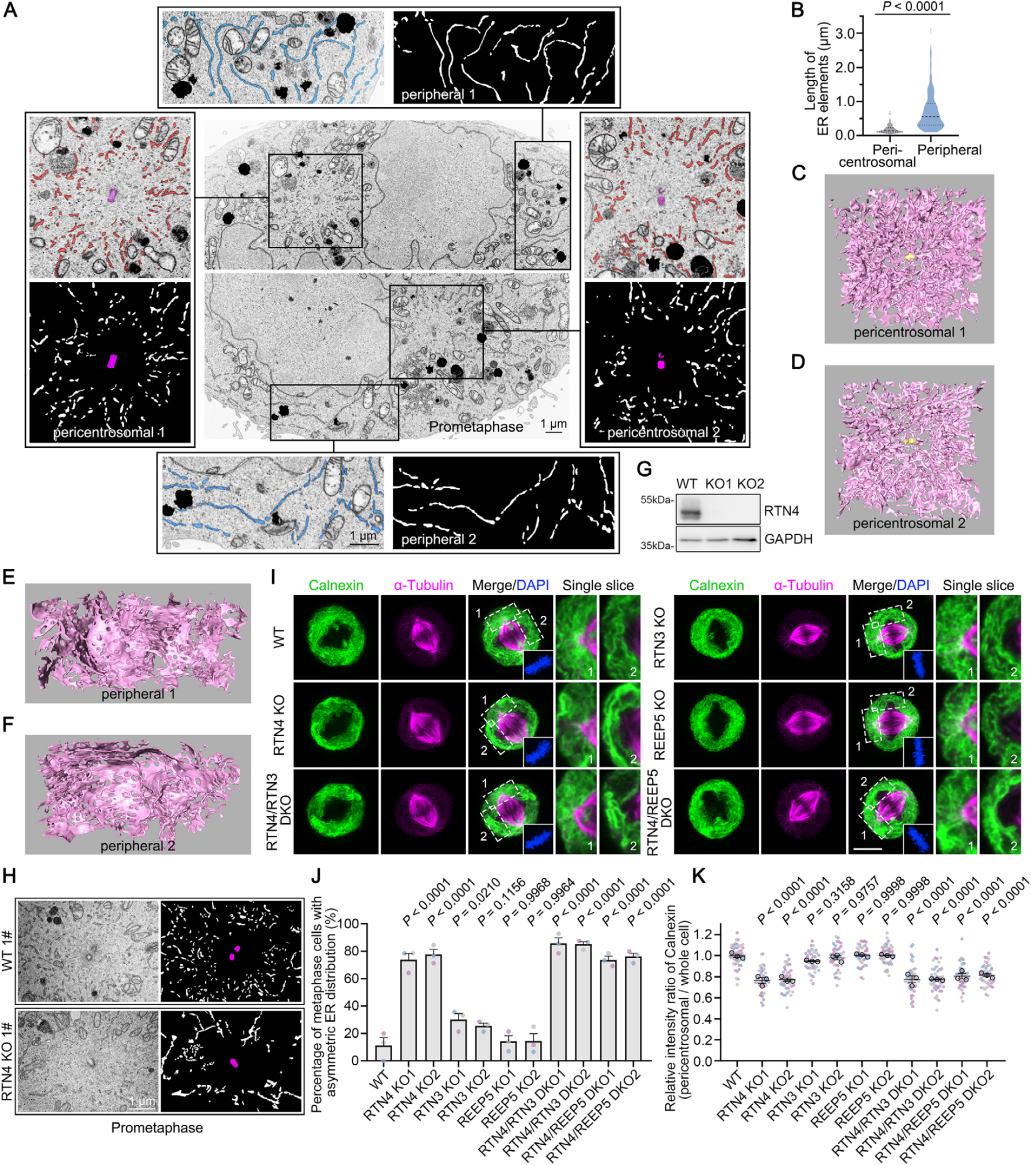
RTN4 pericentrosomal enrichment drives ER tubularization around centrosomes and promotes symmetric ER distribution during early mitosis. (A) Representative FIB‐SEM images of a HeLa cell in prometaphase. Two pericentrosomal regions and two regions distal to the centrosomes are enlarged. Pericentrosomal and peripheral ER are shown in red and blue, respectively. Centrosomes are marked in magenta. Scale bars, 1 µm. (B) Quantification of the lengths of pericentrosomal and peripheral ER elements in (A), with at least 134 ER elements counted per condition. (C–F) Three‐dimensional reconstructions of pericentrosomal (C, D) and peripheral ER (E, F) using FIB‐SEM data from (A). Centrosomes are marked in yellow. See also Video S2. (G) Western blot of wild‐type (WT) and RTN4 knockout (KO) HeLa cells. GAPDH served as the loading control. (H) Representative electron microscopy images of WT and RTN4 KO HeLa cells in prometaphase, showing centrosomes (magenta) and pericentrosomal ER (white). Scale bar, 1 µm. Additional examples are shown in Figure S2F. (I) Representative images of ER distribution in metaphase HeLa cells: WT, single knockouts (RTN4 KO, RTN3 KO, and REEP5 KO), and double knockouts (RTN4/RTN3 DKO and RTN4/REEP5 DKO). Cells were immunolabeled for Calnexin (green) and α‐tubulin (magenta). DNA was stained with DAPI (blue). Maximal‐intensity projections of *z*‐stacks are shown. ER regions (outlined) at the spindle pole (1) and equatorial plate (2) are enlarged on the right. Scale bar, 10 µm. (J) Proportion of WT and indicated KO cells with abnormal asymmetric ER distribution in (I). (K) Quantification of the pericentrosomal enrichment ratio (pericentrosomal/the whole cell fluorescence intensity) of Calnexin in (I). *n* = 3 independent experiments, with at least 40 cells analyzed per condition for (J) and (K). Data points are color‐coded by biological replicates. Data are presented as mean ± s.e.m. across replicates. Statistical tests were the Mann–Whitney test (B) and one‐way ANOVA (J, K). *P* values are shown.

Since the local concentration of RTN4 determines the morphology of the tubular ER in interphase [6, 27, 28], we inferred that RTN4 enrichment around the mitotic centrosomes promotes a more tubular pericentrosomal ER structure. We used focused ion beam‐scanning electron microscopy (FIB‐SEM) to analyze the ultrastructure of the ER membrane during early mitosis (Figure S2A). Individual SEM slices revealed that the cross‐sections of membrane elements surrounding the centrosomes were shorter than those located farther away (Figure 2A,B). Three‐dimensional (3D) reconstructions further showed that most observed membrane elements near the centrosomes were not discrete vesicles but ER components exhibiting *z*‐axis continuity (Figure 2C,D). Notably, the mitotic pericentrosomal ER displayed a more complex, tubular morphology, whereas the peripheral ER was flatter and resembled fenestrated ER sheets (Figure 2C–F and Video S2). The tubular ER surrounding the centrosomes in early mitosis formed an interconnected, radial 3D complex network with multidirectional branches (Figure 2C,D), distinct from the interphase peripheral tubular ER network that is serially joined by three‐way junctions [29]. Furthermore, the highly interconnected pericentrosomal tubular ER network, absent during interphase (Figure S2B), formed only upon mitotic entry, consistent with the spatiotemporal redistribution of RTN4 in early mitosis (Figure 1B). Therefore, the ER in early mitosis exhibits a distinct partitioning pattern from that in interphase, characterized by tubular ER enrichment around the centrosome, whereas sheet‐like ER is positioned farther away.

To further investigate the roles of tubule‐shaping proteins in ER reorganization during mitosis, we generated a series of RTN4, RTN3, and/or REEP5 single‐ and double‐knockout cell lines using CRISPR‐Cas9 approaches (Figure 2G and Figure S2C). Consistent with previous studies [30, 31], RTN4‐knockout interphase cells exhibited a reduced peripheral tubular ER network, accompanied by expanded ER sheets (Figure S2D). In early mitosis, RTN4 knockout disrupted the tubular ER network surrounding the centrosomes, resulting in sparse and uneven ER sheets in the pericentrosomal region (Figure 2H and Figure S2E–G), confirming that RTN4 enrichment drives pericentrosomal ER tubularization during early mitosis. More importantly, RTN4 deficiency produced substantial asymmetric ER distribution in metaphase cells, characterized by reduced ER accumulation around spindle poles and increased intensity of randomly distributed ER outside the spindle zone (Figure 2I–K). By contrast, single‐knockout of RTN3 or REEP5 did not cause such severe ER asymmetry in metaphase as RTN4 knockout (Figure 2I–K), demonstrating the indispensable role of RTN4 in remodeling symmetric ER distribution during mitosis. ER distribution abnormality in RTN4/RTN3 or RTN4/REEP5 double knockout cells resembled that of RTN4 single‐knockouts (Figure 2J,K). These mitotic ER aberrations in knockout cells were not secondary to changes in the levels of other ER‐shaping proteins (Figure S2C). Together, these data indicate that RTN4 enrichment toward the two separated centrosomes during early mitosis drives ER redistribution to both spindle poles, thereby establishing ER symmetry by metaphase.

We then assessed mitotic ER dynamics in early *Caenorhabditis elegans* embryos using the ER marker signal peptidase SP12 fused with mCherry [32]. During interphase, mCherry::SP12 localized to the nuclear envelope and was dispersed throughout the cytoplasmic ER membrane (Figure S2H), consistent with previous reports [32, 33]. As the embryo entered the first symmetric cell division, the ER clustered and accumulated around the centrosomes and nucleus, forming a prominent pericentrosomal ER enrichment similar to that observed in mammalian cells (Figure S2I). Moreover, loss of RET‐1, the homolog of RTN4 in nematodes, led to a marked decrease in the enrichment of the total ER content in the pericentrosomal area during prometaphase (Figure S2I–K), suggesting that the function of RTN4 in mitotic ER remodeling is evolutionarily conserved.

### 2.3 CDK1‐Mediated Phosphorylation Promotes RTN4 Relocalization During Mitosis

**FIGURE 3:**
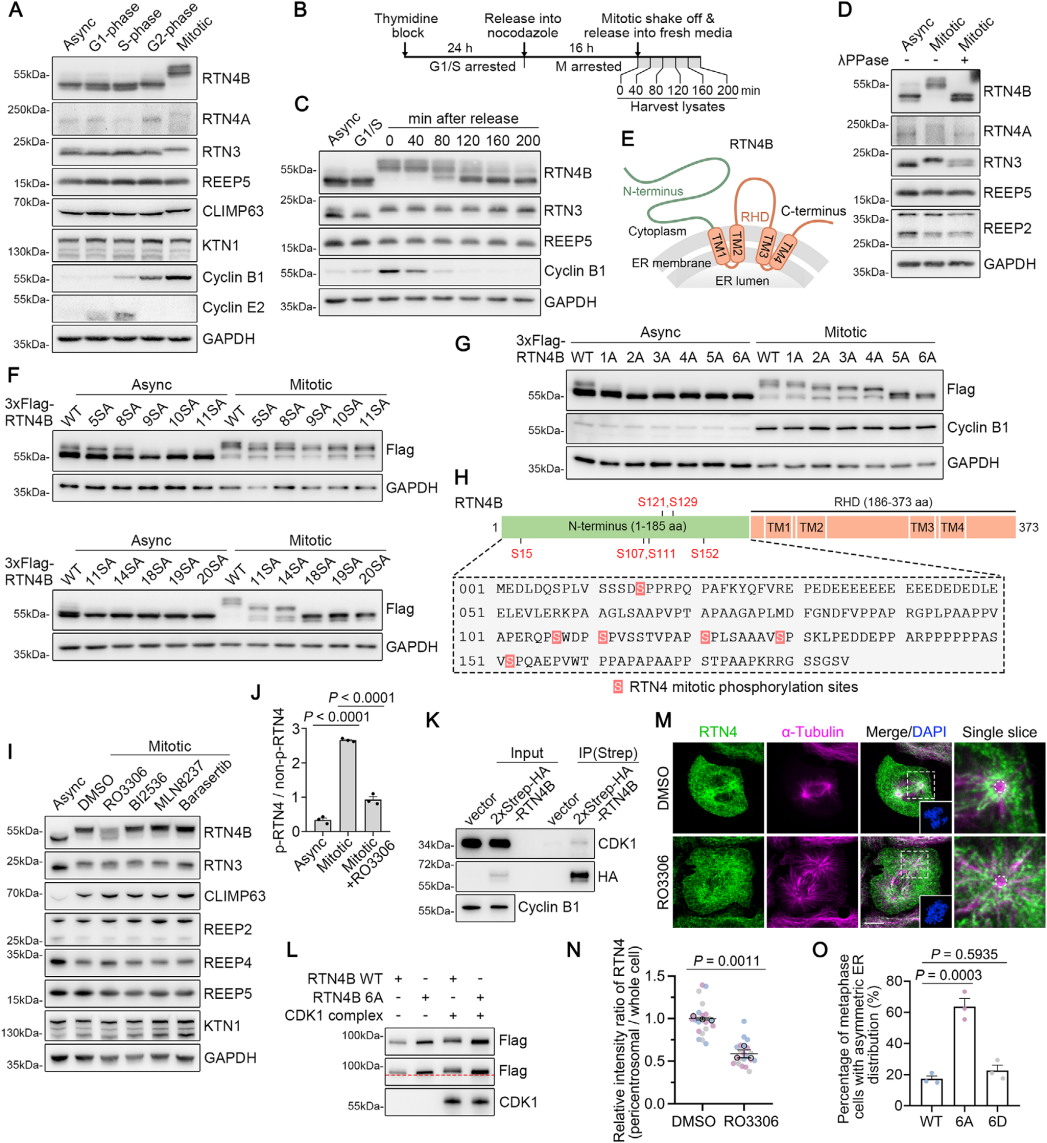
CDK1‐mediated phosphorylation promotes RTN4 redistribution during early mitosis. (A) Western blot analysis of lysates from synchronized HeLa cells treated with mimosine (G1 phase, 0.4 mm), aphidicolin (S phase, 2 µg/mL), RO3306 (G2 phase, 10 µm), or nocodazole (M phase, 100 ng/mL). Cyclin E2 and cyclin B1 are markers for S and M phases, respectively. GAPDH served as the loading control. Async, asynchronous cells. (B) Schematic of the mitotic synchronization protocol used in (C). (C) Western blot analysis of lysates from HeLa cells synchronized by thymidine‐nocodazole treatment and harvested at the indicated time points. (D) Whole‐cell lysates from asynchronous and mitotic HeLa cells were treated with λ‐phosphatase and analyzed by Western blot. (E) Schematic illustration of RTN4B domain composition. The N‐terminal cytoplasmic region is shown in green, and the RHD (RTN4 reticulon homology domain) is shown in orange. (F, G) HeLa cells transfected with the indicated RTN4B constructs were synchronized to prometaphase and analyzed by Western blot to detect the mobility shift of RTN4B mutants in mitosis. RTN4B 20SA denotes the mutant with all 20 N‐terminal serine residues substituted by alanine. 5SA: S7A, S11A, S12A, S13A, S15A; 8SA: 5SA + S181A, S182A, S184A; 9SA: 8SA + S107A; 10SA: 9SA + S152A; 11SA: 10SA + S64A; 14SA: 11SA + S111A, S114A, S115A; 18SA: 14SA + S121A, S124A, S129A, S131A; 19SA: 18SA + S150A; 20SA: 19SA + S171A. RTN4B 6A denotes the mutant with alanine substitutions at six serine residues (S15, S107, S111, S121, S129, and S152). 1A: S107A; 2A: 1A + S15A; 3A: 2A + S111A; 4A: 3A + S121A; 5A: 4A + S129A; 6A: 5A + S152A. (H) Six mitotic phosphorylation sites (red) and the N‐terminal 1–185 aa sequence of RTN4B. All six serine phosphorylation sites are in the N‐terminal cytoplasmic region. (I) Nocodazole‐arrested mitotic HeLa cells were treated with selected kinase inhibitors and analyzed by Western blot with the indicated antibodies. (J) Quantification of the ratio of phosphorylated RTN4 (p‐RTN4) intensity to non‐phosphorylated RTN4 (non‐p‐RTN4) intensity (three independent replicates). (K) Lysates from STLC‐arrested mitotic HeLa cells stably expressing 2×Strep‐HA‐RTN4B were immunoprecipitated with Strep‐Tactin XT resins, and interactions were evaluated by Western blot. (L) In vitro phosphorylation assay using the purified 3×Flag‐mEmerald‐RTN4B wild‐type (WT) or 6A mutant and the active CDK1/Cyclin B1 complex (GST‐tagged). Samples were subsequently analyzed by Western blot with the indicated antibodies. The second row shows the same blot as the first row. The red dashed line is a horizontal guide for comparing band migration levels. (M, N) Representative images (M) and quantification (N) of pericentrosomal RTN4 distribution in prometaphase HeLa cells treated with DMSO or CDK1 inhibitor RO3306. Cells were immunolabeled for RTN4 (green) and α‐tubulin (magenta) in (M). DNA was stained with DAPI (blue). Maximal‐intensity projections of *z*‐stacks are shown. Pericentrosomal regions (outlined) are enlarged on the right. Dashed circles outline the positions of the centrosomes. Scale bar, 10 µm. *n* = 3 independent experiments, with at least 20 cells analyzed per condition for (N). Data points are color‐coded by biological replicates. (O) The proportion of RTN4 knockout (KO) HeLa cells stably expressing 3×Flag‐mEmerald‐RTN4B WT, 6A, or 6D mutant with abnormal asymmetric ER distribution in Figure S3M. *n* = 3 independent experiments, with at least 49 cells analyzed per condition. Data in (J), (N), and (O) are presented as mean ± s.e.m. across replicates. Statistical tests were one‐way ANOVA (J, O) and two‐tailed unpaired Student's *t*‐test (N). *P* values are shown.

To investigate the mechanisms underlying RTN4 dynamics during the cell cycle, we first examined its protein levels at different cell cycle stages. While total RTN4 levels remained relatively constant throughout the cell cycle, a marked mobility upshift was specifically detected in prometaphase cells synchronized with nocodazole (Figure 3A). Upon nocodazole release and mitotic exit, the upshifted RTN4 bands gradually disappeared (Figure 3B,C). This prometaphase‐specific mobility shift was also confirmed in S‐trityl‐L‐cysteine (STLC, Eg5 inhibitor [34])‐arrested prometaphase cells and other cell types (Figure S3A) and was abolished by lambda protein phosphatase (λ‐PPase) treatment (Figure 3D), suggesting mitotic phosphorylation of RTN4.

We next sought to identify the mitotic phosphorylation sites of RTN4. Mammalian RTN4 comprises three major isoforms, RTN4A, RTN4B, and RTN4C, all of which share the same C‐terminal RHD region [35] (Figure S3B). Unlike RTN4C, RTN4A and RTN4B share the same 1–185 aa N‐terminal sequence, while RTN4A contains an additional isoform‐specific region (Figure S3B). Both RTN4A and RTN4B exhibited mitotic band shifts, whereas RTN4C and exogenous RHD did not (Figure S3C,D). Therefore, we focused on identifying phosphorylation sites within the N‐terminal 1–185 aa cytoplasmic region of RTN4B (Figure 3E). We generated a series of RTN4B mutants in which the 20 serine residues in the N‐terminal region were all or selectively mutated to alanine, and tested their mitotic mobility shift (Figure 3F and Figure S3E). Based on this, we identified six mitotic phosphorylation sites of RTN4 among the 20 serine residues, including S15, S107, S111, S121, S129, and S152 (Figure 3G,H), which were also confirmed by mass spectrometry (Figure S3F). The non‐phosphorylatable RTN4B 6A mutant abolished the mitotic band shift (Figure 3G), confirming the authenticity of these phosphorylation sites.

Most of the six mitotic serine phosphorylation sites in RTN4B reside within the consensus sequence for CDK1 (minimal site: S/T‐P; full site: S/T‐P‐x‐K/R [36]) (Figure 3H), a central kinase with diverse functions during mitotic entry [11]. Treatment of mitotic HeLa cells with various mitotic kinase inhibitors revealed that the CDK1‐specific inhibitor RO3306 reduced the upper mitotic bands of RTN4 (Figure 3I,J), and this effect was further validated by another CDK1 inhibitor, BMS‐265246 (Figure S3G). Co‐immunoprecipitation assays demonstrated that RTN4B interacts with CDK1 in mitosis (Figure 3K). We next performed an in vitro phosphorylation assay using purified RTN4B from HeLa cells (Figure S3H). The active CDK1–cyclin B1 complex induced a mobility shift in the wild‐type RTN4B but not in the 6A mutant (Figure 3L), suggesting that CDK1 phosphorylates RTN4B. The pericentrosomal accumulation of RTN4 in prometaphase was abolished after RO3306 or BMS‐265246 treatment (Figure 3M,N and Figure S3I,J). Furthermore, stable expression of either the non‐phosphorylatable RTN4B 6A mutant or the phosphomimetic RTN4B 6D mutant in RTN4‐knockout cells at levels comparable to endogenous RTN4B did not affect interphase ER morphology or distribution (Figure S3K,L). However, only RTN4B 6A‐expressing cells showed abnormal ER asymmetry in metaphase (Figure 3O and Figure S3M). Collectively, these results indicate that CDK1‐mediated phosphorylation of RTN4 is essential for its mitotic relocalization.

### 2.4 Rab11 Mediates the Mitotic Redistribution of RTN4 in a Dynein‐Dependent Manner

The transport of cargoes to the centrosome is primarily mediated by cytoplasmic dynein, which moves toward the minus end of microtubules [37]. Consistent with our previous report [8], RTN4 does not directly bind to microtubules (Figure S4A). However, microtubules were essential for RTN4 relocalization, as nocodazole‐induced microtubule depolymerization abolished pericentrosomal RTN4 localization (Figure S4B). By contrast, STLC treatment, which produces monopolar spindles without causing microtubule depolymerization [34], preserved pericentrosomal enrichment of RTN4 (Figure S4B,C). Treatment with dynarrestin, a cytoplasmic dynein inhibitor [38], or knockdown of p150^Glued^, a core component of the dynein‐dynactin complex [39], abolished RTN4 accumulation around the mitotic centrosomes (Figure S4D–G), suggesting that cytoplasmic dynein is required for the mitotic pericentrosomal enrichment of RTN4. However, we did not detect an interaction between RTN4 and the dynein heavy chain (Figure S4H), indicating that RTN4 undergoes mitotic redistribution through an indirect dynein‐dependent trafficking mechanism.

**FIGURE 4:**
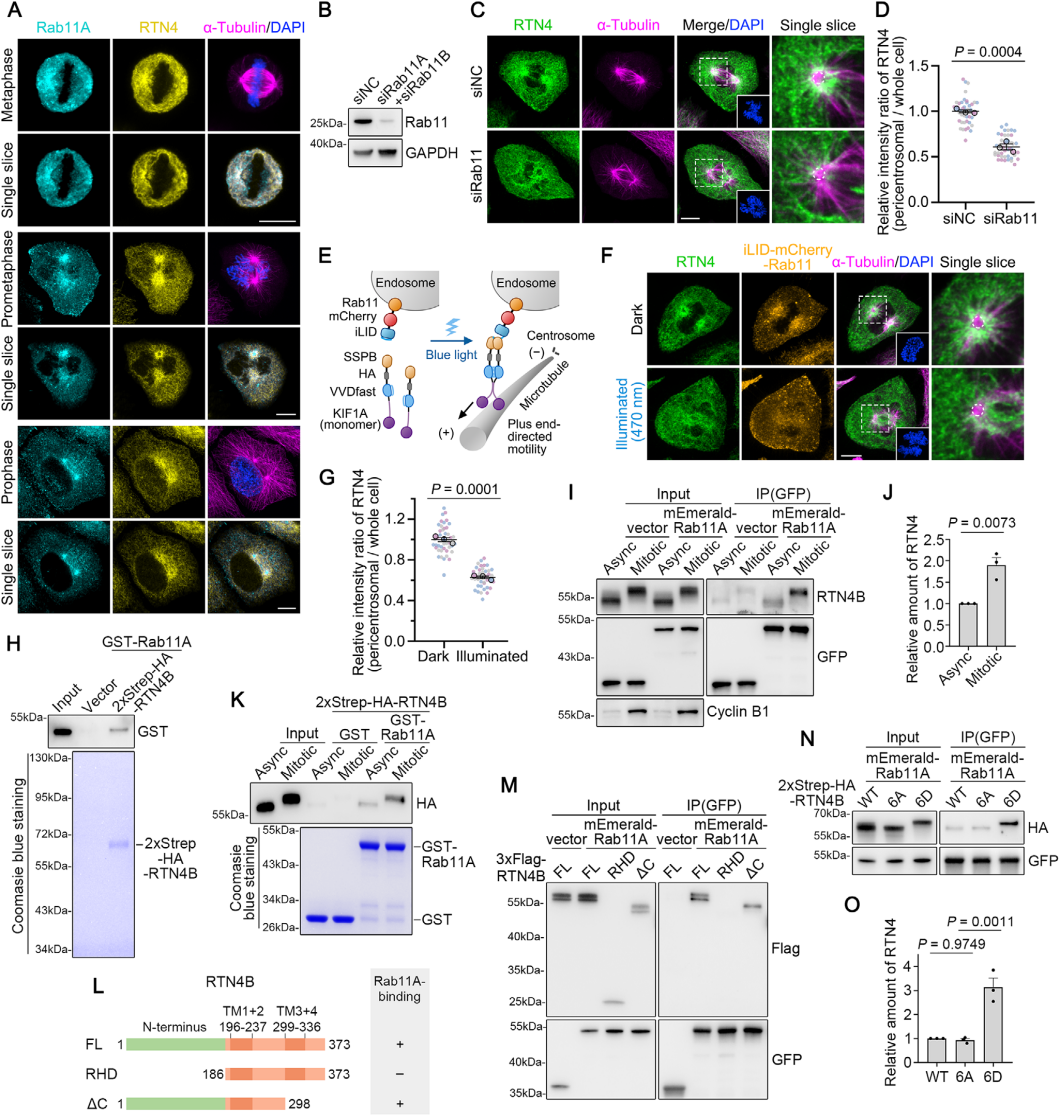
Rab11 mediates the relocalization of phosphorylated RTN4 during early mitosis. (A) Representative images of HeLa cells stably expressing mEmerald‐Rab11A (cyan) at different stages of mitosis. Cells were immunolabeled for RTN4 (yellow) and α‐tubulin (magenta). DNA was stained with DAPI (blue). Scale bars, 10 µm. (B) Western blot analysis of HeLa cells transfected with negative control (NC) or Rab11 siRNAs. GAPDH served as the loading control. (C, D) Representative images (C) and quantification (D) of pericentrosomal RTN4 distribution in prometaphase HeLa cells transfected with NC or Rab11 siRNAs. Cells were immunolabeled for RTN4 (green) and α‐tubulin (magenta) in (C). DNA was stained with DAPI (blue). Maximal‐intensity projections of *z*‐stacks are shown. Pericentrosomal regions (outlined) are enlarged on the right. Dashed circles outline the positions of the centrosomes. Scale bar, 10 µm. *n* = 3 independent experiments, with at least 41 cells analyzed per condition for (D). Data points are color‐coded by biological replicates. (E) Schematic representation of the optogenetic system for transporting and repositioning Rab11‐associated endosomes. Fluorescently labeled (mCherry) Rab11 was fused to iLID. Upon blue‐light (470 nm) illumination, two key dimerization events occur: the homodimerization of VVDfast and the heterodimerization of iLID with SSPB. This promotes both the activation of the opto‐kinesin (blue‐light‐sensitive KIF1A) and its binding to iLID‐mCherry‐Rab11, ultimately driving microtubule plus‐end‐directed transport of Rab11‐marked recycling endosomes. (F) Representative images of prometaphase HeLa cells expressing the indicated optogenetic constructs in the dark or after 10 min of blue‐light illumination. Cells were immunolabeled for RTN4 (green) and α‐tubulin (magenta). DNA was stained with DAPI (blue). Pericentrosomal regions (outlined) are enlarged on the right. Dashed circles outline the positions of the centrosomes. Scale bar, 10 µm. (G) Quantification of pericentrosomal RTN4 distribution in (F). *n* = 3 independent experiments, with at least 39 cells analyzed per condition. (H) In vitro binding assays of purified 2×Strep‐HA‐RTN4B and GST‐Rab11A. 2×Strep‐HA‐RTN4B were stained with Coomassie Brilliant Blue. (I) Lysates from STLC‐arrested mitotic HeLa cells stably expressing mEmerald‐Rab11A were immunoprecipitated with anti‐GFP nanobody agarose beads, and interactions were evaluated by Western blot. Cyclin B1 served as a marker for the M phase. (J) Quantification of the binding affinity of RTN4 for Rab11A from (I) (three independent replicates). (K) Lysates from asynchronous and STLC‐arrested mitotic HeLa cells stably expressing 2×Strep‐HA‐RTN4B were incubated with Glutathione Sepharose 4B beads pre‐coated with purified GST or GST‐Rab11A from Rosetta (DE3) cells. Samples were analyzed by Western blot with the indicated antibodies and Coomassie Brilliant Blue staining. (L) Schematic of full‐length (FL) RTN4B and its truncated mutants. Rab11A binding ability: +, positive; –, negative. (M) Lysates from HEK293T cells overexpressing mEmerald‐Rab11A and 3×Flag‐RTN4B (FL, RHD, or ΔC) were immunoprecipitated with anti‐GFP nanobody agarose beads, and interactions were evaluated by Western blot. (N) Lysates from HeLa cells stably expressing mEmerald‐Rab11A and co‐expressing either 2×Strep‐HA‐RTN4B wild‐type (WT), 6A, or 6D were immunoprecipitated with anti‐GFP nanobody agarose beads, and interactions were evaluated by Western blot. (O) Quantification of the binding affinity of RTN4B WT, 6A, or 6D for Rab11A from (N) (three independent replicates). Data in (D), (G), (J), and (O) are presented as mean ± s.e.m. across replicates. Statistical tests were the two‐tailed unpaired Student's *t*‐test (D, G, J) and one‐way ANOVA (O). *P* values are shown.

During interphase, some newly formed ER tubules are generated and transported by hitchhiking on highly motile organelles [24], such as Rab GTPase‐marked endosomes [20, 21, 22, 23], which are themselves transported along microtubules by molecular motors [19]. To examine the role of Rab GTPases in ER redistribution during mitosis, we first analyzed the mitotic localization of Rab1A/B, Rab5A, Rab7A, Rab11A/B, and Rab25 (Figure 4A and Figure S4I). Among these Rab proteins, Rab11 showed a centrosome‐directed distribution upon mitotic entry, consistent with a previous report [40]. During mitosis, pericentrosomal Rab11 partially colocalized with RTN4 (Figure 4A). Importantly, pericentrosomal enrichment of RTN4 in prometaphase was abolished after Rab11 depletion (Figure 4B–D). We next used a blue‐light‐induced optogenetic heterodimerization system [41] to generate a cell line stably expressing iLID‐mCherry‐Rab11 and a doxycycline‐inducible light‐sensitive kinesin (opto‐kinesin) construct KIF1A (1–365 aa)‐VVDfast‐HA‐SSPB (micro) (Figure 4E). Upon blue‐light illumination, Rab11 is transported toward the microtubule plus ends (Figure 4E). In interphase cells, both opto‐kinesins and Rab11 rapidly redistributed to the cell periphery after 10 min of illumination (Figure S5A), confirming the effectiveness of the system. RTN4 also accumulated at the cell periphery and partially colocalized with Rab11 in these cells (Figure S5A). When cells were synchronized in mitosis and globally illuminated with blue light, RTN4 was no longer enriched pericentrosomally during prometaphase (Figure 4F,G), further indicating that the centrosome‐directed redistribution of RTN4 in early mitosis is dependent on Rab11 activity.

### 2.5 RTN4 Phosphorylation Promotes its Interaction With Rab11 During Mitosis

We next examined whether RTN4 interacts with Rab11. Co‐immunoprecipitation assays revealed that among the three Rab11 family members, Rab11A, Rab11B, and Rab25, RTN4 preferentially interacted with Rab11A/B (Figure S5B). We further purified GST‐tagged Rab11A from Rosetta cells and 2×Strep‐HA‐tagged RTN4B from mitotic HeLa cells (Figure S5C). Recombinant Rab11A bound to RTN4B in vitro (Figure 4H), indicating a direct interaction between the two proteins. Moreover, the interaction between RTN4B and Rab11A increased during mitosis (Figure 4I,J), as further confirmed by GST pull‐down assays (Figure 4K). Mapping assays revealed that the N‐terminal cytoplasmic region of RTN4, which contains the mitotic phosphorylation sites, mediated its interaction with Rab11A (Figure 4L,M). We therefore inferred that mitotic phosphorylation of RTN4 increases its binding affinity for Rab11A. Compared with the RTN4B non‐phosphorylatable 6A mutant, the phosphomimetic 6D mutant interacted more strongly with Rab11A (Figure 4N,O). Consistently, treatment with a CDK1 kinase inhibitor impaired the interaction between RTN4B and Rab11A (Figure S5D). These results demonstrate that mitotic phosphorylation of RTN4 promotes its interaction with Rab11. Co‐immunoprecipitation assays showed that RTN4 interacts with FIP3, an adaptor protein that links Rab11 and dynein [40, 42], suggesting that FIP3 may be involved in the Rab11‐dynein‐mediated mitotic transport of RTN4 (Figure S5E). Furthermore, although RTN4 interacted with both the dominant‐negative (S25N) and constitutively active (Q70L) mutants of Rab11A (Figure S5F), overexpression of the S25N mutant reduced the pericentrosomal enrichment of RTN4 during prometaphase (Figure S5G,H). This indicates that the binding of RTN4 to Rab11 is independent of Rab11 activity, whereas the redistribution of RTN4 requires Rab11 activity, consistent with the inability of Rab11A S25N to recruit dynein and localize to the centrosomes during early mitosis [40].

### 2.6 RTN4 Redistribution Promotes Symmetric Organelle Inheritance

**FIGURE 5:**
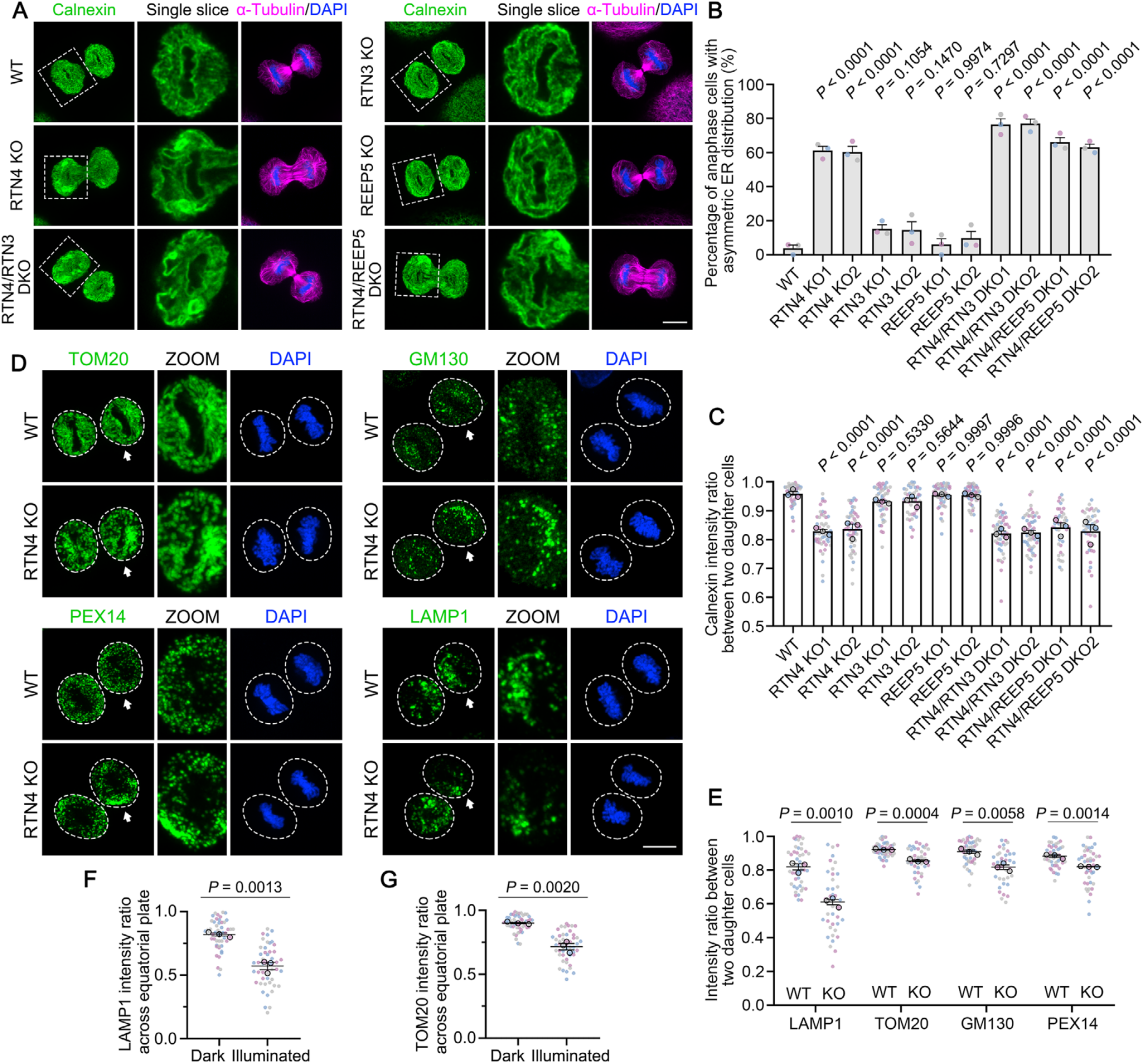
Knockout of RTN4 disrupts the symmetric inheritance of the ER and other organelles. (A) Representative images of ER partitioning in telophase HeLa cells: wild‐type (WT), single knockouts (RTN4 KO, RTN3 KO, and REEP5 KO), and double knockouts (RTN4/RTN3 DKO and RTN4/REEP5 DKO). Cells were immunolabeled for Calnexin (green) and α‐tubulin (magenta). DNA was stained with DAPI (blue). Maximal‐intensity projections of *z*‐stacks are shown. ER regions (outlined) are enlarged on the right. Scale bar, 10 µm. (B) Proportion of WT and indicated KO cells with abnormal asymmetric ER partitioning in (A). (C) Quantification of the Calnexin fluorescence‐intensity ratio between the two telophase daughter cells in (A). *n* = 3 independent experiments, with at least 44 cells analyzed per condition for (B) and (C). Data points are color‐coded by biological replicates. (D) Representative images of organelle (green) partitioning in telophase WT and RTN4 KO HeLa cells. Markers: anti‐TOM20 for mitochondria, anti‐LAMP1 for lysosomes, anti‐GM130 for Golgi apparatus, and anti‐PEX14 for peroxisomes. DNA was stained with DAPI (blue). Maximal‐intensity projections of *z*‐stacks are shown. The daughter cell regions (indicated by arrows) are enlarged on the right. Scale bar, 10 µm. (E) Quantification of the fluorescence‐intensity ratio of TOM20, GM130, PEX14, and LAMP1 between the two telophase daughter cells in (D). *n* = 3 independent experiments, with at least 37 cells analyzed per condition. (F, G) Quantification of the fluorescence‐intensity ratio between regions on either side of the equatorial plate in metaphase cells for LAMP1 (F) and TOM20 (G), from representative images in Figure S6D. *n* = 3 independent experiments, with at least 47 and 49 cells analyzed for (F) and (G), respectively. Data in (B), (C), (E), (F), and (G) are presented as mean ± s.e.m. across replicates. Statistical tests were one‐way ANOVA (B, C) and two‐tailed unpaired Student's *t*‐test (E–G). *P* values are shown.

To examine the effect of RTN4 mitotic redistribution on ER inheritance, we quantified the total ER intensity inherited by the two daughter cells at telophase. In wild‐type telophase cells, the two daughter cells inherited similar amounts of ER, whereas in RTN4‐knockout cells, significant differences were observed, leading to asymmetric ER partitioning (Figure 5A–C). By contrast, knockout of RTN3 or REEP5 did not cause similar ER inheritance defects (Figure 5A–C), highlighting the essential role of mitotic RTN4 in ensuring ER inheritance fidelity. Emerging evidence indicates that the interphase ER coordinates the positioning and transport of intracellular organelles through numerous membrane contact sites [3, 8, 24], and that morphological abnormalities of the mitotic ER may lead to defects in mitochondrial distribution [1]. We next examined the impact of RTN4‐mediated ER reorganization on the inheritance of other organelles. Some organelles, including lysosomes and mitochondria, clustered toward centrosomes during prophase but remained at the periphery of RTN4‐enriched regions (Figure S6A). RTN4 knockout caused asymmetry in the partitioning of lysosomes, mitochondria, peroxisomes, and the Golgi apparatus in telophase daughter cells (Figure 5D,E), demonstrating the importance of this ER‐shaping protein for the symmetric inheritance of organelles. When RTN4 was coupled to photoactivatable elements and forced to move toward microtubule plus ends by blue‐light‐activated KIF1A (Figure S6B), the interphase localization of lysosomes and mitochondria remained unaffected (Figure S6C); however, their symmetric distribution during metaphase was disrupted (Figure 5F,G and Figure S6D). Thus, these data indicate that RTN4 does not play a decisive role in ER‐mediated organelle positioning in interphase, but RTN4‐mediated ER reorganization promotes the redistribution and inheritance of other organelles during mitosis. Moreover, loss of RET‐1 in *C. elegans* led to uneven lysosome partitioning between the two daughter cells during the first symmetric embryonic cell division (Figure S6E,F), further suggesting the significance of mitotic ER dynamics for faithful organelle inheritance.

### 2.7 RTN4 Regulates Spindle Dynamics and Mitotic Progression

**FIGURE 6:**
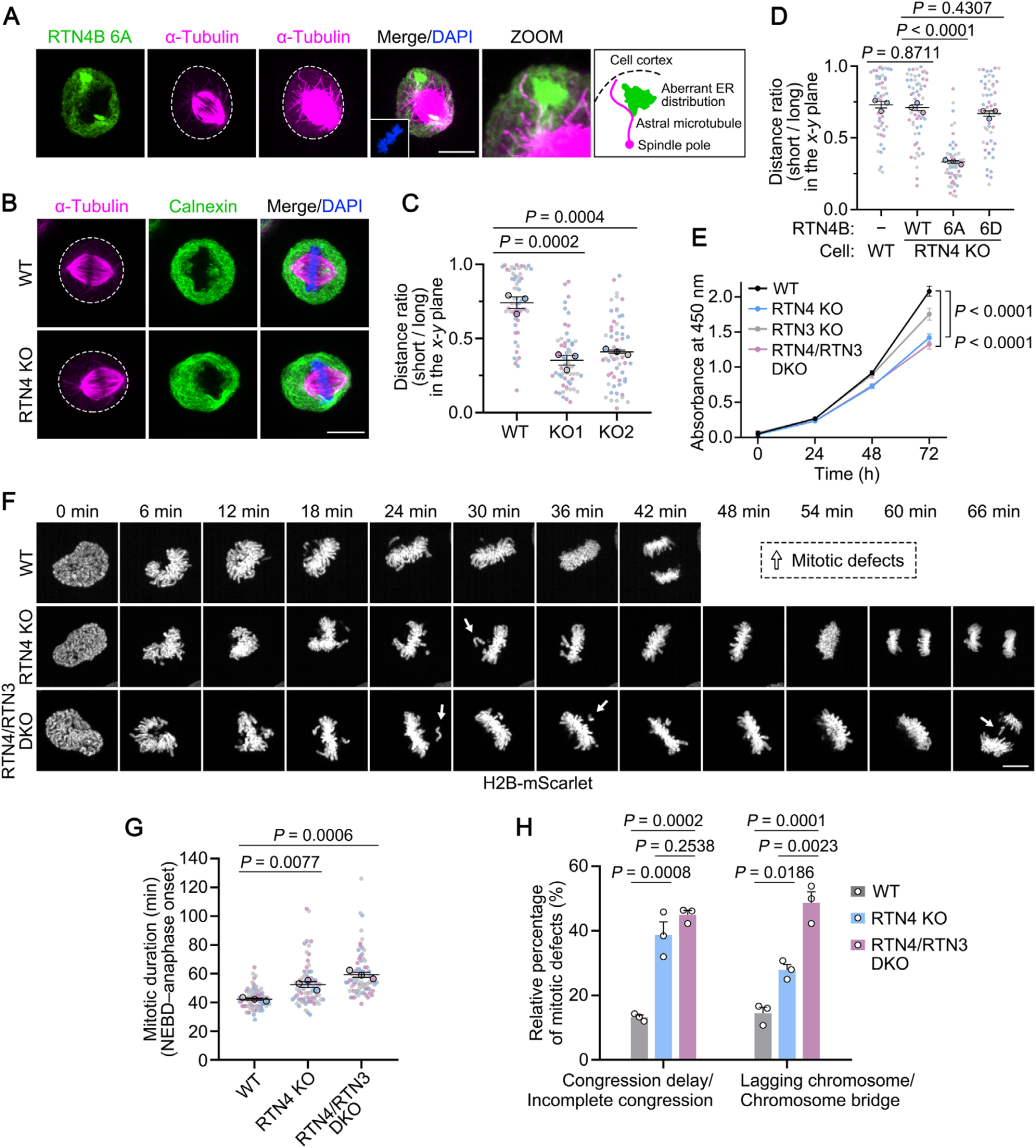
RTN4‐mediated ER mitotic remodeling facilitates spindle dynamics and mitotic progression. (A) Representative images of astral microtubules redirecting and bypassing abnormally distributed ER in HeLa cells stably expressing 3×Flag‐mEmerald‐RTN4B 6A (green). Cells were immunolabeled for α‐tubulin (magenta). DNA was stained with DAPI (blue). The abnormally distributed ER region (outlined) is enlarged, with a schematic diagram on the right. Scale bar, 10 µm. (B) Representative images of spindle positioning in the *x‐y* plane in wild‐type (WT) and RTN4 knockout (KO) metaphase HeLa cells. Cells were immunolabeled for Calnexin (green) and α‐tubulin (magenta). DNA was stained with DAPI (blue). Scale bar, 10 µm. (C) Quantification of metaphase spindle positioning in WT and RTN4 KO HeLa cells in (B). *n* = 3 independent experiments, with at least 54 cells analyzed per condition. (D) Quantification of metaphase spindle positioning in HeLa cells stably expressing 3×Flag‐mEmerald‐RTN4B WT, 6A, or 6D mutant. *n* = 3 independent experiments, with at least 49 cells analyzed per condition. (E) Cell growth rates of WT, RTN4 KO, RTN3 KO, and RTN4/RTN3 double knockout (DKO) HeLa cells were evaluated using the CCK‐8 assay. *n* = 6 experiments. Data are mean ± s.d. across replicates. (F) Time‐lapse images of WT, RTN4 KO, and RTN4/RTN3 DKO HeLa cells stably expressing H2B‐mScarlet (white). The time of nuclear envelope breakdown was set to zero; relative time (min) is shown. White arrows indicate mitotic defects. Scale bar, 10 µm. See also Video S3. (G) Quantification of the duration from nuclear envelope breakdown to anaphase onset in (F). (H) Quantification of the percentage of cells with mitotic defects in (F). *n* = 3 independent experiments, with at least 75 cells analyzed per condition for (G) and (H). Data in (C), (D), (G), and (H) are presented as mean ± s.e.m. across replicates. Statistical tests were one‐way ANOVA (C, D, E, G, H). *P* values are shown.

We next examined the effects of RTN4‐mediated ER reorganization during mitosis on spindle dynamics. The abnormally distributed ER caused by the expression of the RTN4B 6A mutant appeared to disrupt the orientation of astral microtubules in metaphase cells (Figure 6A), and the number of astral microtubules contacting the cell cortex from the two spindle poles also became unequal (Figure S7A,B), suggesting that astral microtubules may be unable to extend properly to the cortex. Compared with wild‐type cells, RTN4‐knockout metaphase cells exhibited spindle positioning defects, with one spindle pole deviating toward the cell cortex in the *x‐y* plane (Figure 6B,C and Figure S7C). This phenotype was also observed in other cell types during metaphase (Figure S7D–F) and could be rescued by re‐expression of the RTN4B 6D mutant, but not by the RTN4B 6A mutant (Figure 6D and Figure S3M), indicating that phosphorylation of RTN4, critical for ER mitotic remodeling, affects spindle positioning accuracy. The distribution of astral microtubules and NUMA proteins, which play decisive roles in spindle positioning [43], became asymmetric at the cell cortex because of the loss of RTN4 (Figure S7G,H). Furthermore, the spindle orientation angle (α) along the *z*‐axis increased after RTN4 knockout, with the average angle changing from 8.3° in wild‐type cells to 20.5° in RTN4‐knockout cells (Figure S7I–K). These findings underscore the importance of RTN4‐mediated ER remodeling in regulating spindle dynamics during mitosis.

The cell proliferation rate decreased after RTN4 knockout, as determined by the CCK‐8 assay (Figure 6E). We subsequently monitored the progression of cell division using live‐cell imaging. The duration from nuclear envelope breakdown to the onset of anaphase was extended in RTN4‐knockout and RTN4/RTN3 double‐knockout cells (Figure 6F,G and Video S3). Moreover, compared with wild‐type cells, both RTN4‐knockout and RTN4/RTN3 double‐knockout cells exhibited multiple mitotic defects, including incomplete congression, delayed congression, lagging chromosomes, and chromosome bridges (Figure 6F,H). We next examined whether RET‐1, the homolog of RTN4, contributes to the development of *C. elegans*. Compared with wild‐type animals, *ret‐1* loss‐of‐function mutants exhibited slower larval development and reduced body size (Figure S7L–O). Together, these results indicate that the ER‐shaping protein RTN4 contributes to proper mitotic progression and promotes development in *C. elegans*.

## 3 Discussion

**FIGURE 7:**
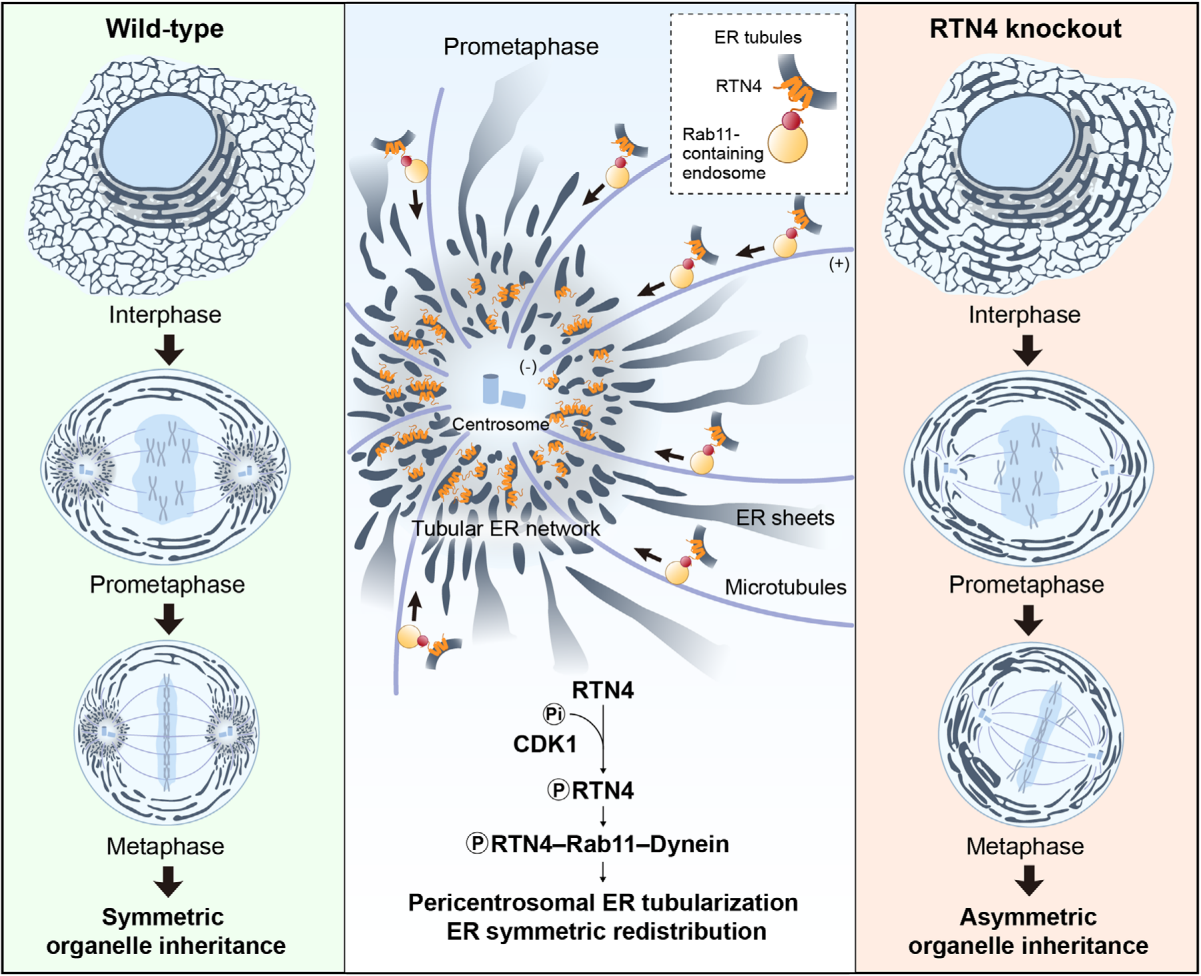
Model showing how RTN4 governs ER mitotic reorganization and facilitates symmetric organelle inheritance. Upon mitotic entry, RTN4 relocalizes to the pericentrosomal region, forming a more tubular ER network around the centrosomes. CDK1‐mediated phosphorylation of RTN4 increases its interaction with Rab11 GTPase, facilitating dynein‐dependent transport of RTN4 to the pericentrosomal region. RTN4‐mediated ER mitotic reorganization promotes the symmetric distribution and equal inheritance of the ER and other organelles and is essential for proper mitotic progression.

The ER is a pivotal orchestrator of cellular signaling and organelle positioning [3, 8]. Upon entering mitosis, it undergoes extensive morphological remodeling [25, 44, 45, 46, 47]. Key unresolved questions include how the ER achieves symmetric reorganization during early mitosis and whether this remodeling actively coordinates organelle segregation. In this study, we identify a fundamental mechanism of ER reorganization during early mitosis, whereby the ER undergoes not only positional rearrangement of its tubular and sheet‐like subdomains but also a transition from an asymmetric interphase distribution to a symmetric one concentrated around the two separate centrosomes (Figure 7). The tubular ER‐shaping protein RTN4 accumulates around the centrosomes during early mitosis, thereby driving the formation of a highly branched tubular ER network in the pericentrosomal region. As the centrosomes define the two poles of symmetric cell division, this redistribution ensures the symmetric partitioning and inheritance of the mitotic ER. We also demonstrate that the RTN4 homolog plays a conserved role in mediating mitotic ER redistribution in *C. elegans*. Consistently, a comparable pericentrosomal ER enrichment is also evident in dividing *Drosophila* syncytial embryos [48]. Notably, RTN4 knockout does not completely abolish pericentrosomal ER during early mitosis, despite changes in its density and morphology. This may reflect interphase ER sheets originally near the centrosome that failed to be properly redistributed upon mitotic entry. Nevertheless, we do not exclude the possibility that additional factors also promote ER transport to or anchoring at the mitotic centrosome.

Faithful organelle inheritance is a defining feature of symmetric cell division, and its failure can disrupt cell fate, leading to developmental disorders and tumorigenesis [1, 2]. Our previous work demonstrated that the ER acts as a master regulator of spatial rearrangements of other membranous organelles, including mitochondria and lysosomes, during interphase [8]. Here, we find that RTN4‐mediated ER reorganization during mitosis facilitates the symmetric distribution and inheritance of other organelles between the two daughter cells, likely via membrane contact sites that connect the ER to other organelles [1, 49]. Therefore, rather than equipping each discrete organelle with a distinct mitotic redistribution mechanism, cells use the continuous, integrated ER membrane as a unified transport platform to achieve organizational efficiency in rapid organelle repositioning during mitosis. Moreover, ER sheet‐associated proteins (CLIMP63, P180, and KTN1) are organizers for the distribution of the ER and other organelles during interphase [8]. However, during mitosis, this organizational role shifts to the tubular ER protein RTN4. This transition may result from the functional inactivation of specific ER‐sheet proteins during mitosis [14], preventing their strong microtubule‐binding activity from interfering with spindle assembly.

Rab proteins coordinate vesicle and organelle transport [18]. Specific ER‐associated Rab GTPases, including Rab5 and Rab7, regulate the morphology and dynamics of ER tubules in interphase cells [20, 21, 22, 23, 50]. During mitosis, degradation of the Rab11 GTPase‐activating protein (GAP) Evi5 increases the level of active, GTP‐bound Rab11, which in turn promotes Rab11 recruitment to the mitotic centrosomes [40]. Our results further demonstrate that upon mitotic entry, Rab11 facilitates dynein‐dependent relocalization of RTN4 to the pericentrosomal region, revealing a novel role of Rab proteins in mediating ER symmetric redistribution during mitosis. This mechanism is consistent with the function of Rab11 in regulating ER morphology in *C. elegans* [51]. Although RTN4 binds both the constitutively active Rab11A Q70L and dominant‐negative Rab11A S25N mutants, expression of the S25N mutant reduces pericentrosomal RTN4 accumulation during prometaphase (Figure S5G,H), consistent with the inability of this mutant to recruit dynein and localize to the centrosome [40].

After mitotic entry, several ER membrane proteins are phosphorylated and dissociate from the spindle microtubules [14, 15]. Here, we find that RTN4, a protein that does not directly bind microtubules, is phosphorylated during mitosis; this modification promotes its microtubule‐dependent reorganization. This finding broadens the known role of ER protein phosphorylation in early mitosis. Furthermore, CDK1, a core mitotic regulator that triggers mitotic entry [52], mediates multisite phosphorylation of its substrates [36]. We demonstrate that CDK1 phosphorylates RTN4 on multiple serine residues within its N‐terminal cytoplasmic region, the same region responsible for Rab11 binding. Since our in vitro phosphorylation assay does not fully recapitulate RTN4 phosphorylation in mitosis, we cannot exclude the possible contribution of additional kinases, which warrants further investigation.

## 4 Experimental Section

### 4.1 Cell Culture

HeLa, U2OS, COS‐7, RPE1, and HEK293T cells were obtained from the ATCC. HeLa, U2OS, COS‐7, and HEK293T cells were cultured in Dulbecco's Modified Eagle Medium (DMEM; high glucose, CellMax, CGM101.06) supplemented with 10% (v/v) fetal bovine serum (FBS, CellMax, SA201.02). RPE1 cells were cultured in DME/F12 (1:1) medium (CellMax, CGM104.06) supplemented with 10% FBS. All cells were maintained in a humidified incubator at 37°C with 5% CO_2_.

### 4.2 Plasmid Construction

Human RTN4 (GenBank: NM_153828.3), Rab11A (GenBank: NM_004663.5), Rab11B (GenBank: NM_004218.4), Rab1A (GenBank: NM_004161.5), Rab1B (GenBank: NM_030981.3), Rab5A (GenBank: NM_004162.5), Rab7A (GenBank: NM_004637.6), Rab25 (GenBank: NM_020387.4), RAB11FIP3 (GenBank: NM_014700.4), and H2BC11 (GenBank: NM_021058.4) were amplified from a U2OS cell cDNA library and cloned into p3×Flag‐CMV‐7.1 (Sigma–Aldrich, E4026), pmEmerald‐C1 (Addgene, 53975), pmScarlet‐C1 (Addgene, 85042), pcDNA3.1(+) (Invitrogen, V790‐20), p2×StrepII‐mNeonGreen [8], pSIN (provided by Zhengfan Jiang, Peking University, China), and/or pGEX‐6P‐1 (Amersham Biosciences, 27‐4597‐01) using standard cloning procedures. RTN4 phosphorylation site mutants, RTN4 truncation mutants, Rab11A S25N, and Rab11A Q70L mutants were constructed by PCR and seamless cloning (2×MultiF Seamless Assembly Mix, ABclonal, RK21020). pcDNA3‐er‐(n2)oxStayGold(c4) v2.0 was obtained from Addgene (186296). pSIN‐iLID‐mCherry‐Rab11 and pLVX‐KIF1A (1–365 aa)‐VVDfast‐HA‐SSPB (micro) were generated from pB80‐KIF1A (1–365 aa)‐VVDfast‐mVenus‐SSPB (micro)_P2A_iLID‐mCherry‐Rab11 (Addgene, 174644) and cloned into pSIN and pLVX (provided by Zhe Zhang, Peking University, China), respectively.

### 4.3 *C. Elegans* Strains and Maintenance

Worms were maintained on nematode growth medium (NGM) plates seeded with *E. coli* strain OP50‐1 as a food source at 20°C. *E. coli* OP50‐1 was cultured overnight in LB broth containing 10 mg/L streptomycin at 37°C and then seeded onto NGM plates to grow for two days at room temperature. The following *C. elegans* strains were obtained from the Caenorhabditis Genetics Center: N2, VC441: *ret‐1(gk242)* V, OCF15: *unc‐119(ed3)* III*; ocfIs2 [pie‐1p:mCherry::sp12::pie‐1 3'UTR + unc‐119(+)]*, and RW10226: *unc‐119(ed3) III; ltIs37 [pie‐1p::mCherry::his‐58 + unc‐119(+)] IV; stIs10226 [his‐72p::HIS‐24::mCherry::let‐858 3' UTR + unc‐119(+)]*. xdKi18 (gfp::rab‐7 knock‐in) [53] was a kind gift from Dr. Mei Ding at the Institute of Genetics and Developmental Biology, Chinese Academy of Sciences, Beijing, China. In all experiments, *ret‐1(lf)* mutants were matched with the N2 strain used for outcrossing. The presence of individual mutants and transgenes was confirmed by PCR and sequencing.

### 4.4 Antibodies

Primary antibodies used: rabbit polyclonal anti‐ATL3 (Proteintech, 16921‐1‐AP), rabbit polyclonal anti‐Calnexin (Proteintech, 10427‐2‐AP), rabbit polyclonal anti‐CDK1 (Proteintech, 19532‐1‐AP), rabbit polyclonal anti‐CKAP4 (Climp63) (Proteintech, 16686‐1‐AP), mouse monoclonal anti‐Climp63 (Enzo, ENZ‐ABS669), rabbit polyclonal anti‐Cyclin B1 (Proteintech, 28603‐1‐AP), rabbit polyclonal anti‐Cyclin E2 (Cell Signaling Technology, 4132), rabbit polyclonal anti‐DYNC1H1 (DHC1) (Proteintech, 12345‐1‐AP), mouse monoclonal anti‐EEA1 (BD Biosciences, 610456), mouse monoclonal anti‐Flag M2 (Sigma–Aldrich, F1804), mouse monoclonal anti‐GAPDH (Proteintech, 60004‐1‐Ig), rabbit polyclonal anti‐GFP (This lab [54], N/A), rabbit monoclonal anti‐GM130 (Abcam, ab52649), mouse monoclonal anti‐GST (Proteintech, 66001‐2‐Ig), mouse monoclonal anti‐HA (Sigma–Aldrich, H9658), rabbit polyclonal anti‐KTN1 (Proteintech, 19841‐1‐AP), mouse monoclonal anti‐LAMP1 (Santa Cruz, sc‐20011), rabbit polyclonal anti‐Lunapark (Abcam, ab121416), rabbit polyclonal anti‐NUMA (Abcam, ab97585), mouse monoclonal anti‐p150 [Glued] (BD Biosciences, 610473), rabbit polyclonal anti‐p180 (Thermo Fisher Scientific, PA5‐21392), rabbit polyclonal anti‐PEX14 (Proteintech, 10594‐1‐AP), rabbit polyclonal anti‐Rab11A/B (Proteintech, 15903‐1‐AP), rabbit polyclonal anti‐REEP2 (Proteintech, 15684‐1‐AP), rabbit polyclonal anti‐REEP4 (Proteintech, 26650‐1‐AP), rabbit polyclonal anti‐REEP5 (Proteintech, 14643‐1‐AP), rabbit polyclonal anti‐Reticulon3 (Proteintech, 12055‐2‐AP), rabbit polyclonal anti‐Reticulon4 (Novusbio, NB100‐56681), rabbit polyclonal anti‐Reticulon4 (Proteintech, 10740‐1‐AP), rabbit polyclonal anti‐TOM20 (Proteintech, 11802‐1‐AP), mouse monoclonal anti‐TOM20 (BD Biosciences, 612278), mouse monoclonal anti‐α‐tubulin (Sigma–Aldrich, T5168), rabbit monoclonal anti‐α‐tubulin (Abcam, ab176560), mouse monoclonal anti‐γ‐tubulin (Sigma–Aldrich, T5326), rabbit polyclonal anti‐γ‐tubulin (Sigma–Aldrich, T3559). Peroxidase‐AffiniPure goat anti‐ mouse/rabbit IgG (H+L) secondary antibodies were from Jackson ImmunoResearch. Alexa Fluor 488/568/647 conjugated goat anti‐mouse/rabbit IgG (H+L) highly cross‐adsorbed secondary antibodies were from Invitrogen.

### 4.5 Knockout Cell Line Generation by CRISPR/Cas9

All knockout cell lines were generated using CRISPR/Cas9‐based methods. Target oligonucleotides (5’‐GCGGGCACGGTCGACGACAC‐3’ and 5’‐CGTTCAAGTACCAGTTCGTG‐3’ for RTN4, 5’‐GCGCGCCTTACCCGCACAGG‐3’ and 5’‐CTGTGCGGGTAAGGCGCGCG‐3’ for RTN3, 5’‐GGAGCTTCATCGCTCTTGGT‐3’ and 5’‐CGGACTGGTGGCCTTGTACC‐3’ for REEP5) were synthesized, ligated into U6‐sgRNA vectors, and co‐transfected with pSpCas9. Transfected cells were selected using 2 µg/mL puromycin (Selleck, S7417) or 10 µg/mL blasticidin (Selleck, S7419) and single‐cell cloned by flow cytometry (Beckman Coulter, MoFlo Astrios EQ). Expanded clones were validated by Western blot analysis and genome sequencing to confirm successful knockout.

### 4.6 Cell Transfection and Chemical Treatment

Lipofectamine 3000 (Invitrogen, L3000015) and polyethylenimine (Polysciences, 23966) were used for plasmid transfection according to the manufacturer's instructions.

For cell synchronization, HeLa cells were treated with 0.4 mm mimosine (Selleck, S7446), 2 µg/mL aphidicolin (Sigma–Aldrich, 178273), 10 µm RO3306 (Sigma–Aldrich, SML0569), or 100 ng/mL nocodazole (Sigma–Aldrich, M1404) for 16 h to synchronize cells in late G1, early S, G2, and G2/M phases, respectively. For prometaphase arrest, cells were treated with 10 µm STLC (Sigma–Aldrich, 164739) for 16–18 h.

For imaging prometaphase‐metaphase cells, HeLa cells were treated with 2.5 mm thymidine (Sigma–Aldrich, T1895) for 24 h, washed three times with fresh medium, and released into the indicated phases before processing for immunofluorescence. To assess RTN4 phosphorylation during mitotic exit, synchronized cells were released from a thymidine block and treated with 100 ng/mL nocodazole for 16 h to induce mitotic arrest. The mitotic rounded cells were then released from nocodazole, and lysates were collected every 40 min.

For dephosphorylation assays, asynchronous or nocodazole‐arrested mitotic cell lysates were treated with λPPase (NEB, P0753) for 30 min at 30°C according to the manufacturer's instructions. Samples were then analyzed by Western blotting.

To inhibit mitosis‐related kinases, nocodazole‐arrested mitotic HeLa cells were treated with 500 nm MLN8237 (Selleck, S1133) for Aurora kinase A inhibition, 10 µm Barasertib (Selleck, S1147) for Aurora kinase B inhibition, or 100 nm BI 2536 (Selleck, S1109) for PLK1 inhibition, each for 30 min. To inhibit CDK1, RO3306 was used at 10 µm for 15–30 min, or BMS‐265246 (Selleck, S2014) was used at 1 µm for 30–40 min. Dynein activity was inhibited using 50 µm dynarrestin (MedChemExpress, HY‐121802) for 1 h.

### 4.7 Stable Cell Lines

For lentivirus production, HEK293T cells were co‐transfected with the packaging plasmids psPAX2 and pCMV‐VSV‐G, together with the indicated transfer plasmids using polyethylenimine. 6 h after transfection, the culture medium was replaced with fresh medium. Lentiviral supernatant was collected 48 h after medium replacement, filtered through a 0.22 µm filter unit (Millipore), and concentrated with 10% (w/v) PEG8000. For stable cell line generation, target cells were infected with the packaged lentivirus in the presence of 8 µg/mL polybrene (Sigma–Aldrich, 107689). Cells stably expressing the desired recombinant proteins were selected and maintained with 2 µg/mL puromycin and/or sorted by fluorescence using flow cytometry (Beckman Coulter, MoFlo Astrios EQ).

### 4.8 RNA Interference

The sense‐strand sequence of the negative control siRNA was 5’‐UUCUCCGAACGUGUCACGUTT‐3’. The human Rab11A siRNA target sequence was 5’‐AAUGUCAGACAGACGCGAAAA‐3’. The human Rab11B siRNA target sequence was 5’‐AAGCACCUGACCUAUGAGAAC‐3’. These siRNA oligonucleotides effectively knock down Rab11A and Rab11B when co‐transfected into cells [55]. The p150^Glued^ siRNA target sequence was 5’‐GAUCGAGAGACAGUUAUUA‐3’ [39]. All siRNAs were synthesized by GenePharma. Cells were transfected with siRNAs using CALNP RNAi in vitro reagent (D‐Nano Therapeutics, DN001) for 48 h according to the manufacturer's instructions.

### 4.9 Western Blotting and Co‐Immunoprecipitation

For Western blotting, cells were directly lysed in sample loading buffer (50 mm Tris‐HCl, 10% glycerol, 2% SDS, 0.1% bromophenol blue, 1 mm DTT, pH 6.8), boiled for 10 min, and sonicated for 5 min. Proteins were separated by SDS‐PAGE and transferred to polyvinylidene difluoride membranes (Millipore, IPVH00010). The membranes were blocked with 4% (w/v) non‐fat milk and incubated with the indicated primary antibodies, followed by HRP‐conjugated secondary antibodies. Immunoreactive proteins were visualized using the SuperSignal Enhancer Chemiluminescent (ECL Plus) Substrate (Nature Biosciences, TE0015) and imaged with a Tanon 5200 Multi chemiluminescence imaging system. Primary antibodies were used at the following dilutions: mouse monoclonal anti‐Flag M2 (Sigma–Aldrich, F1804), 1:10000; mouse monoclonal anti‐GAPDH (Proteintech, 60004‐1‐Ig), 1:10000; mouse monoclonal anti‐HA (Sigma–Aldrich, H9658), 1:5000; rabbit polyclonal anti‐Reticulon4 (Novusbio, NB100‐56681), 1:2000; mouse monoclonal anti‐α‐tubulin (Sigma–Aldrich, T5168), 1:5000. All other antibodies were used at 1:1000.

For co‐immunoprecipitation analysis, transfected HEK293T cells or HeLa cells stably expressing exogenous proteins were collected and lysed on ice for 20 min in immunoprecipitation lysis buffer (50 mm Tris‐HCl, 150 mm NaCl, 1% Triton X‐100, 1 mm EDTA, pH 7.4), supplemented with protease and phosphatase inhibitor cocktails. After centrifugation, the supernatants were incubated with anti‐Flag M2 affinity gels (Millipore, A2220), anti‐GFP nanobody agarose beads (AlpalifeBio, KTSM1301), or Strep‐Tactin XT 4Flow resins (IBA Lifesciences, 2‐5010‐010) for 2 h at 4°C. After washing with lysis buffer, the immunoprecipitated samples were boiled in SDS sample buffer and analyzed by Western blotting.

### 4.10 Immunofluorescence and Live Cell Imaging

For immunofluorescence, cells grown on coverslips were fixed with 4% (w/v) paraformaldehyde (PFA) for 15 min at 37°C and permeabilized with 0.15% Triton X‐100 in PBS for 5 min. After blocking in PBS containing 4% (w/v) BSA, cells were incubated with primary antibodies overnight at 4°C, followed by Alexa Fluor‐conjugated, species‐specific secondary antibodies for 1 h at room temperature. DNA was stained with 1 µg/mL DAPI (Invitrogen, D1306). Primary antibodies were used at the following dilutions: mouse monoclonal anti‐Flag M2 (Sigma–Aldrich, F1804), 1:1000; mouse monoclonal anti‐HA (Sigma–Aldrich, H9658), 1:1000; mouse monoclonal anti‐LAMP1 (Santa Cruz, sc‐20011), 1:500; rabbit polyclonal anti‐TOM20 (Proteintech, 11802‐1‐AP), 1:1000; and mouse monoclonal anti‐α‐tubulin (Sigma–Aldrich, T5168), 1:1000. All other antibodies were used at 1:200. Cells were mounted with Fluoromount‐G (SouthernBiotech, 0100–01) and imaged using either a Live SR CSU W1 spinning disk confocal microscope equipped with a 100×/1.4 NA oil objective lens or a Zeiss LSM980 confocal microscope in Airyscan mode equipped with a 63×/1.4 NA Plan‐Apochromat oil‐immersion objective. Images were processed using Imaris (Oxford Instruments, version 9.6) or ImageJ (National Institutes of Health) software.

For live‐cell imaging, cells were seeded in 35‐mm glass‐bottom dishes and maintained in a chamber at 37°C with 5% CO_2_ during imaging. Live‐cell imaging was performed using a Dragonfly high‐speed spinning‐disk confocal microscope (Andor) equipped with a 63×/1.4 NA oil‐immersion objective. Microtubules were visualized using SiR‐Tubulin (Cytoskeleton, CY‐SC002) following the manufacturer's instructions.

For *C. elegans* embryo imaging, dissected embryos were mounted as described previously [32] and imaged using a Live SR CSU W1 spinning‐disk confocal microscope equipped with a 60×/1.27 NA water‐immersion objective.

### 4.11 Measurements of Pericentrosomal ER Distribution

To assess mitotic pericentrosomal ER distribution, HeLa cells were immunolabeled with anti‐RTN4 or anti‐Calnexin and imaged using a Live SR CSU W1 spinning‐disk confocal microscope equipped with a 100×/1.4 NA oil‐immersion objective. Maximum‐intensity *z*‐projections were generated from the acquired three‐dimensional image stacks before quantification. The total fluorescence intensity of RTN4 or Calnexin within two 6‐µm‐diameter circular pericentrosomal regions (centered on the two mitotic centrosomes) was quantified manually in Fiji software and expressed as the ratio to the total cellular fluorescence intensity.

To quantify ER accumulation around the centrosome in mitotic *C. elegans* embryos, the fluorescence intensity of SP12 within an 8‐µm‐diameter circular pericentrosomal region (centered on the mitotic centrosome at a relatively low *z*‐position in the AB cell) was manually measured using Fiji and expressed as the ratio to that of an equivalent cortical area (a circular area of equal size at the AB cell cortex on the side away from the P1 cell).

### 4.12 Protein Purification and In Vitro Binding Assays

*Escherichia coli* Rosetta (DE3) cells expressing GST‐tagged proteins were harvested and lysed by ultrasonication in pull‐down buffer (50 mm Tris‐HCl, 200 mm NaCl, 1 mm EDTA, 1 mm DTT, pH 8.0) containing protease inhibitors on ice. After centrifugation, the supernatant was incubated with Glutathione Sepharose 4B beads (Cytiva, 17075601) for 2 h at 4°C. After extensive washing, GST‐tagged proteins were eluted with 10 mm reduced glutathione (Amresco, 0399). The Strep‐tagged fusion protein 2×Strep‐HA‐RTN4B was stably expressed in HeLa cells. Harvested cells were lysed in cell lysis buffer (50 mm Tris‐HCl, 150 mm NaCl, 1% Triton X‐100, 1 mm EDTA, pH 7.4) containing protease and phosphatase inhibitors. After centrifugation, the supernatant was incubated with Strep‐Tactin XT 4Flow resins for 2 h at 4°C. After extensive washing, the fusion protein‐bound beads were used for in vitro binding assays.

For in vitro binding assays, beads coated with Strep‐tagged proteins were incubated with purified GST‐tagged proteins for 2 h at 4°C. After extensive washing with lysis buffer, bound proteins were eluted using Strep‐Tactin XT elution buffer (IBA Lifesciences, 2‐1042‐025). Eluted samples were analyzed by Western blotting or Coomassie Brilliant Blue staining.

For GST pull‐down assays, asynchronous and STLC‐arrested mitotic HeLa cells stably expressing 2×Strep‐HA‐RTN4B were lysed in cell lysis buffer containing protease and phosphatase inhibitor cocktails. After centrifugation, the supernatant was incubated with GST‐fusion protein‐bound beads for 2 h at 4°C. The beads were washed extensively with cell lysis buffer and boiled in sample loading buffer. Samples were then analyzed by Western blotting or Coomassie Brilliant Blue staining.

### 4.13 Sample Preparation for Mass Spectrometry Analysis

For mass spectrometry sample preparation, cells were collected and lysed in lysis buffer. After centrifugation, the supernatants were incubated with either protein G‐Sepharose beads (Cytiva, 17061805) prebound with antibodies or anti‐Flag M2 affinity gels (Millipore, A2220) for 2 h at 4°C. After four washes with lysis buffer, the beads were boiled in protein SDS loading buffer. Samples were separated by SDS‐PAGE and stained with Coomassie Brilliant Blue. After tryptic digestion, proteins were analyzed using an Orbitrap Fusion Lumos mass spectrometer (Thermo Fisher Scientific) to identify protein post‐translational modifications.

### 4.14 In vitro Kinase Assay

HeLa cells stably expressing 3×Flag‐mEmerald‐RTN4B WT or 6A mutant were harvested and lysed in lysis buffer (50 mm Tris‐HCl, 150 mm NaCl, 1% Triton X‐100, pH 7.5) containing protease and phosphatase inhibitors. After centrifugation, the supernatant was incubated with anti‐Flag M2 affinity gels for 2 h at 4°C. Flag‐tagged fusion proteins were eluted with 100 µg/mL 3×FLAG peptide. The purified 3×Flag‐mEmerald‐RTN4B WT or 6A mutant was then incubated with active CDK1/Cyclin B1 complex (Abcam, ab271456) in 1× kinase buffer (10 mm Tris‐HCl, 2 mm MgCl_2_, 150 mm NaCl, 1 mm DTT, pH 7.5) containing phosphatase inhibitors and 500 µm ATP (NEB, P0756S) at 30°C for 45 min. The reaction was terminated by adding SDS sample loading buffer, and the samples were analyzed by Western blotting using the appropriate antibodies.

### 4.15 Transmission Electron Microscopy

Wild‐type and RTN4 knockout HeLa cells were plated on ACLAR 33C film (Electron Microscopy Sciences, 50425) and fixed with 2.5% (v/v) glutaraldehyde (Sigma–Aldrich, G5882) in 0.1 m phosphate buffer (PB, pH 7.4). After fixation, the samples were extensively washed with 0.1 m PB and post‐fixed for 30 min at room temperature in the dark using a mixture of 1% osmium tetroxide and 0.8% potassium ferrocyanide (Sigma–Aldrich, 60279). The samples were subsequently incubated in a 1% aqueous solution of uranyl acetate and then washed with double‐distilled water (ddH_2_O). Dehydration was performed through a graded ethanol series (30%, 50%, 70%, 85%, 95%, and 100%; 6 min each), followed by two 6‐min changes in 100% acetone. The cells were gradually infiltrated with Embed 812 resin (Electron Microscopy Sciences, 14120) and polymerized at 65°C for 24 h. After polymerization, the ACLAR 33C film was detached from the polymerized resin blocks before trimming. Ultrathin sections (70 nm thick) were cut using an ultramicrotome (Leica Microsystems, UC7) fitted with a diamond knife (Diatome, Ultra 35°). Serial sections were mounted on single‐slot copper grids and stained with uranyl acetate and lead citrate. Grids were examined using a transmission electron microscope (Thermo Fisher Scientific, Tecnai G2 Spirit BioTWIN) operated at an accelerating voltage of 120 kV. Micrographs were acquired using a digital camera (Gatan, Orius 832).

### 4.16 Focused Ion Beam‐Scanning Electron Microscopy (FIB‐SEM)

HeLa cells stably expressing 3×Flag‐mEmerald‐RTN4B WT were synchronized with thymidine for 20 h, washed three times, and released into fresh medium for 7–8 h. Samples were immediately fixed with 4% (w/v) PFA. Mitotic HeLa cells from prophase to prometaphase were selected based on rounded morphology and RTN4 fluorescence intensity using a Zeiss LSM980 confocal microscope equipped with a 20×/0.8 NA air objective. Cells were subsequently fixed with 2.5% (v/v) glutaraldehyde in 0.1 m PB for 1 h at room temperature. After four washes with 0.1 m PB, samples were post‐fixed with 1% osmium tetroxide and 0.8% potassium ferrocyanide for 1 h at room temperature and washed with ddH_2_O. The samples were then treated with 1% thiocarbohydrazide (Sigma–Aldrich, 223220) for 20 min at room temperature (protected from light), followed by washing with ddH_2_O. Samples were then treated again with 1% osmium tetroxide for 30 min at room temperature. After rinsing again with ddH_2_O, the samples were stained overnight with 1% uranyl acetate at 4°C. Following a final rinse with ddH_2_O, samples were dehydrated through a graded series of ethanol (30%, 50%, 70%, 85%, 95%, and three changes of 100%; 6 min each) and embedded in Embed 812 resin. The cured resin blocks were trimmed and sectioned using an ultramicrotome (Leica Microsystems, UC7) until the selected mitotic cells were tangentially exposed for FIB‐SEM imaging. FIB‐SEM imaging was performed using a Helios Nanolab G3 UC (Thermo Fisher Scientific). Images were acquired at a 10.74 nm pixel size with a 2 kV accelerating voltage and 0.2 nA beam current using the in‐column energy‐selective backscattered electron detector (ICD). A 10 nm FIB step size (*z*‐thickness) was obtained with a 30 kV, 2.5 nA gallium beam.

### 4.17 Segmentation of FIB‐SEM Datasets

Instance segmentation of intracellular structures was performed using the empanada‐napari software package, following a previously described pipeline for applying a generalist deep learning model to instance segmentation in electron microscopy images [56]. First, a subset of the FIB‐SEM images was manually annotated to generate preliminary training labels. These annotated datasets were then used to fine‐tune the generalist deep learning model integrated within the empanada‐napari platform. The model was iteratively refined by adjusting inference parameters and retraining with updated annotations until satisfactory 2D segmentation performance was achieved. This optimized model was subsequently applied to generate 3D segmentations. Three‐dimensional ER segmentations from the pericentrosomal and centrosome‐distal regions of interest were isolated for analysis. Final segmentation accuracy was further refined through manual proofreading, including label merging, splitting, deletion, and filtering steps.

### 4.18 Optogenetic Control of Intracellular Transport Experiments

For optogenetic Rab11 or RTN4 repositioning experiments, HeLa cells stably expressing iLID‐mCherry‐Rab11 or iLID‐mEmerald‐RTN4B and stably expressing doxycycline‐inducible KIF1A (1–365 aa)‐VVDfast‐HA‐SSPB (micro) were treated with 1 µg/ml doxycycline (Selleck, S5159) for 24 h to induce protein expression. Cells were illuminated with blue light (470 nm) for 10 min before fixation and immunofluorescence staining. In optogenetic Rab11 repositioning experiments, endogenous RTN4 localization was detected in blue light‐activated cells using an anti‐RTN4 antibody (Novusbio, NB100‐56681). In optogenetic RTN4 repositioning experiments, mitochondrial and lysosomal localizations were detected using anti‐TOM20 (Proteintech, 11802‐1‐AP) and anti‐LAMP1 (Santa Cruz, sc‐20011) antibodies, respectively.

### 4.19 Microtubule Co‐Sedimentation Assay

To assess the microtubule‐binding ability of RTN4, asynchronous or nocodazole‐arrested mitotic HeLa cells were lysed in PIPES buffer (80 mm PIPES, 1 mm MgCl_2_, 1 mm EGTA, 100 mm NaCl, 1% Triton X‐100, pH 6.8) supplemented with a protease inhibitor cocktail for 30 min on ice. After two rounds of centrifugation at 20 000 *g* for 20 min at 4°C, the supernatant was supplemented with 1 mm GTP and 40 µm Taxol (Selleck, S1150) and incubated at either 4°C or 37°C for 30 min to induce tubulin polymerization. Samples were subsequently centrifuged at 20 000 g for 30 min at 4°C or 37°C. Pellet (P) and supernatant (S) fractions were collected and analyzed by immunoblotting.

### 4.20 Cell Proliferation Assay

A total of 1000 cells were seeded per well in a 96‐well plate. After cell attachment, proliferation was monitored every 24 h using the Super‐Enhanced Cell Counting Kit‐8 (CCK‐8, Beyotime, C0048M) according to the manufacturer's instructions. Absorbance was measured at 450 nm using a microplate reader (BioTek Instruments, Cytation 5 Cell Imaging Multimode Reader).

### 4.21 Developmental Stage and Worm Body Size Measurement

To assess the effect of *ret‐1* on development, approximately 12 gravid adult worms were transferred to fresh OP50‐1 plates to lay eggs for 4 h. Adults were then removed, and the plates were incubated at 20°C. After 49 h, the developmental stages of all progeny were scored using a stereomicroscope (Motic, SMZ168‐B). For each biological replicate, at least 120 animals per condition were scored. For body size measurements, six gravid adult worms were transferred to fresh OP50‐1 plates to lay eggs. After 4 h, adults were removed, and the plates were incubated at 20°C for 65 h. Twenty worms per condition were then immobilized for 5 min in 4 mg/mL tetramisole/M9 solution and aligned. Images were captured using a stereomicroscope (Motic, SMZ‐171), and individual body lengths were measured with ImageJ. All data presented in the figures represent composites of at least three biological replicates. Biological replicates were conducted on separate days using different populations of animals.

### 4.22 Statistical Analysis

Statistical analyses were performed using GraphPad Prism 8. Data were presented as mean ± standard error of the mean (s.e.m.) from at least three independent experiments unless otherwise specified. Statistical significance between groups was determined using a two‐tailed unpaired Student's *t*‐test, Mann–Whitney *U*‐test, or one‐way analysis of variance (ANOVA) with Dunnett's or Tukey's multiple comparisons test. *P* values are shown in the figures. *P* < 0.05 was considered significant.

## Author Contributions

X.X. designed and conducted the molecular biology experiments, cell biology experiments and performed data analysis; Y.L. conducted experiments on *C. elegans* and performed data analysis; R.W. and W.X. conducted some molecular biology experiments and performed data analysis; H.S. conducted EM reconstruction; N.H. contributed to analysis of the results; J.T., P.Z., J.M., and J.C. are the senior authors who designed the project; X.X., J.T., P.Z., and J.C. wrote the manuscript.

## Funding

This work was supported by the National Natural Science Foundation of China (32130024, 32270740, 32470735, 32370732, 32570806, and 32400971), the Beijing Natural Science Foundation (5244029), the Scientific Research Common Program of Beijing Municipal Commission of Education (KM202410025031), and the Chinese Institutes for Medical Research, Beijing (CX23YQB13).

## Conflicts of Interest

The authors declare no conflicts of interest.

## Supporting information




**Supporting File 1**: advs76193‐sup‐0001‐SuppMat.docx.

**Supporting File 2**: advs76193‐sup‐0002‐VideoS1‐S3.zip.

**Supporting File 3**: advs76193‐sup‐0003‐DataFile.pdf.

## Data Availability

The data that support the findings of this study are available from the corresponding author upon reasonable request.
