# Supplementary material for: Pericentrosomal Redistribution of the Endoplasmic Reticulum Ensures Organelle Symmetric Inheritance and Mitotic Progression
Source: Adv Sci (Weinh). 2026 Jun 22;13(51):e76193. doi: 10.1002/advs.76193 (PMC13337110; doi:10.1002/advs.76193)
Supplement: Supplementary file 1 [file ADVS-13-e76193-s001.pdf]

Supporting Information

Pericentrosomal Redistribution of the Endoplasmic Reticulum Ensures Organelle Symmetric Inheritance and Mitotic Progression

Xiangyu Xu, Yalin Liu, Rongyi Wang, Wenwen Xu, Hao Shi, Ning Huang, Junlin Teng, Jin Meng*, Pengli Zheng*, Jianguo Chen**

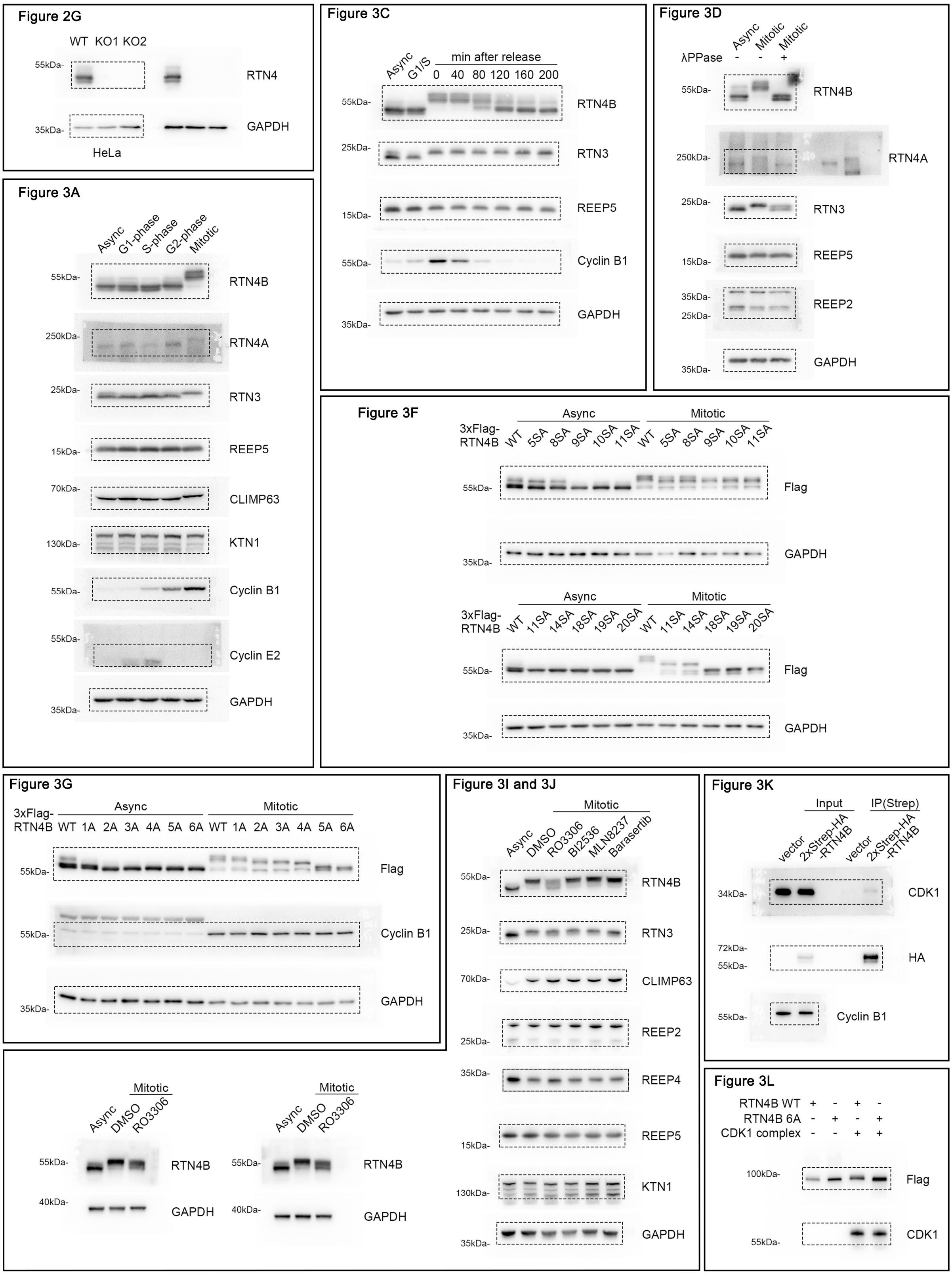

Raw data 1. Raw data of Western blots. Related to Figures 2–3.

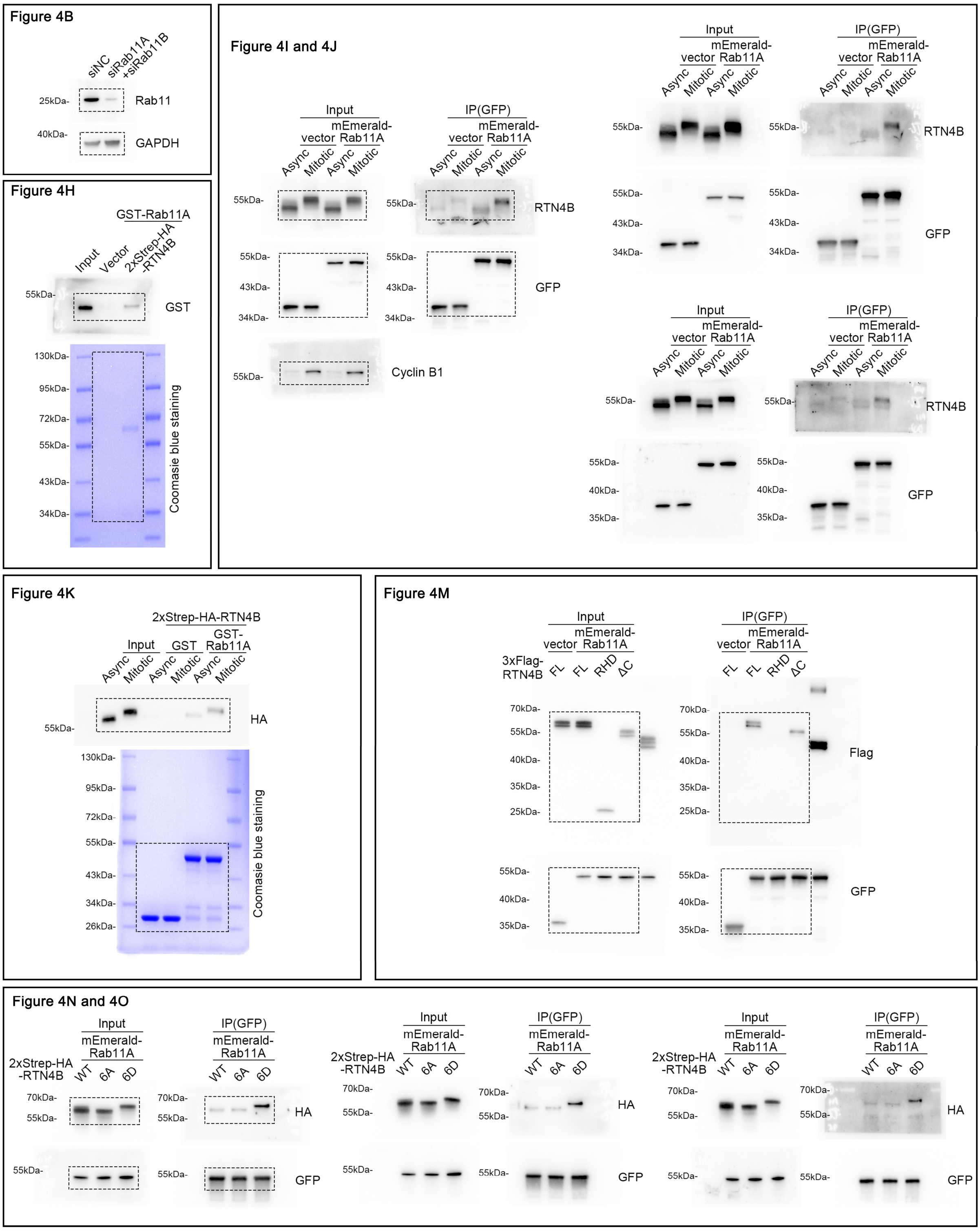

Raw data 2. Raw data of Western blots. Related to Figure 4.

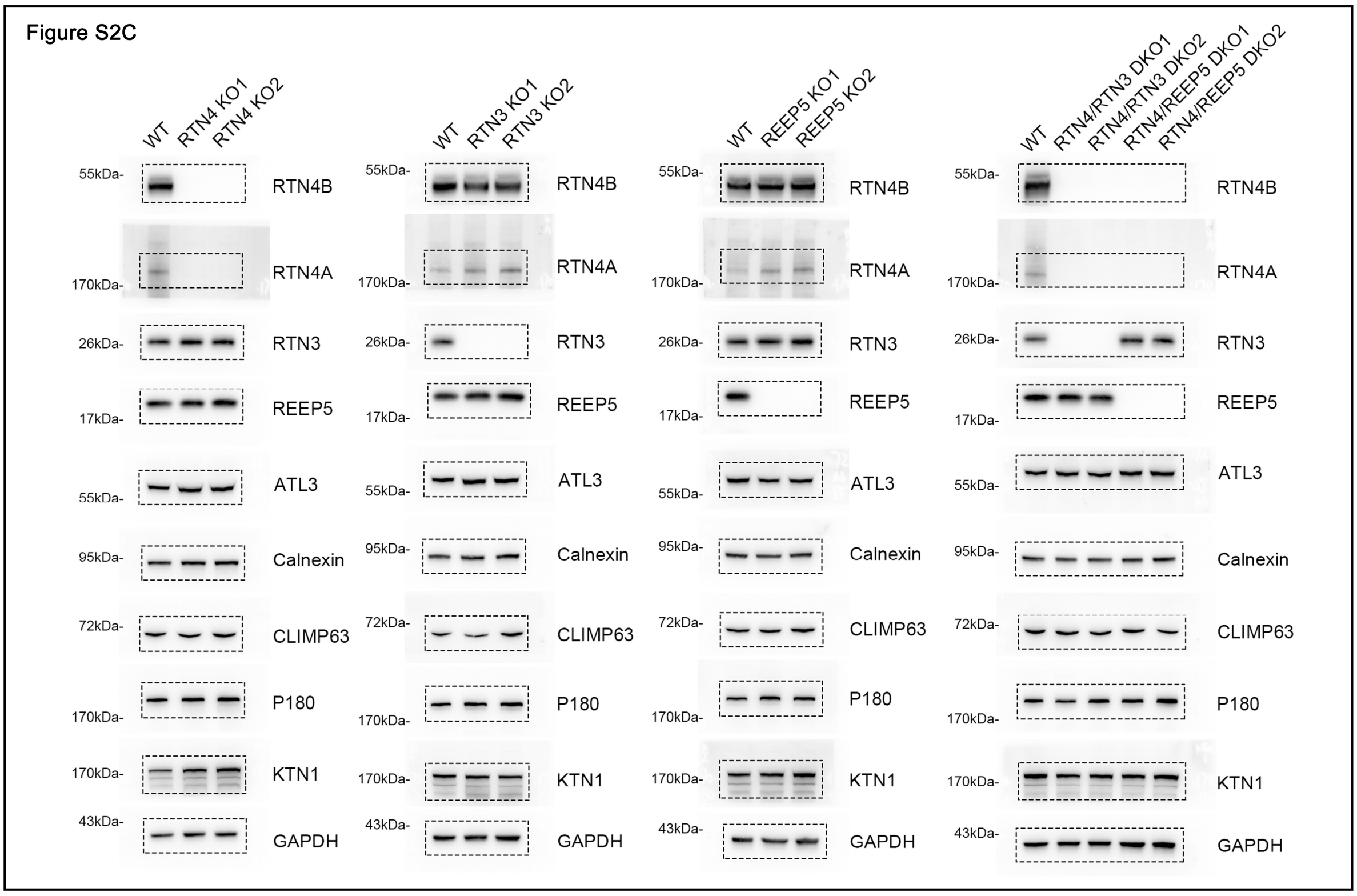

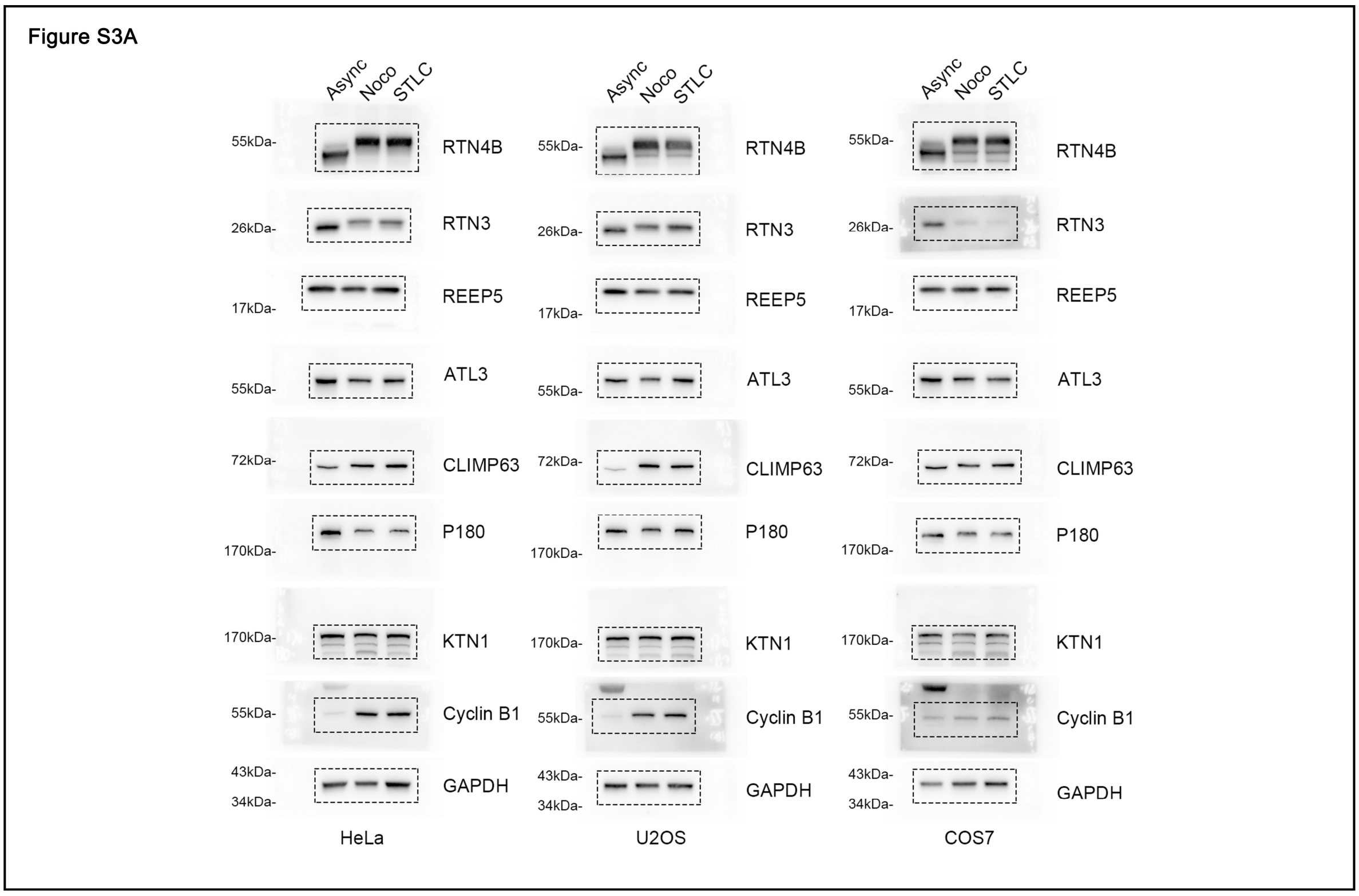

Raw data 3. Raw data of Western blots. Related to Figures S2–S3.

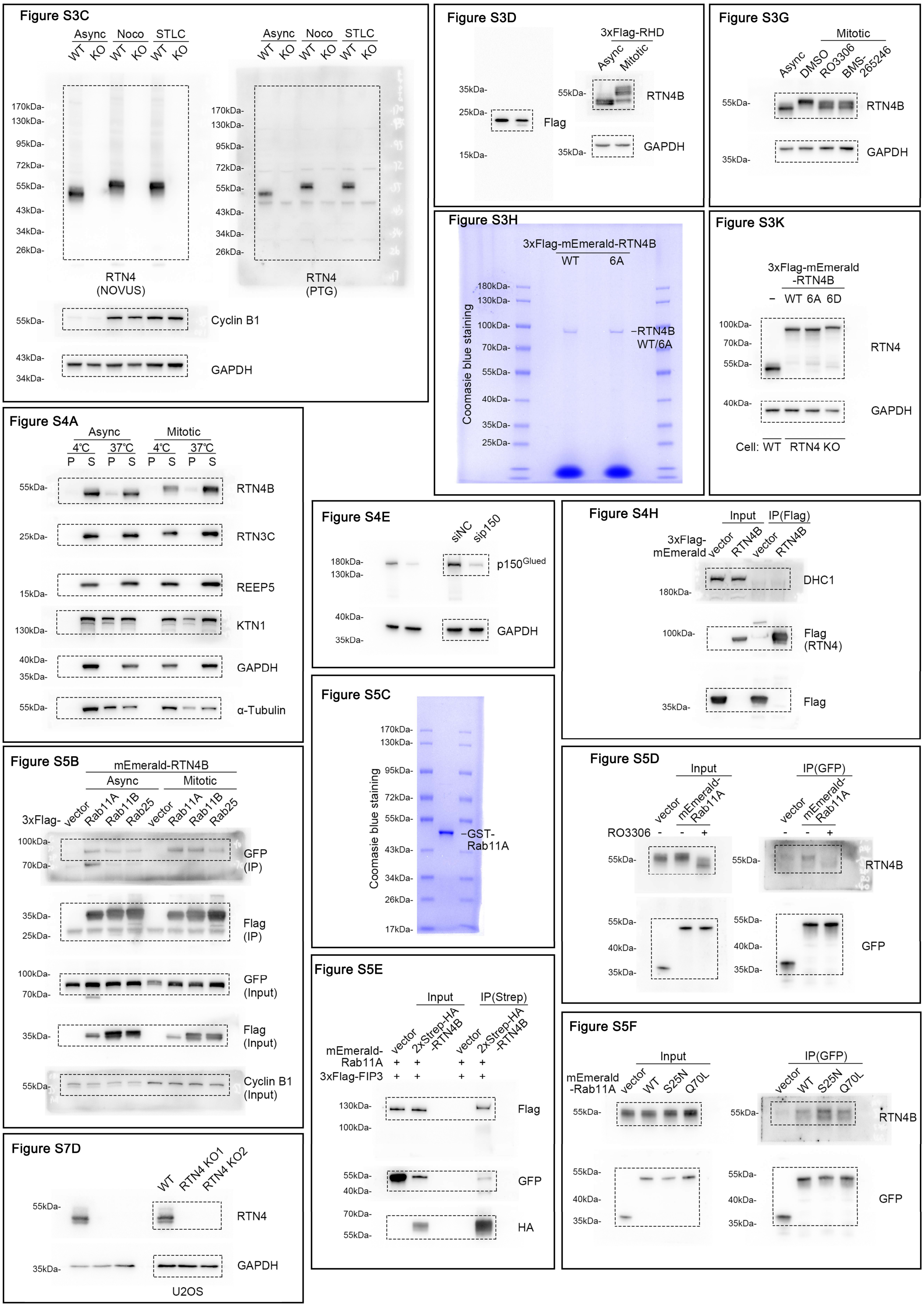

Raw data 4. Raw data of Western blots. Related to Figures S3–S5, Figure S7.

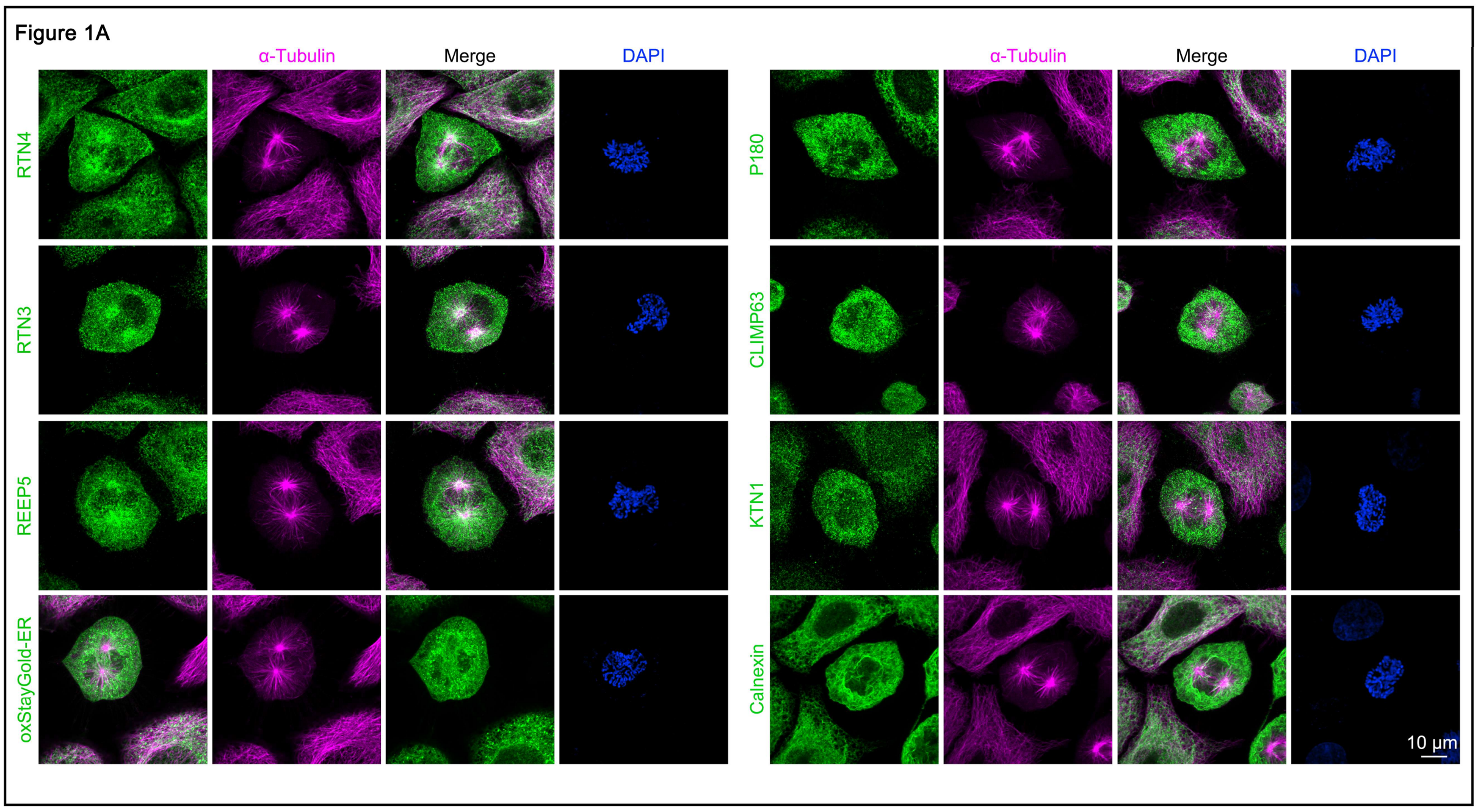

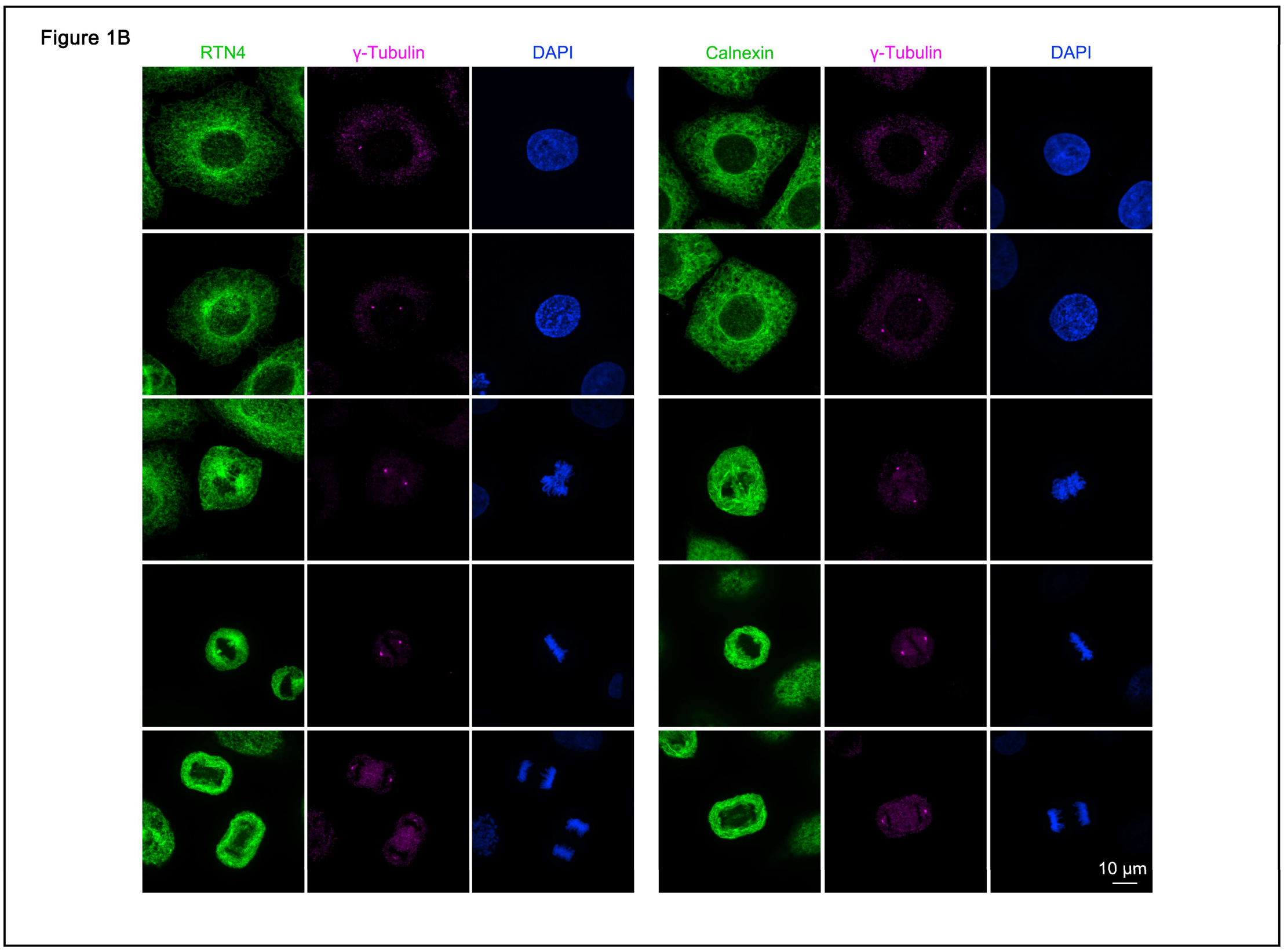

Raw data 5. Raw data of microscope images. Related to Figure 1.

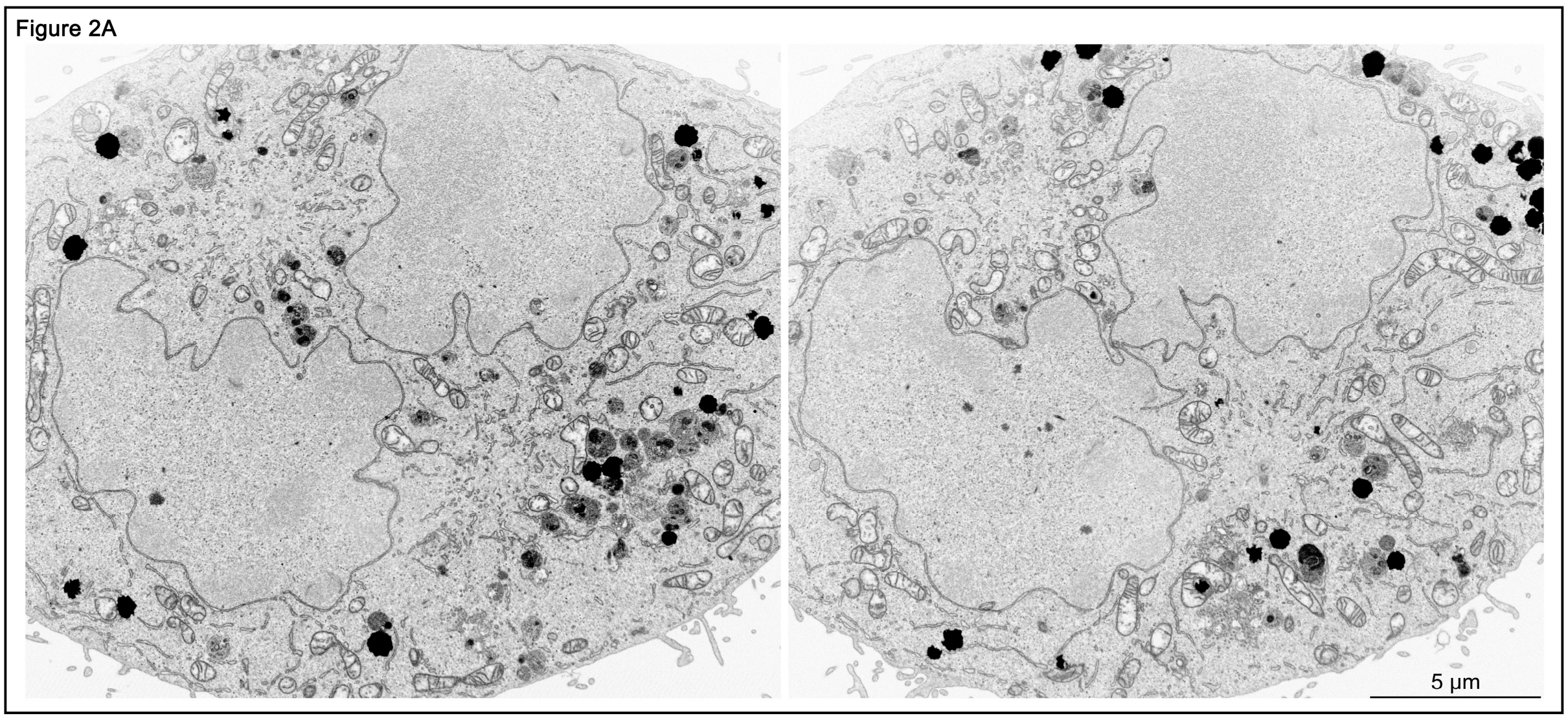

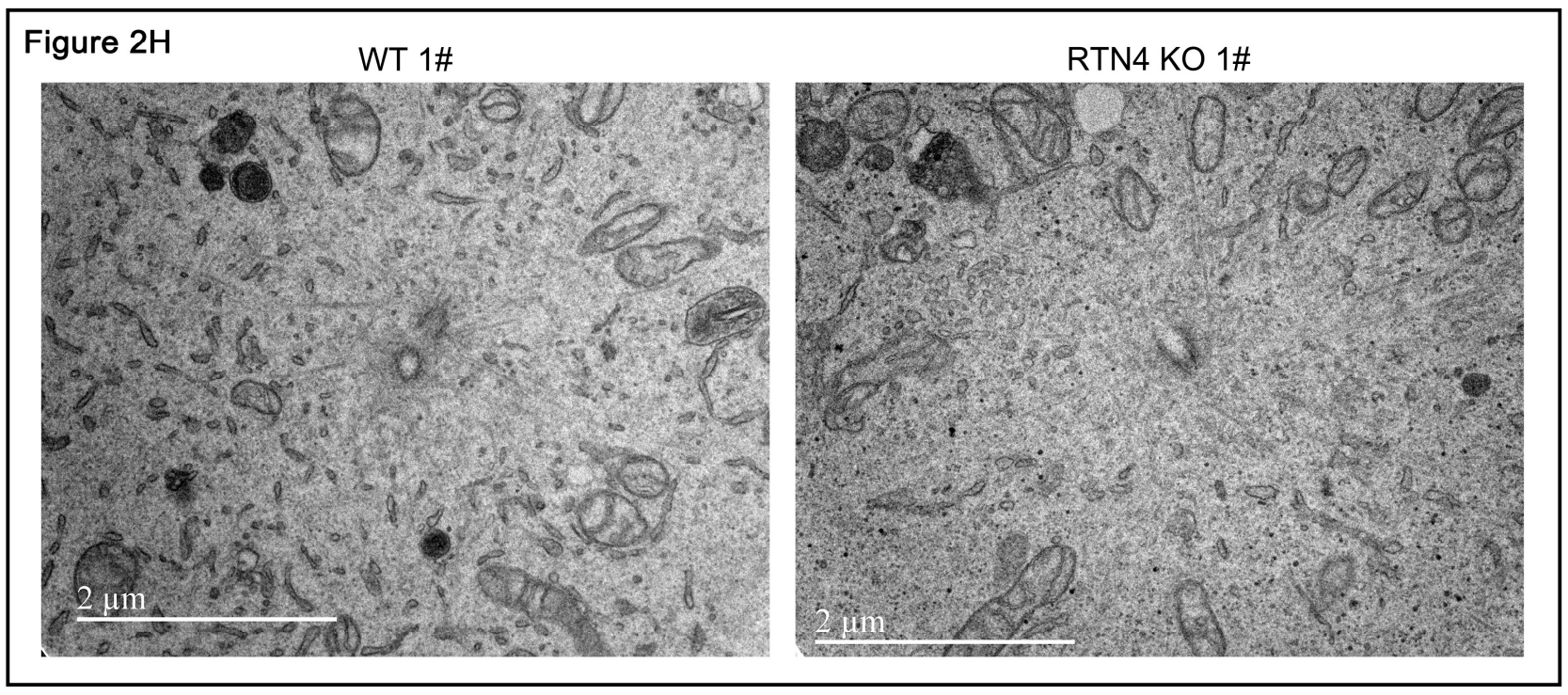

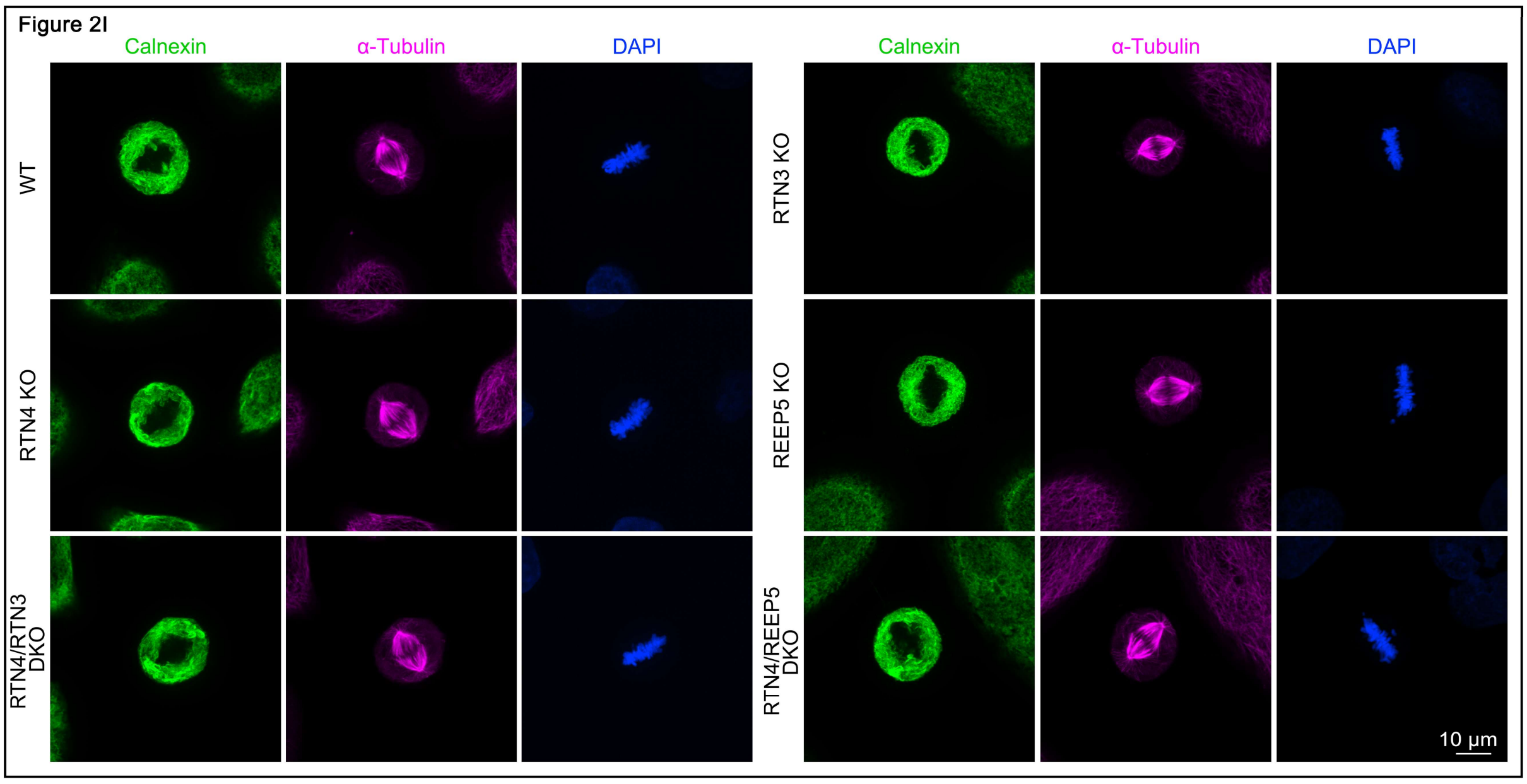

Raw data 6. Raw data of microscope images. Related to Figure 2.

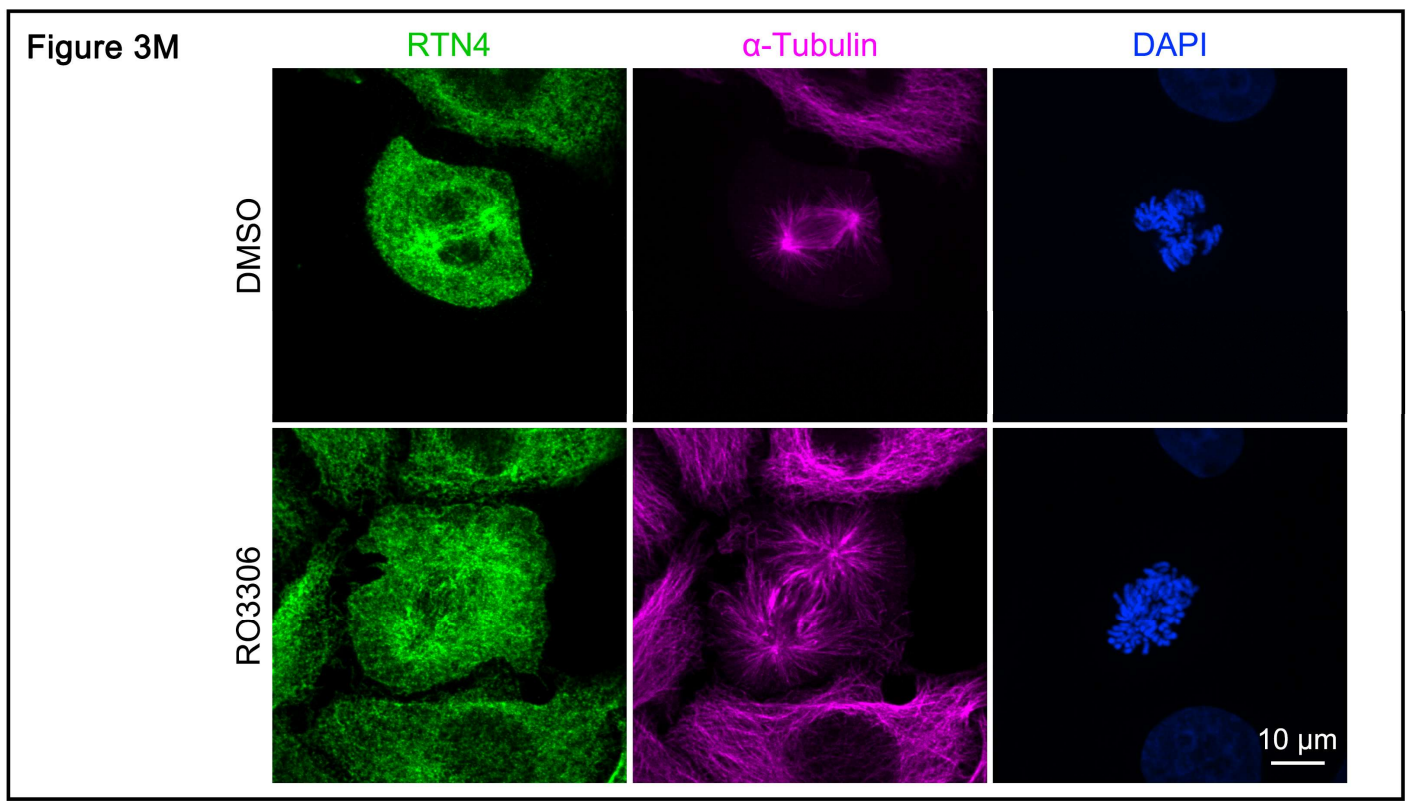

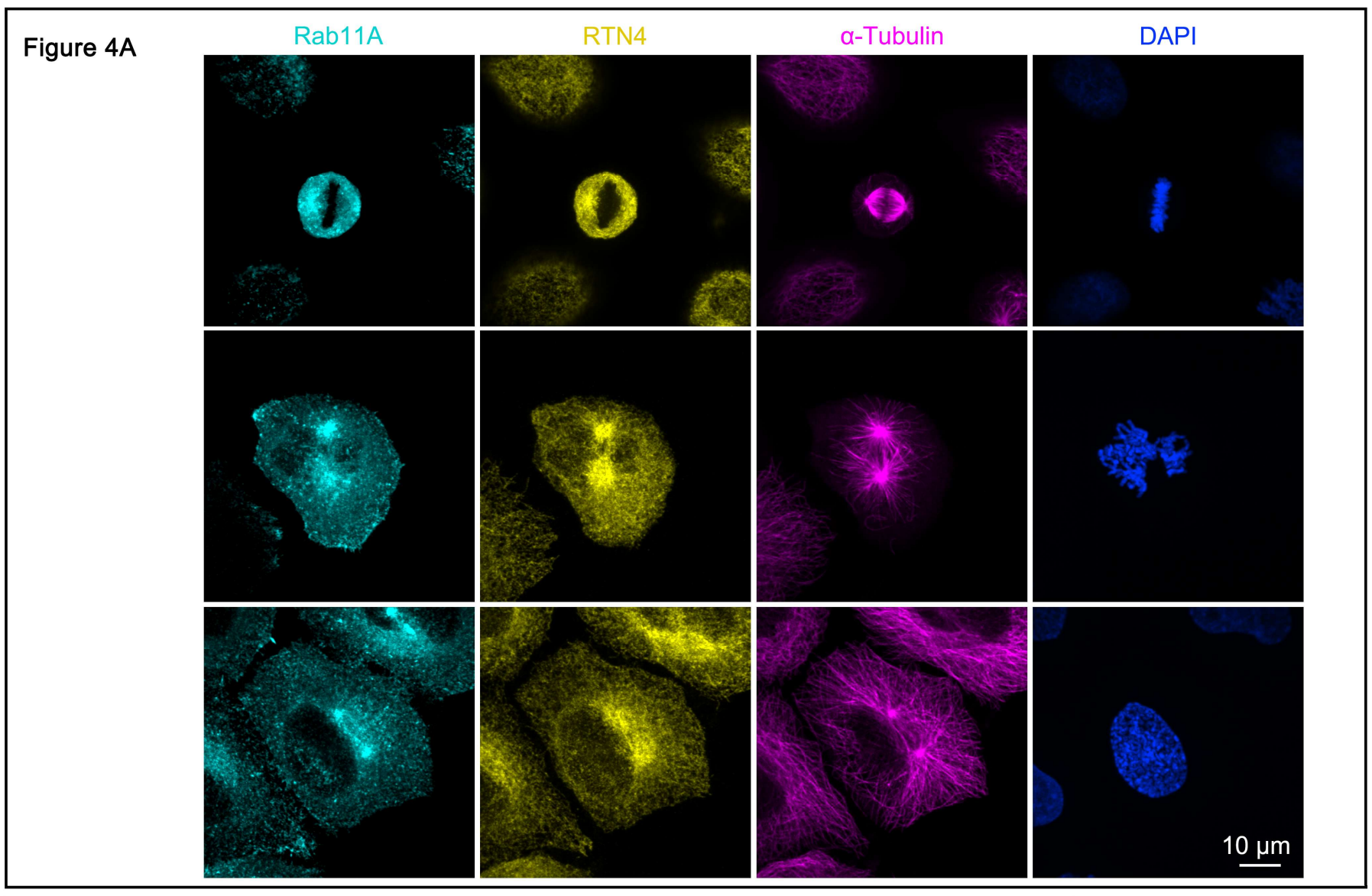

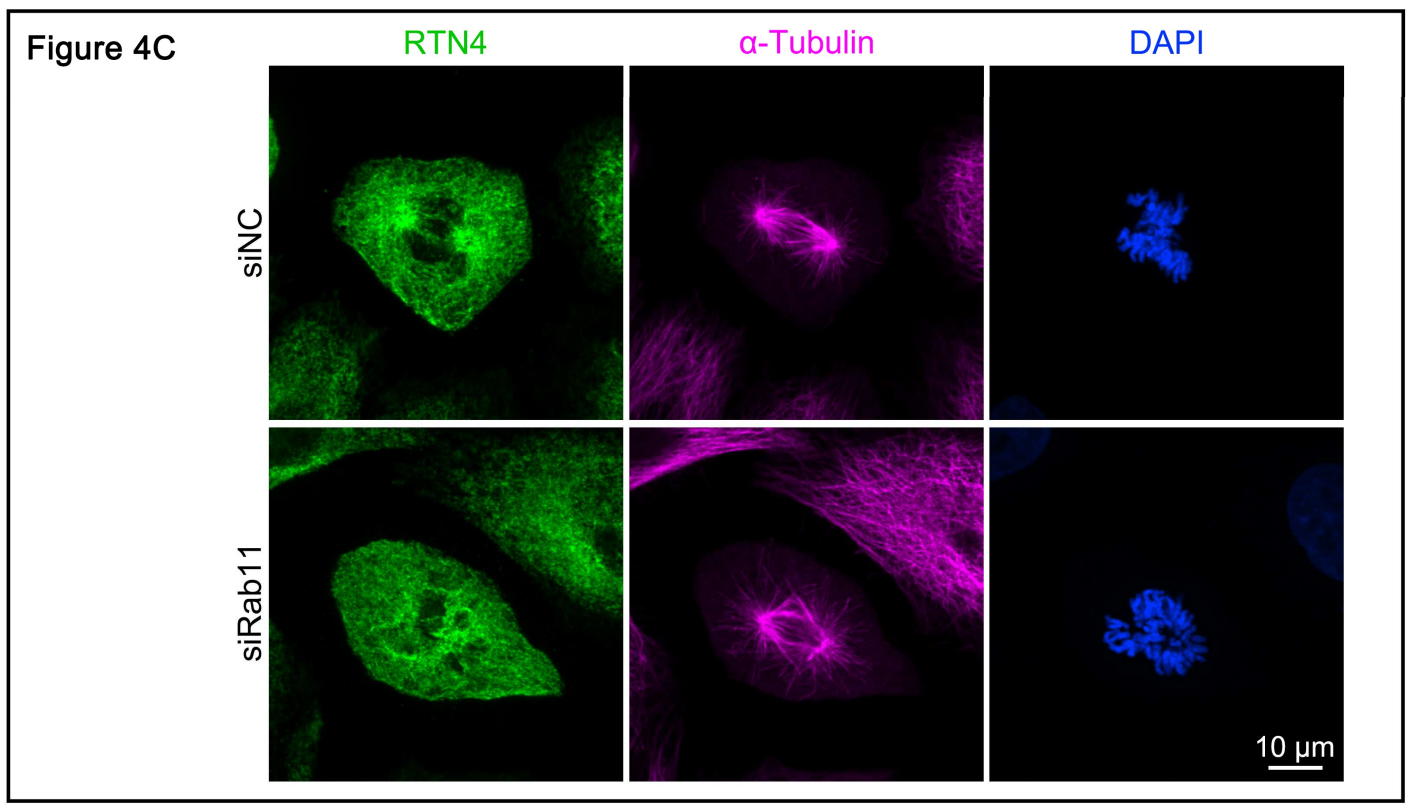

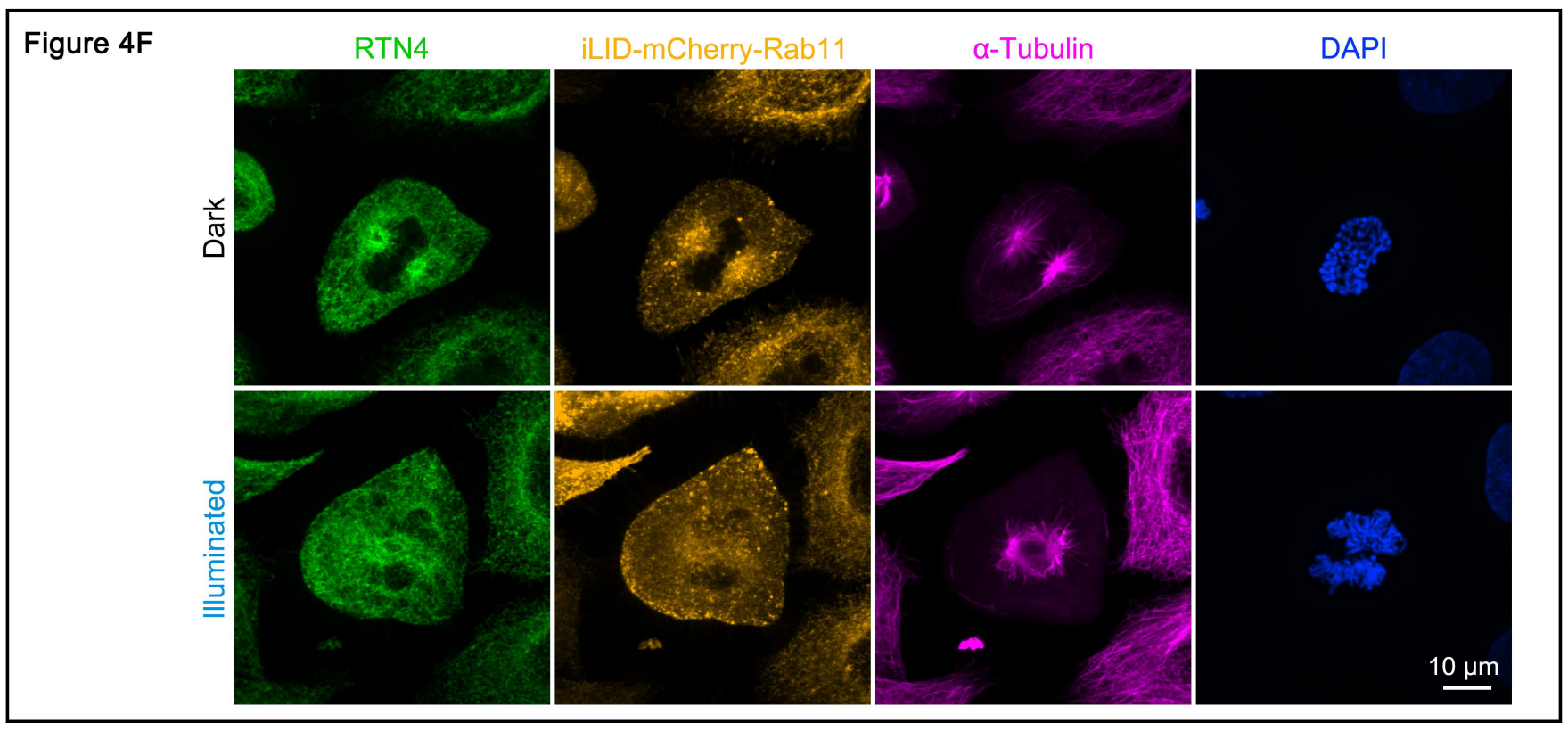

Raw data 7. Raw data of microscope images. Related to Figures 3–4.

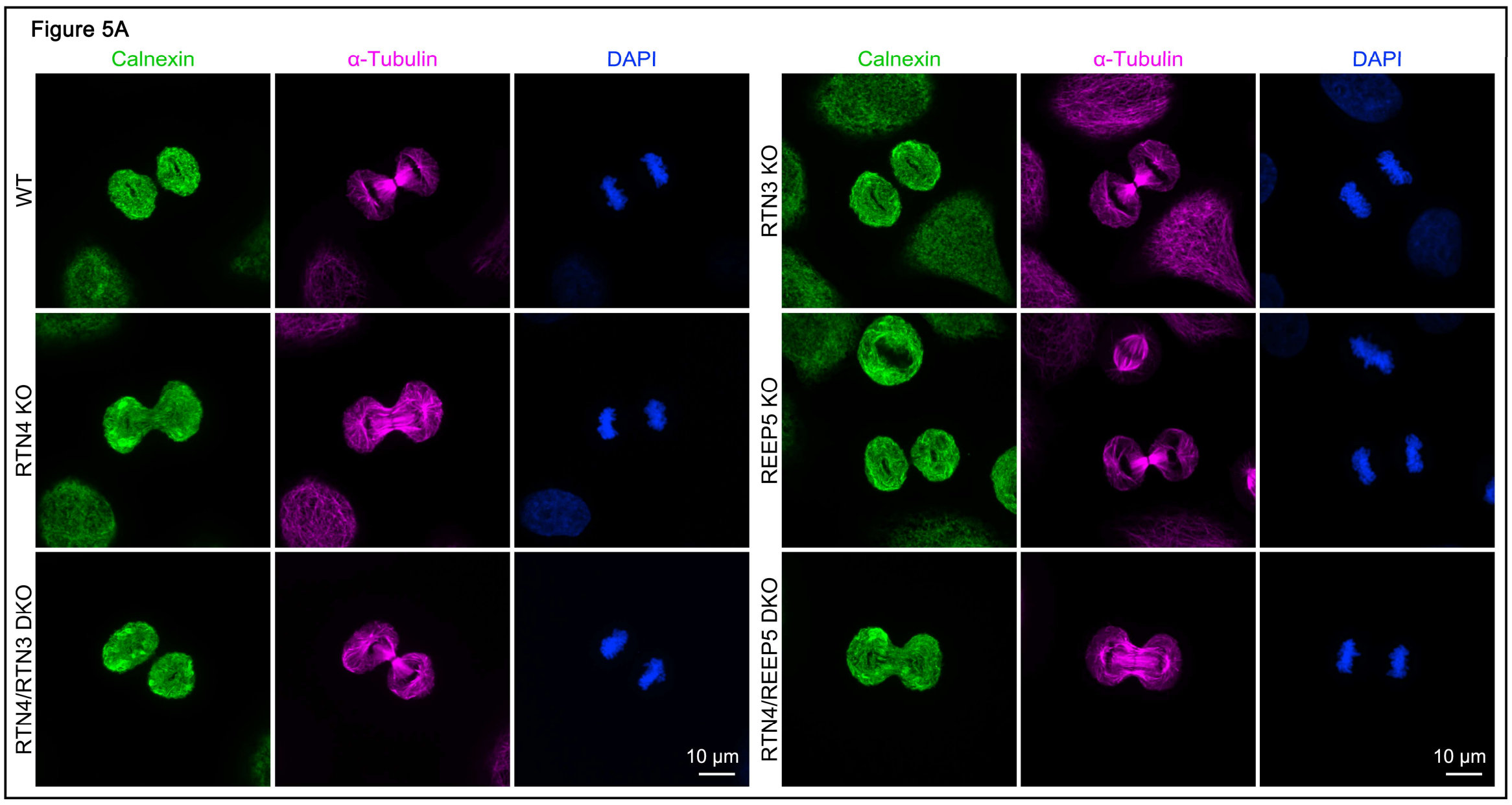

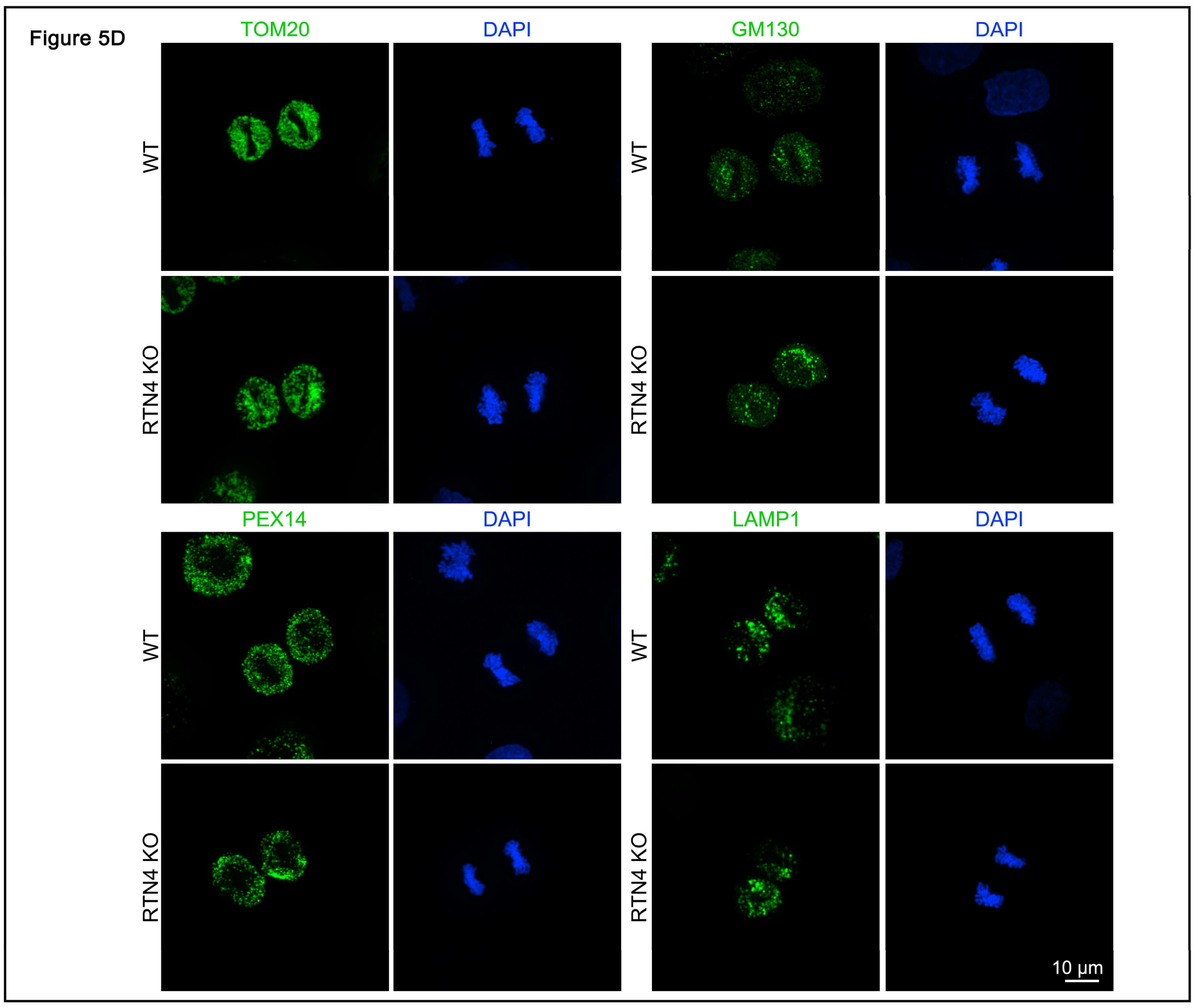

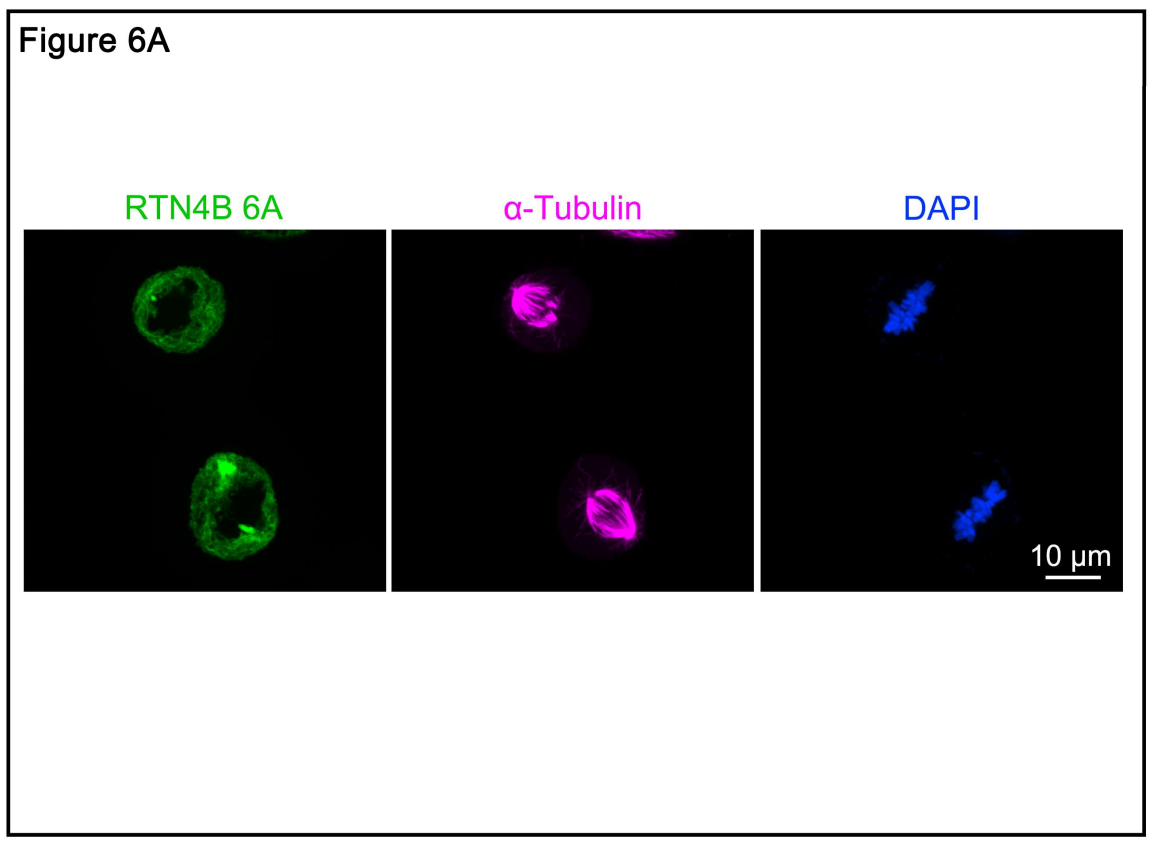

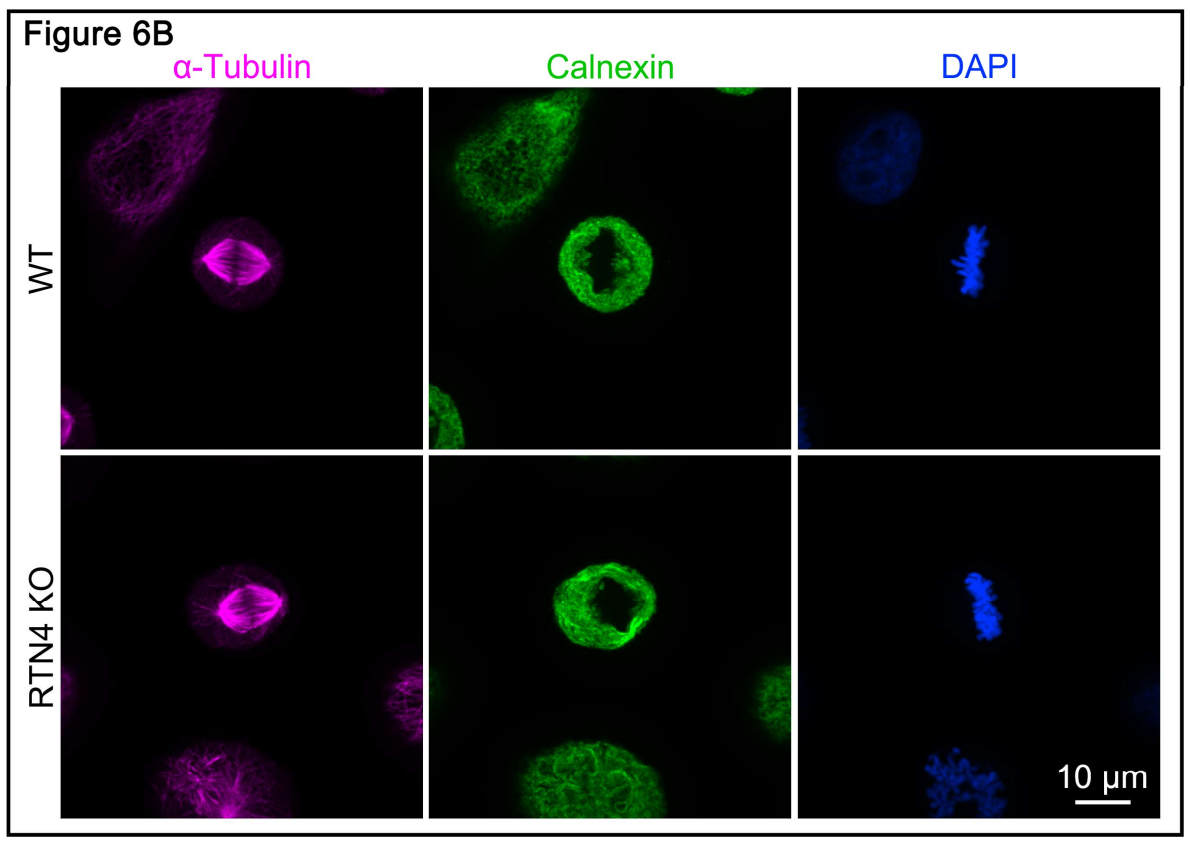

Raw data 8. Raw data of microscope images. Related to Figures 5–6.

Figure S1A

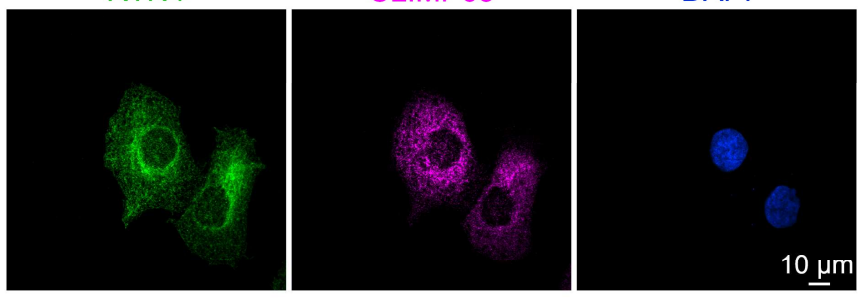

Figure S1B

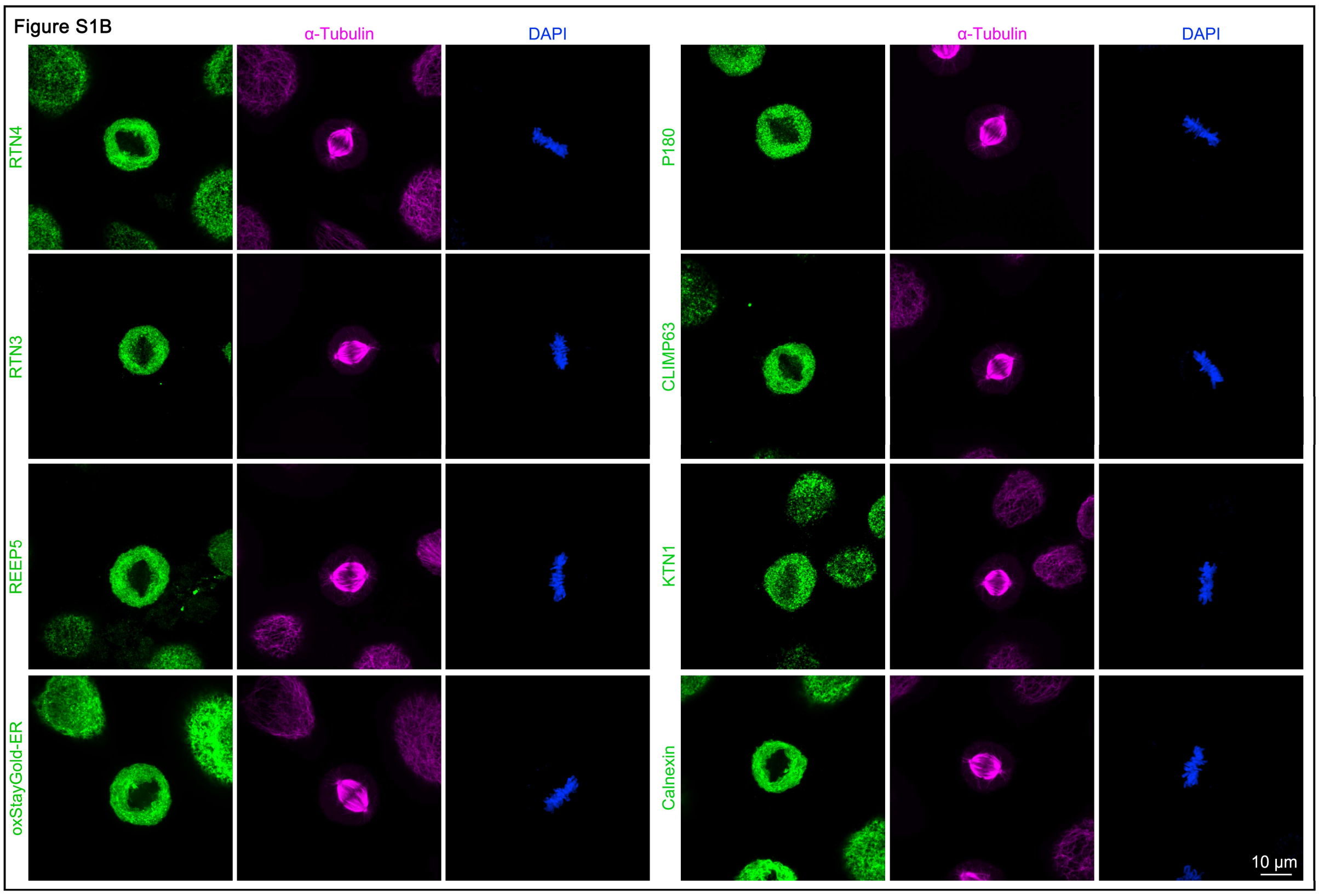

Raw data 9. Raw data of microscope images. Related to Figure S1.

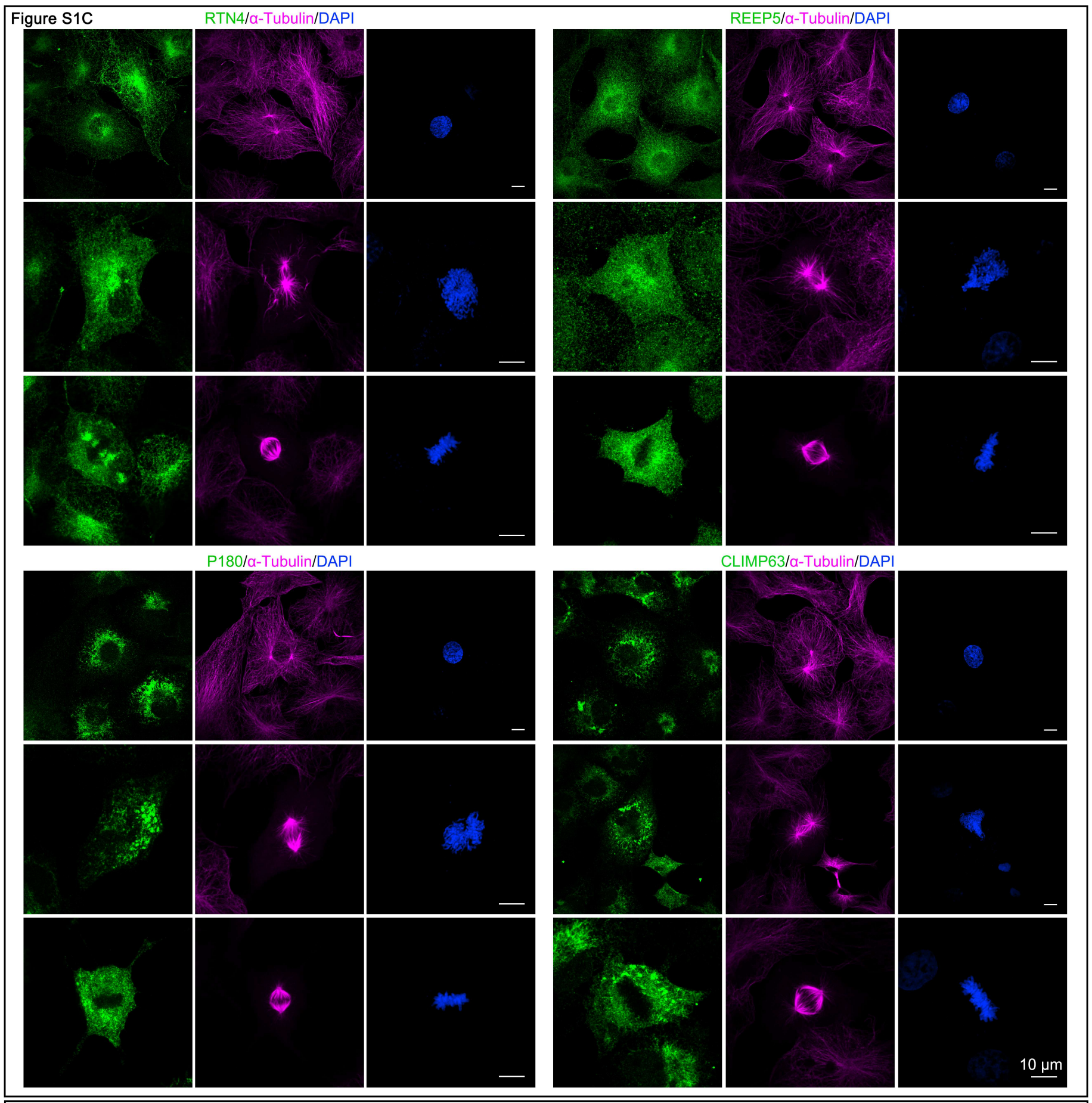

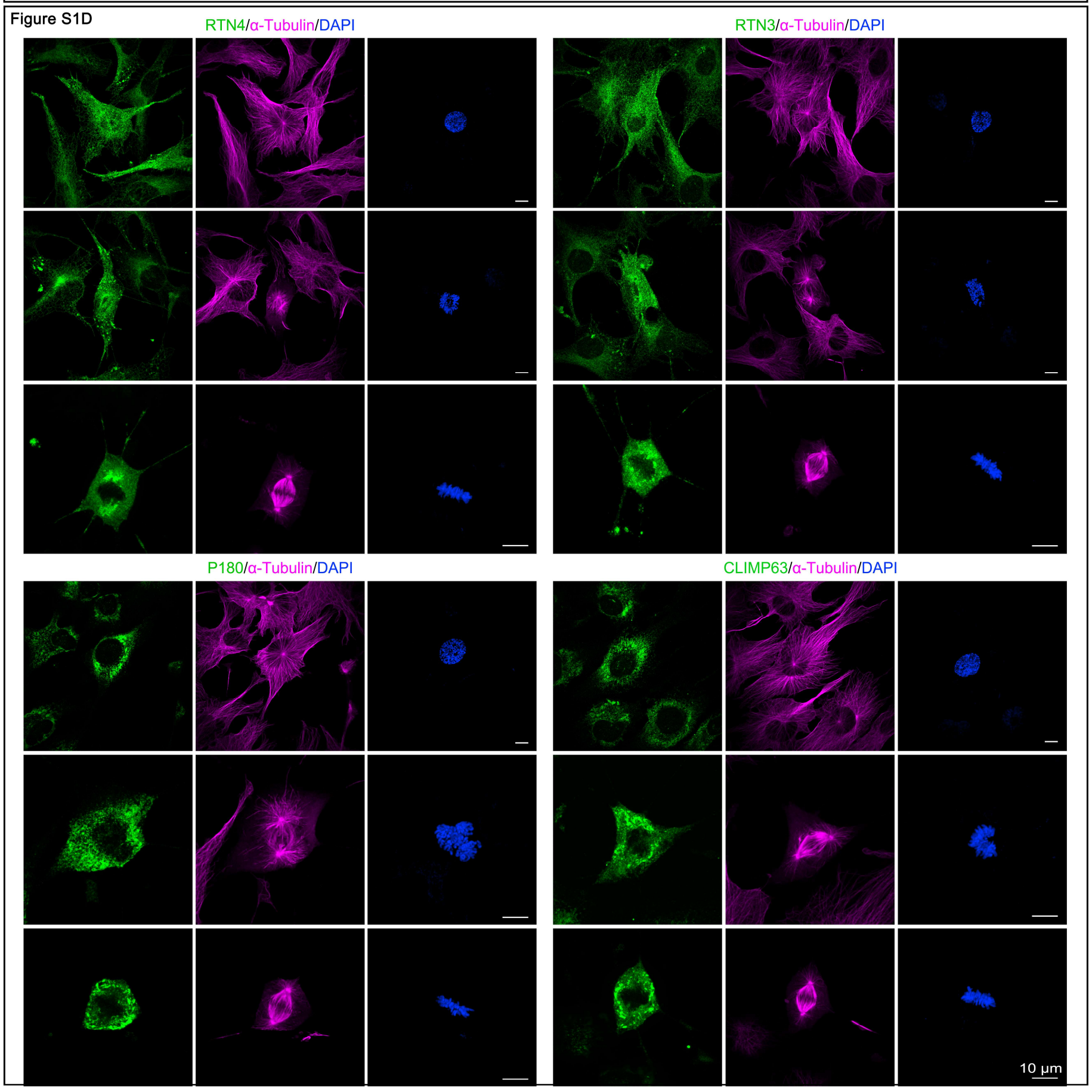

Raw data 10. Raw data of microscope images. Related to Figure S1.

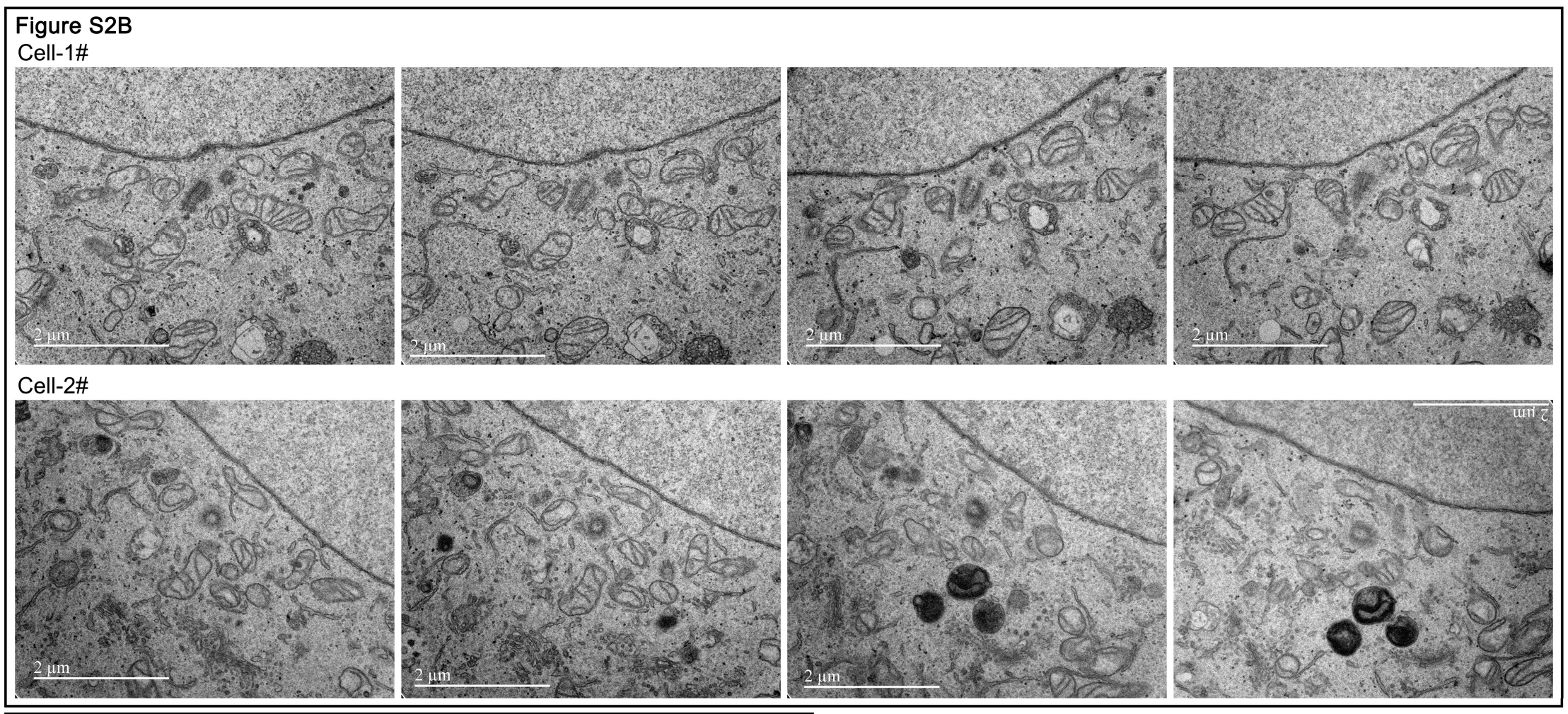

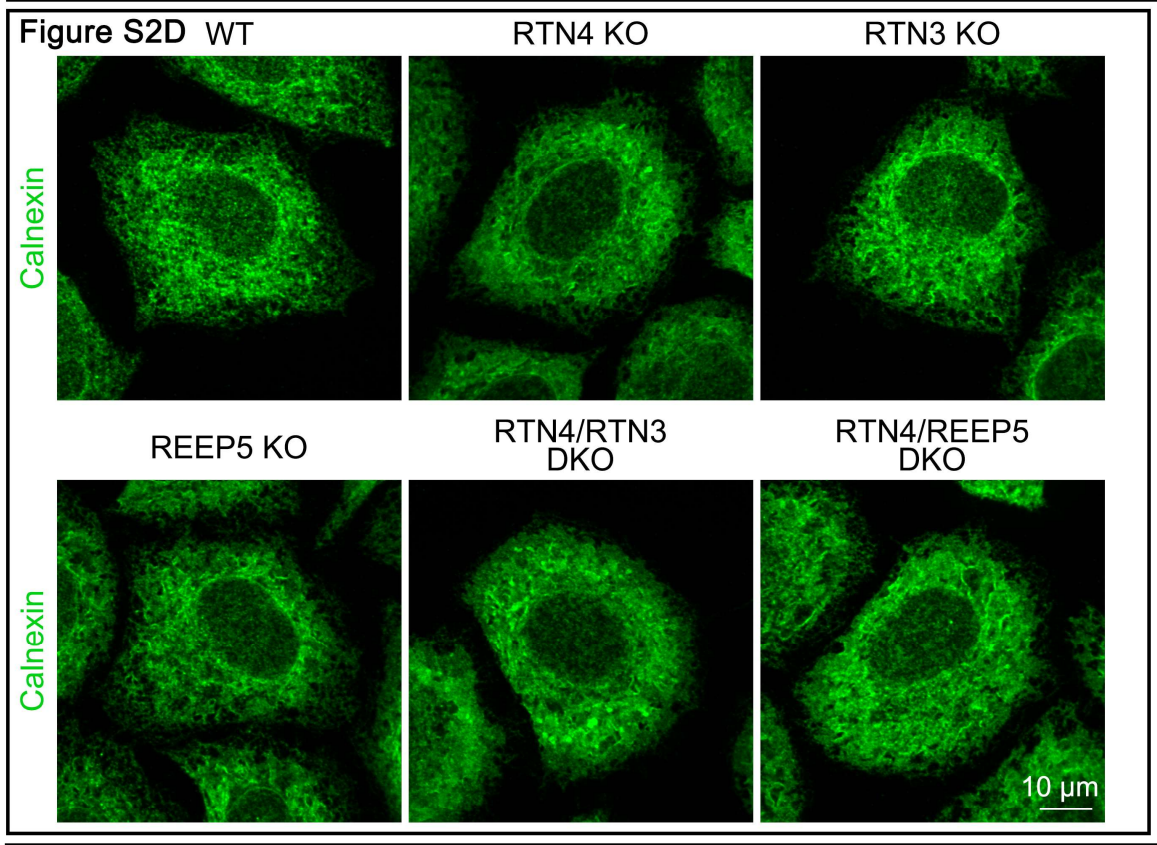

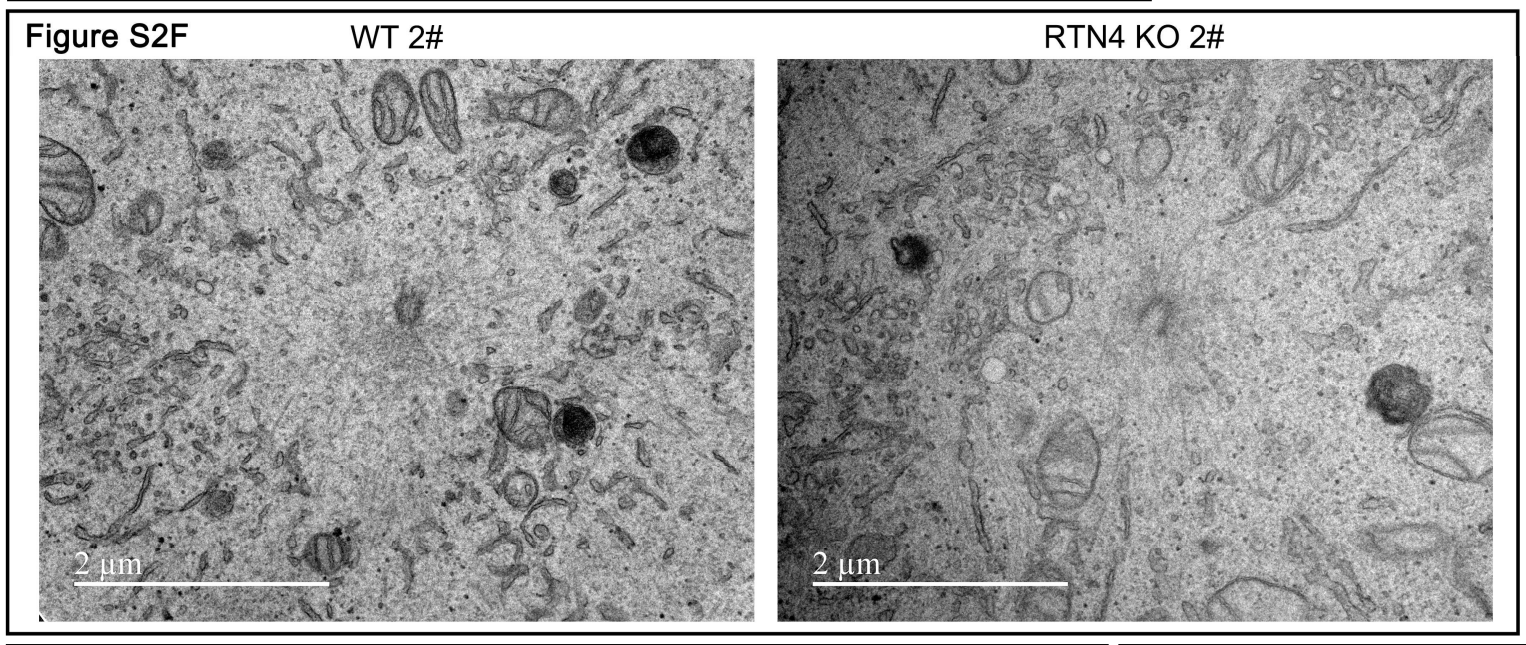

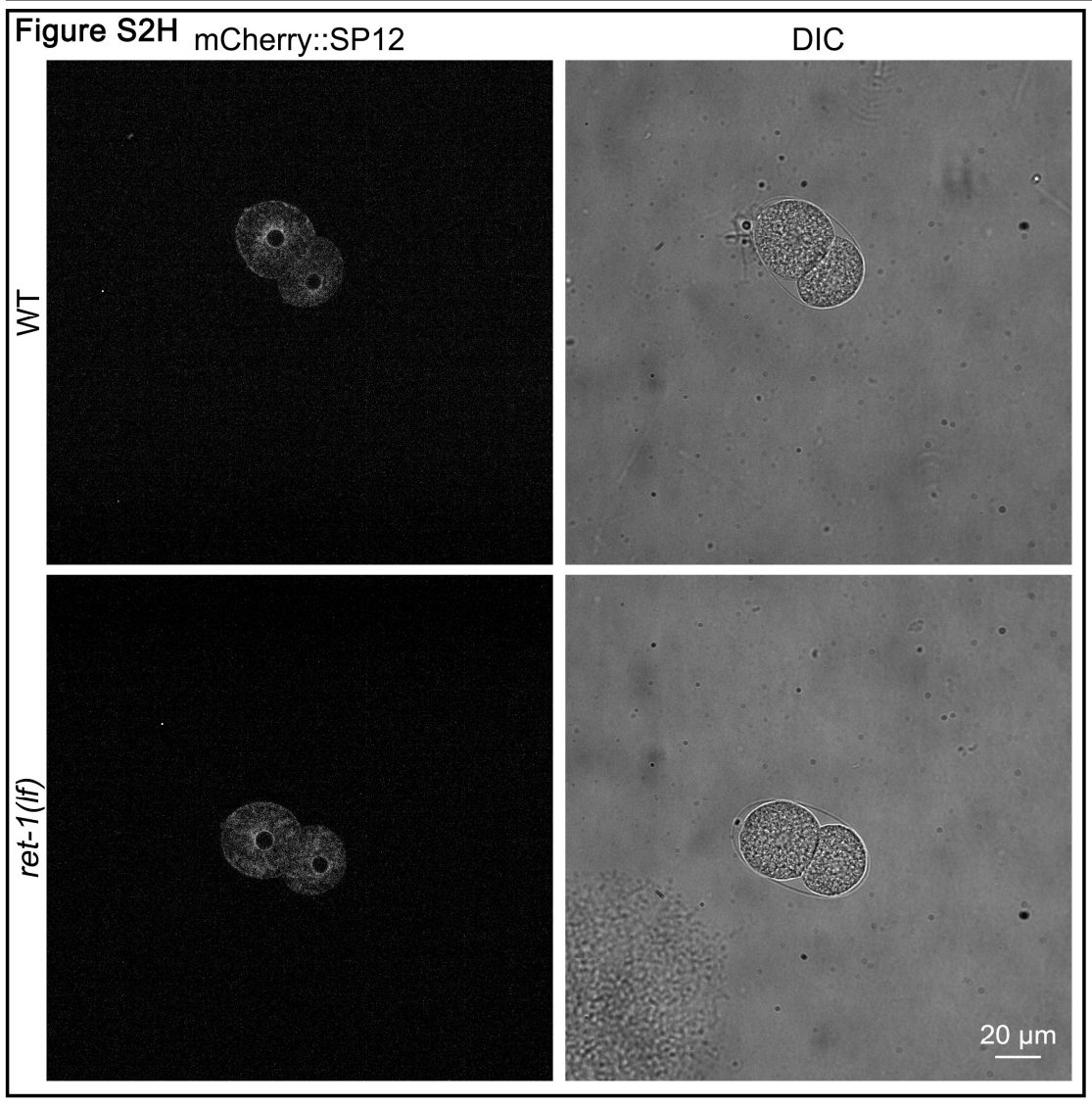

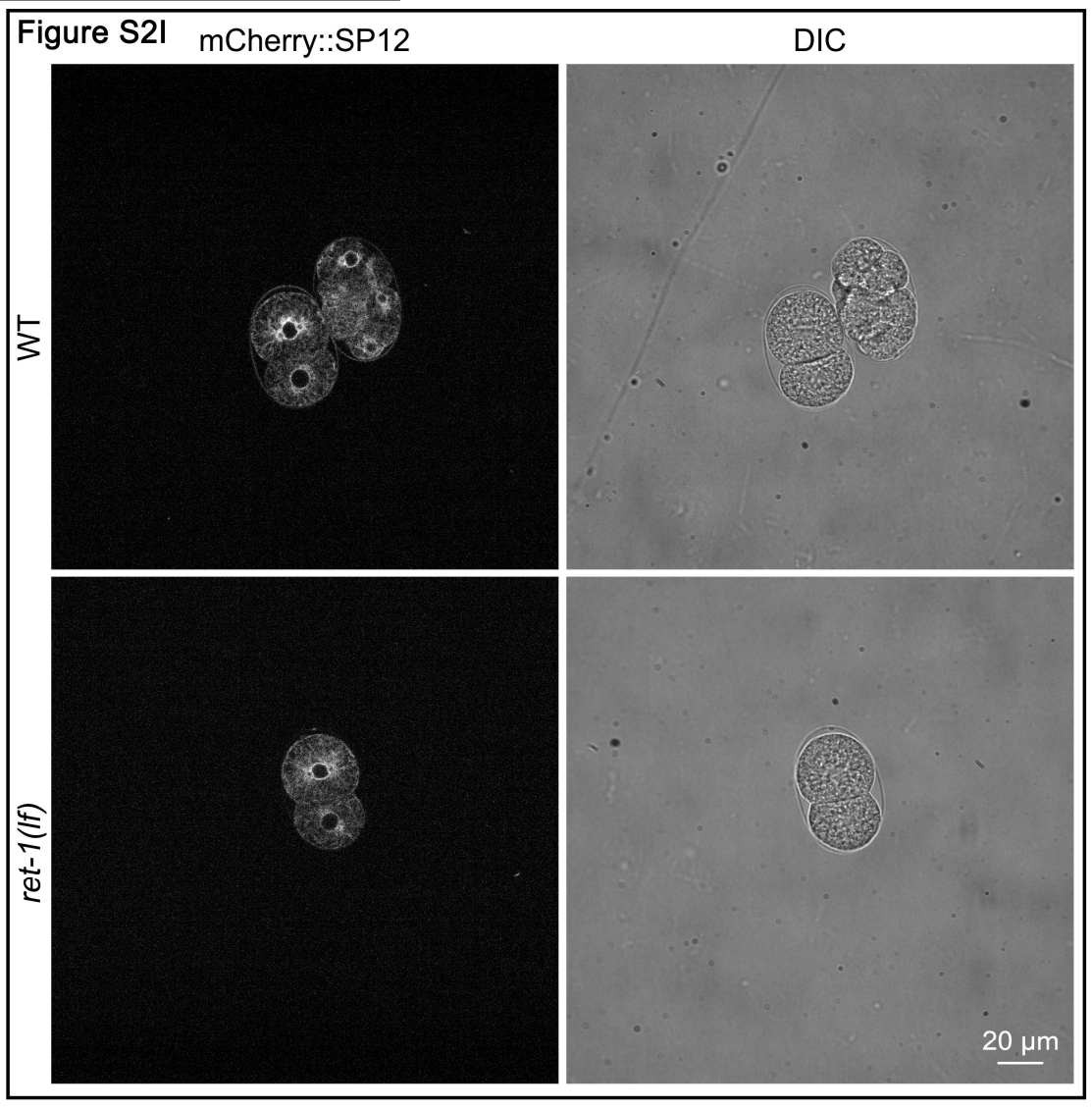

Raw data 11. Raw data of microscope images. Related to Figure S2.

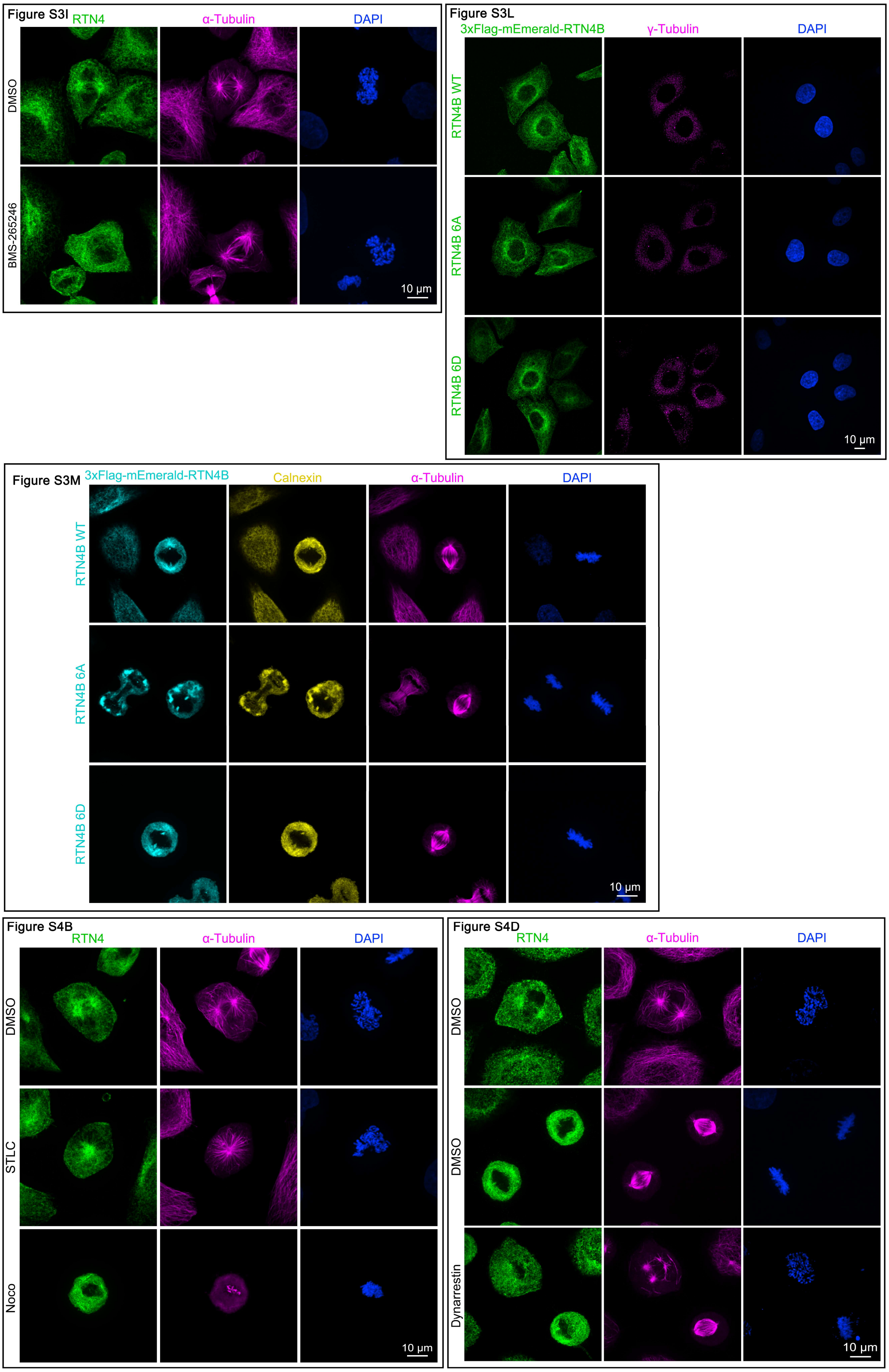

Raw data 12. Raw data of microscope images. Related to Figures S3–S4.

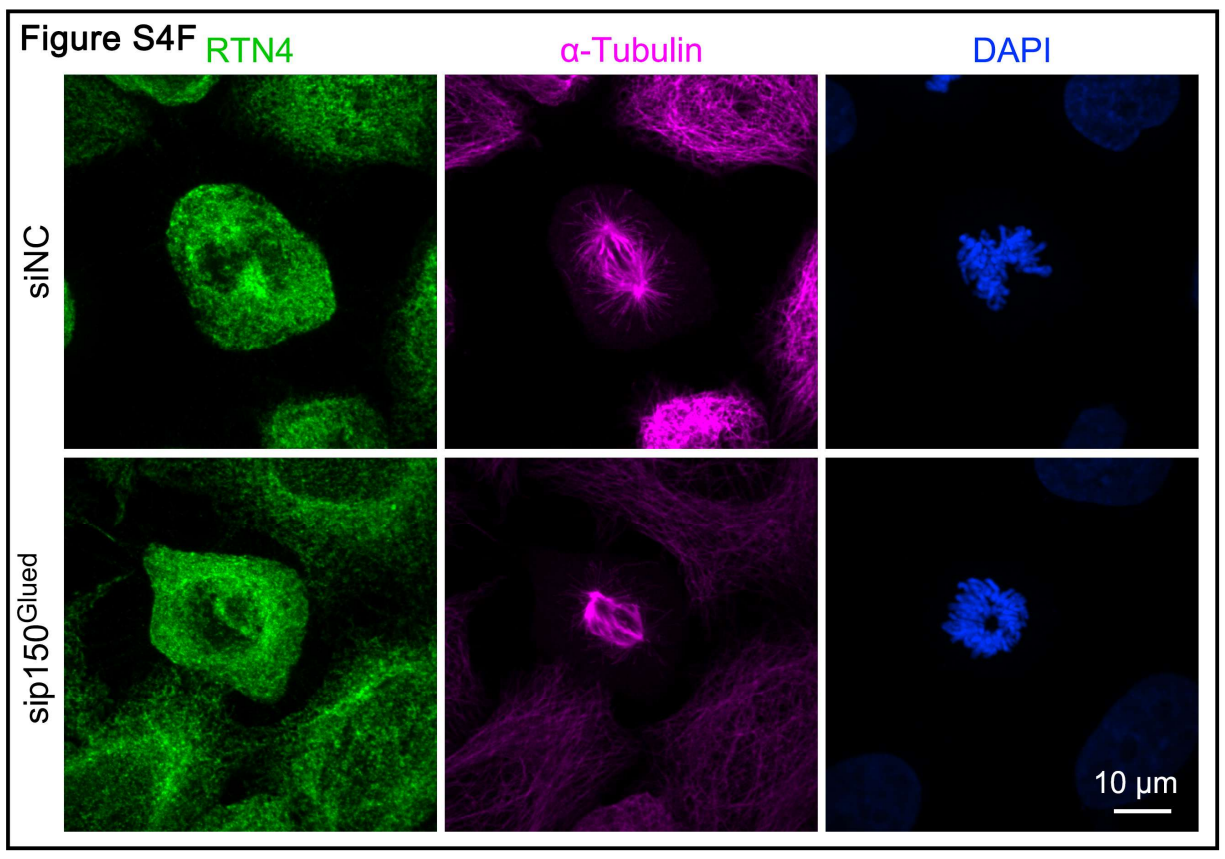

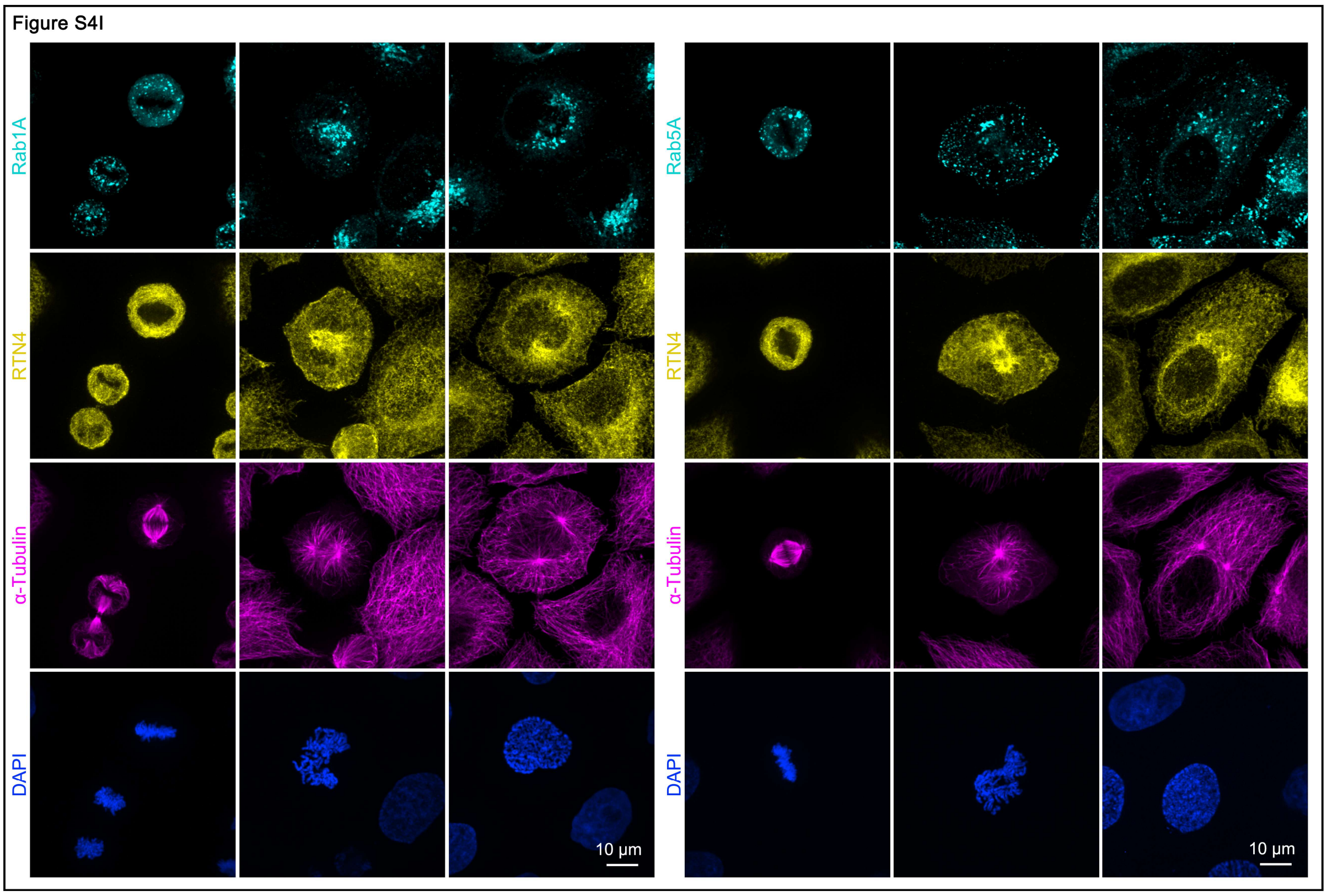

Raw data 13. Raw data of microscope images. Related to Figure S4.

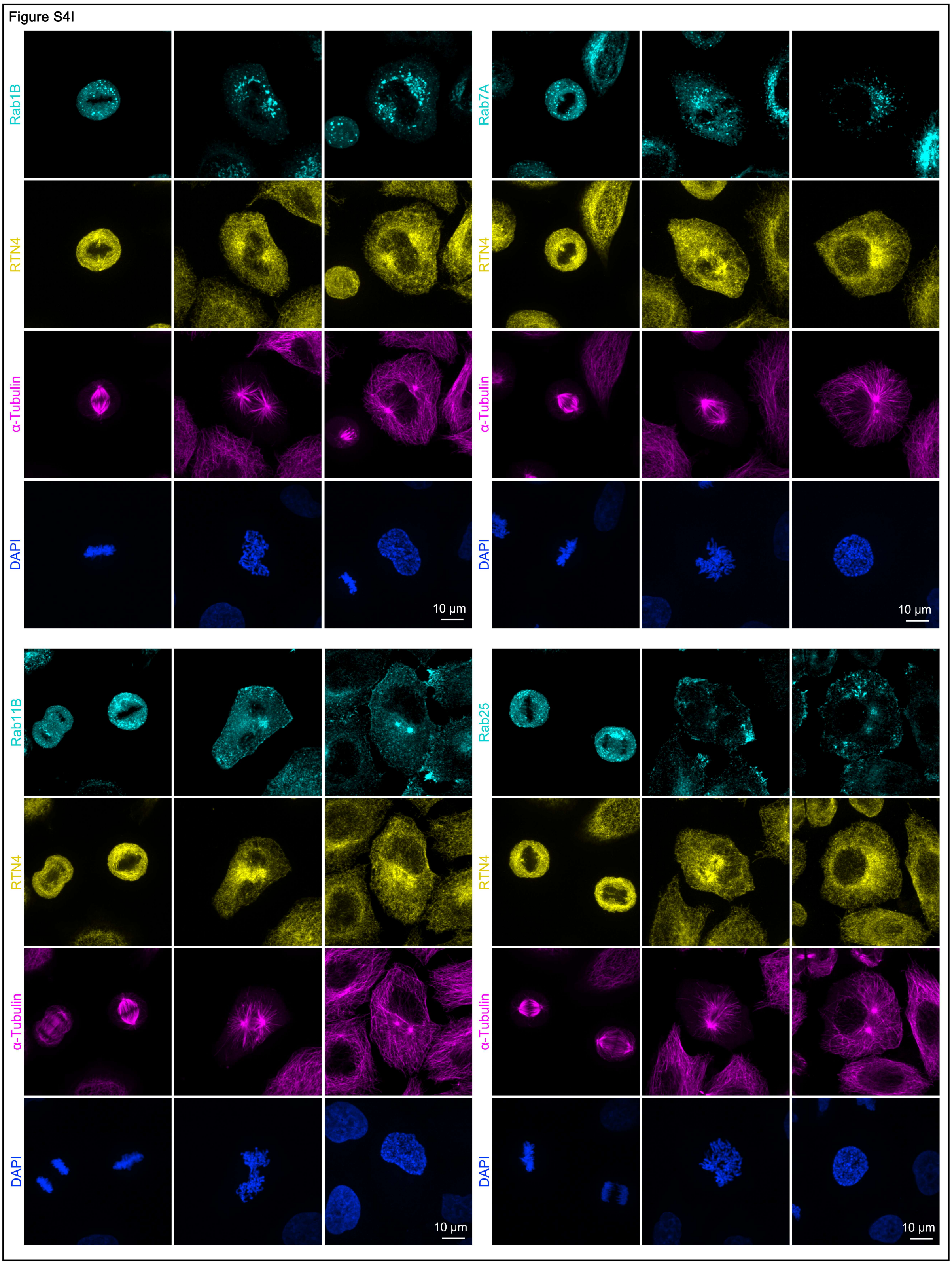

Raw data 14. Raw data of microscope images. Related to Figure S4.

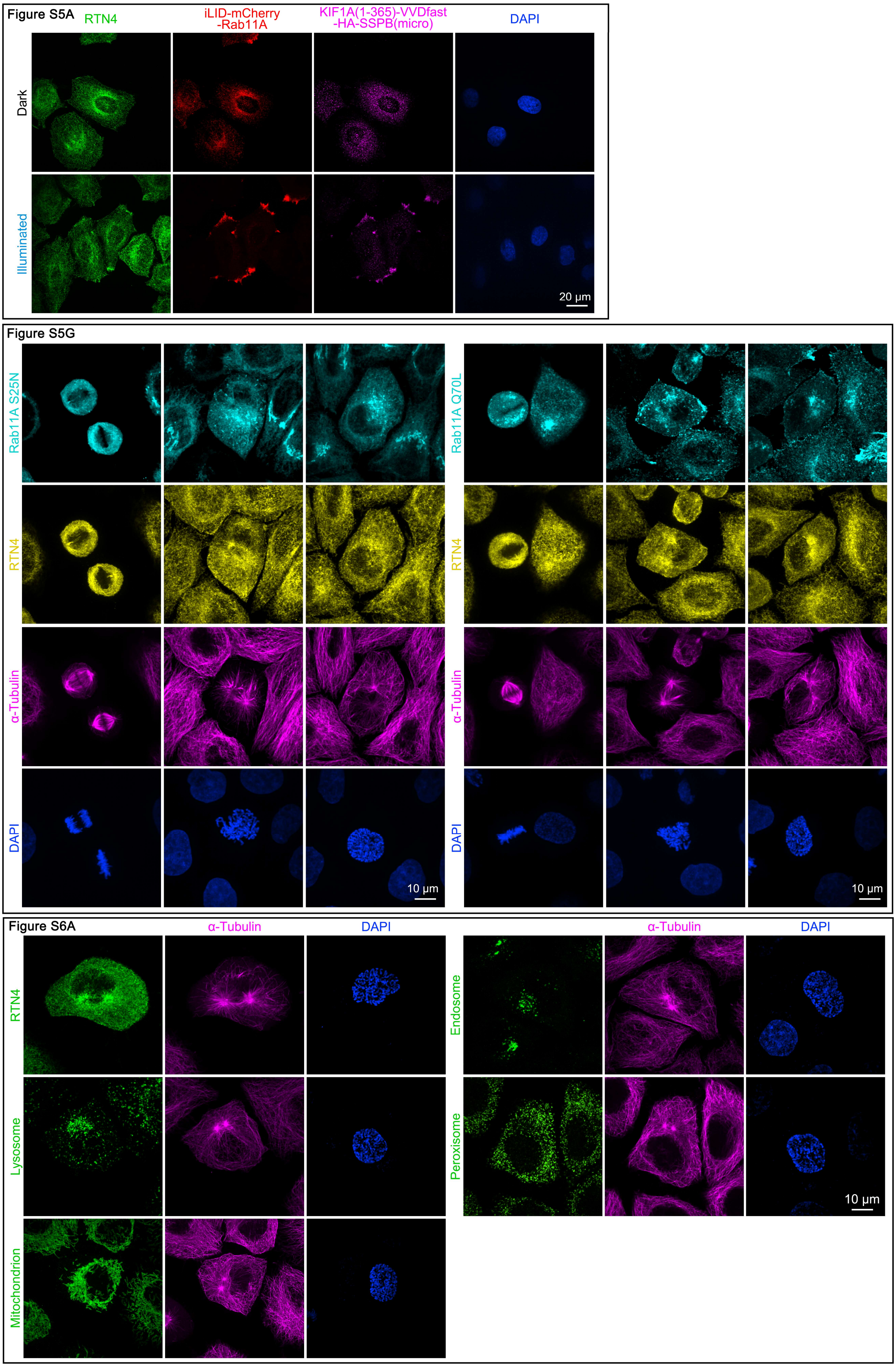

Raw data 15. Raw data of microscope images. Related to Figures S5–S6.

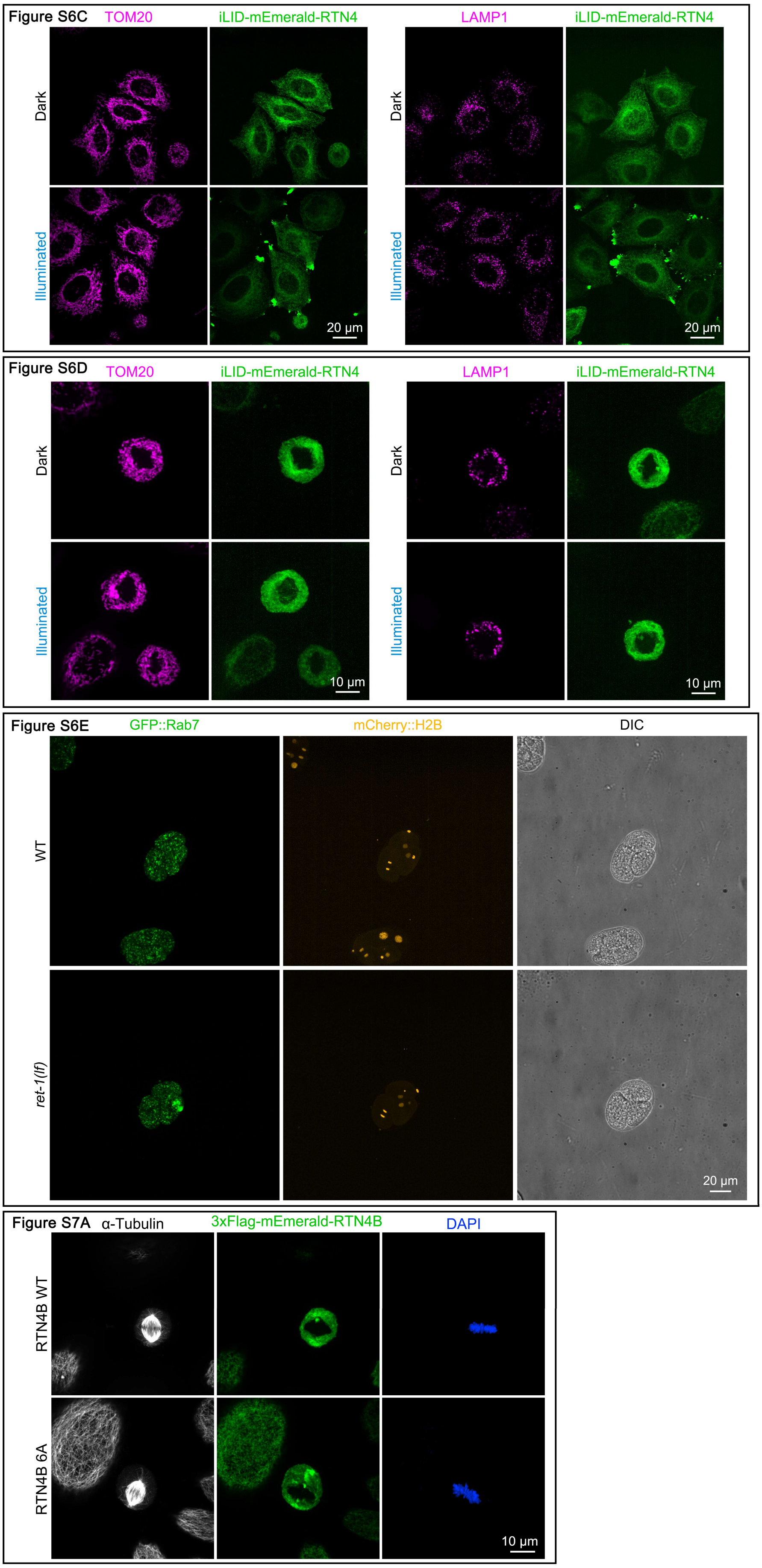

Raw data 16. Raw data of microscope images. Related to Figures S6–S7.

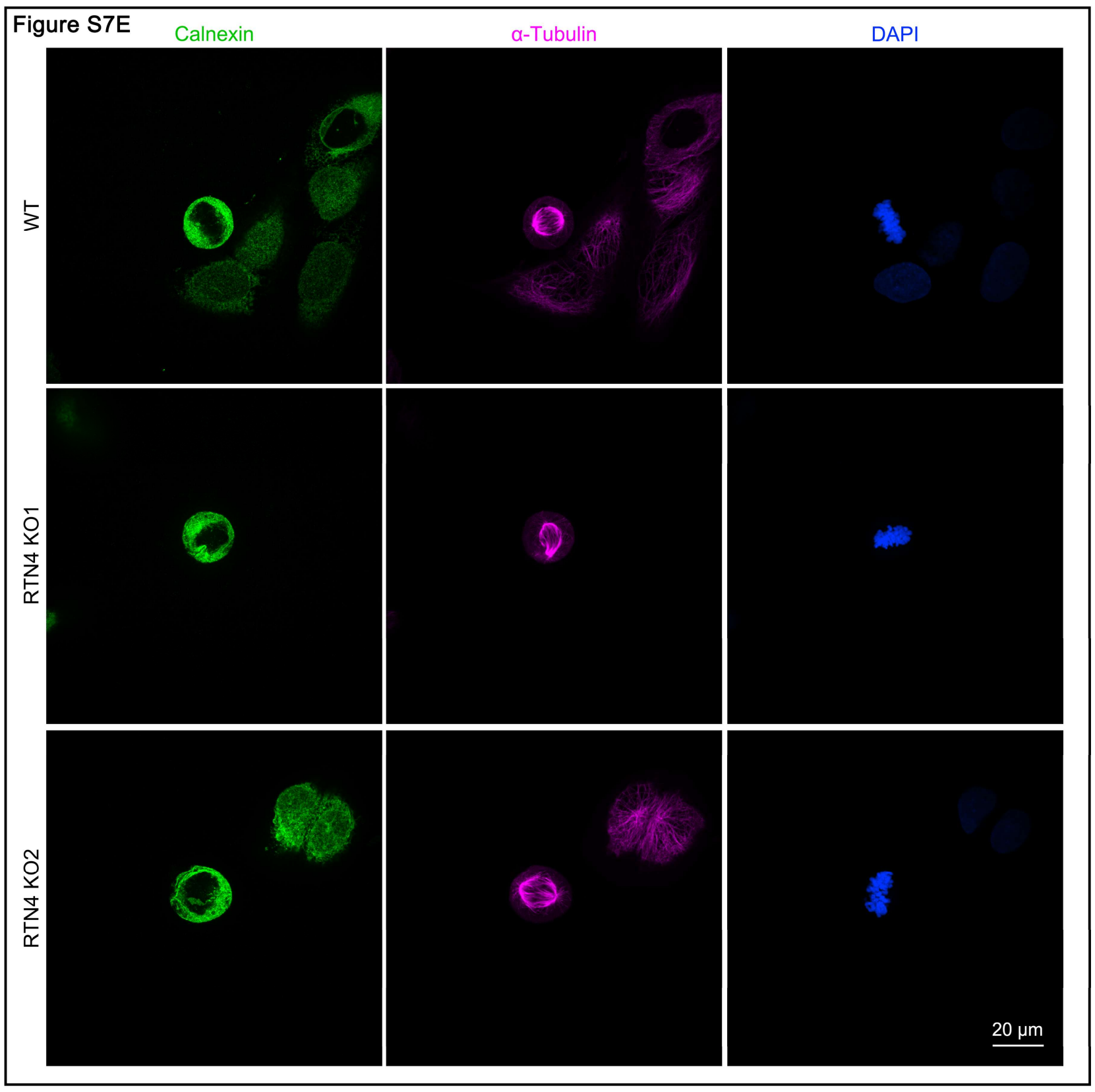

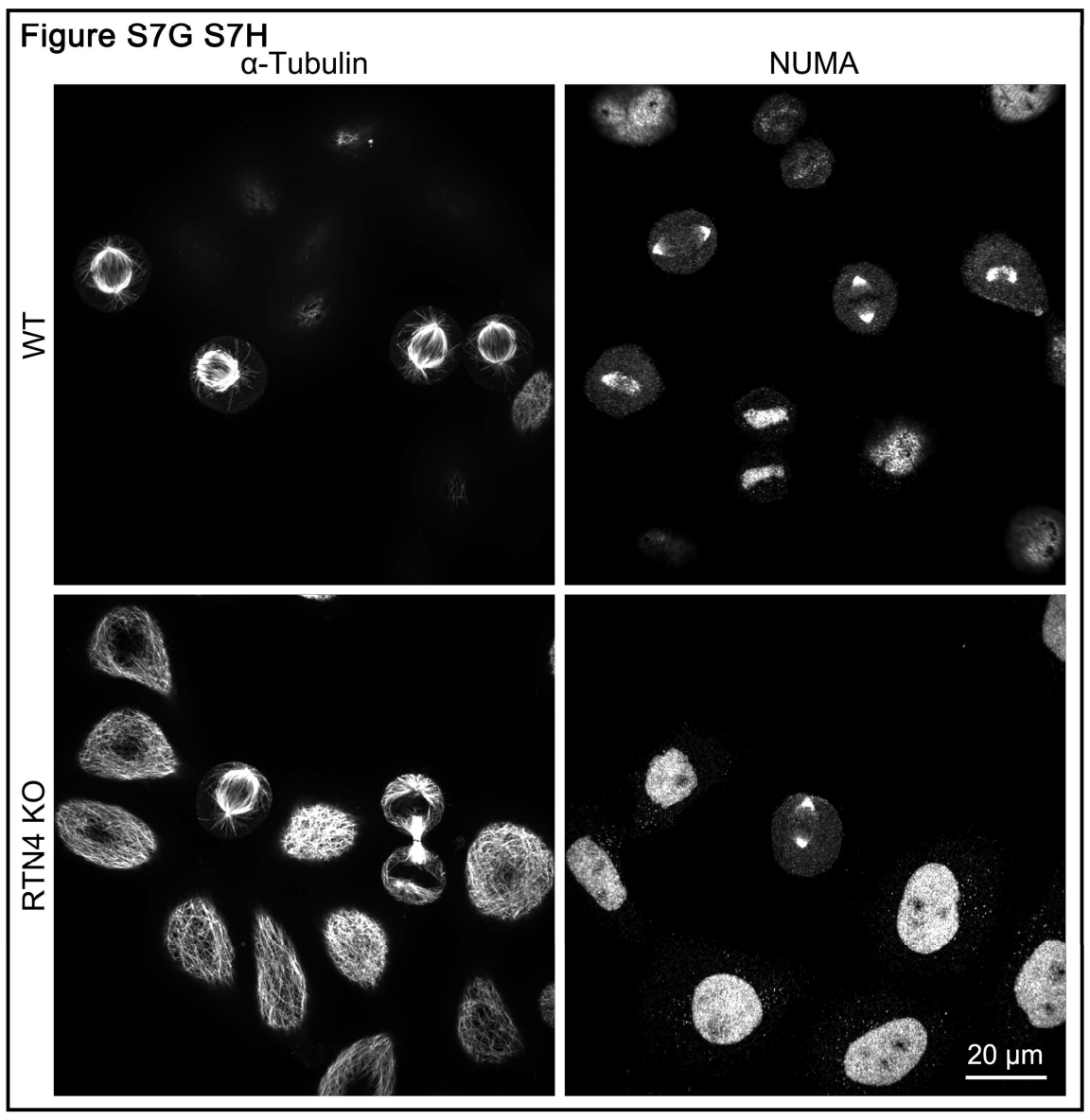

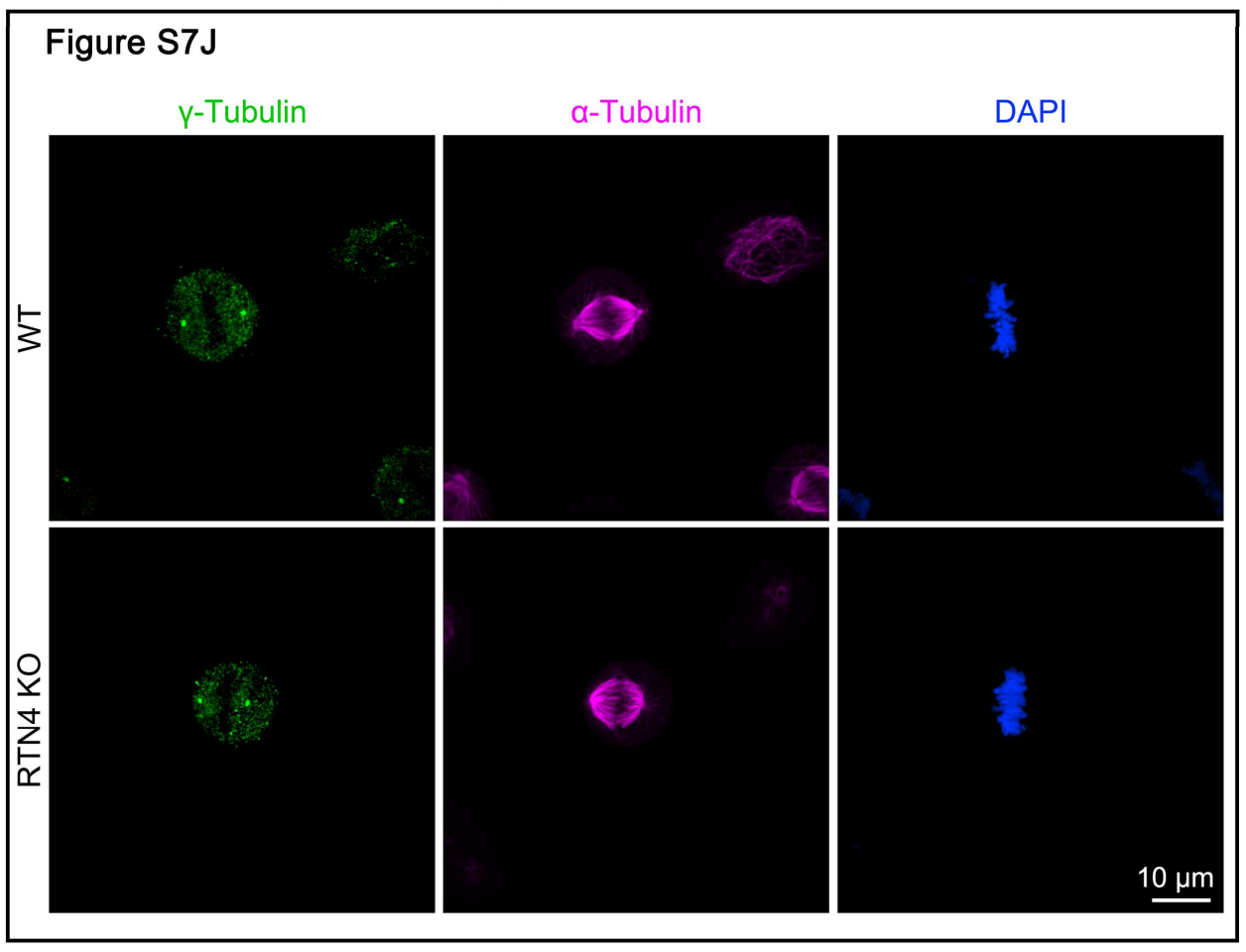

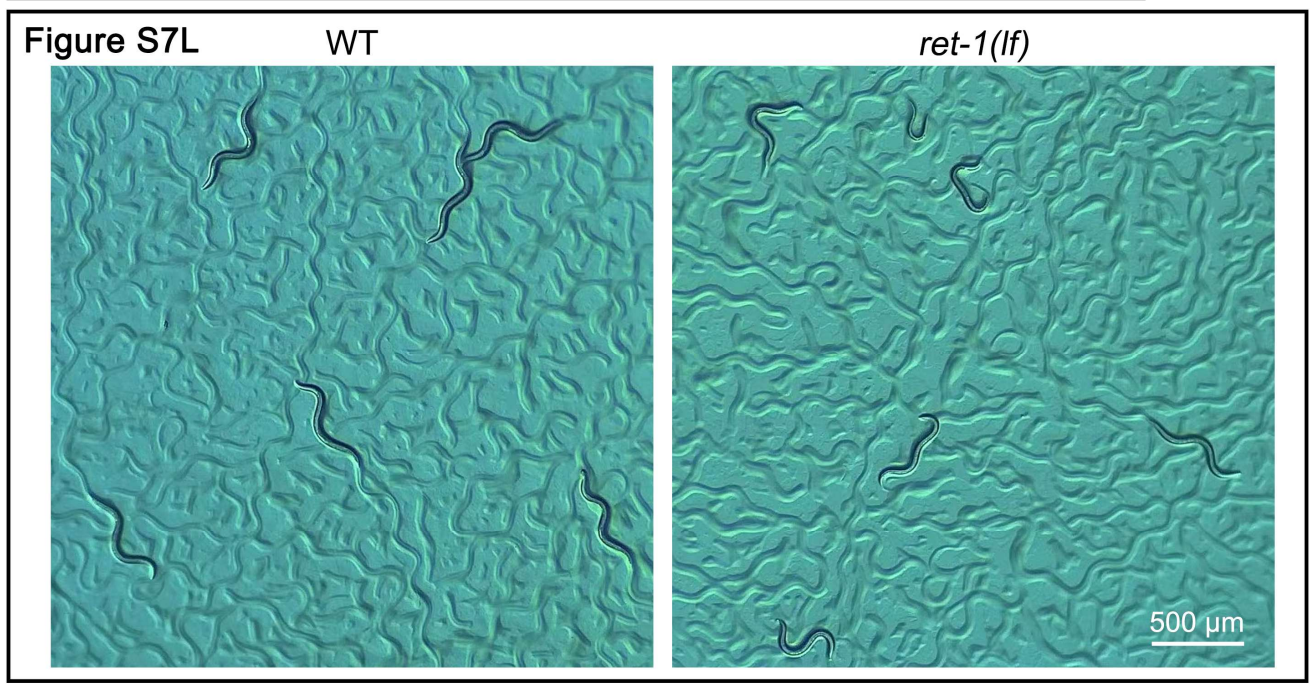

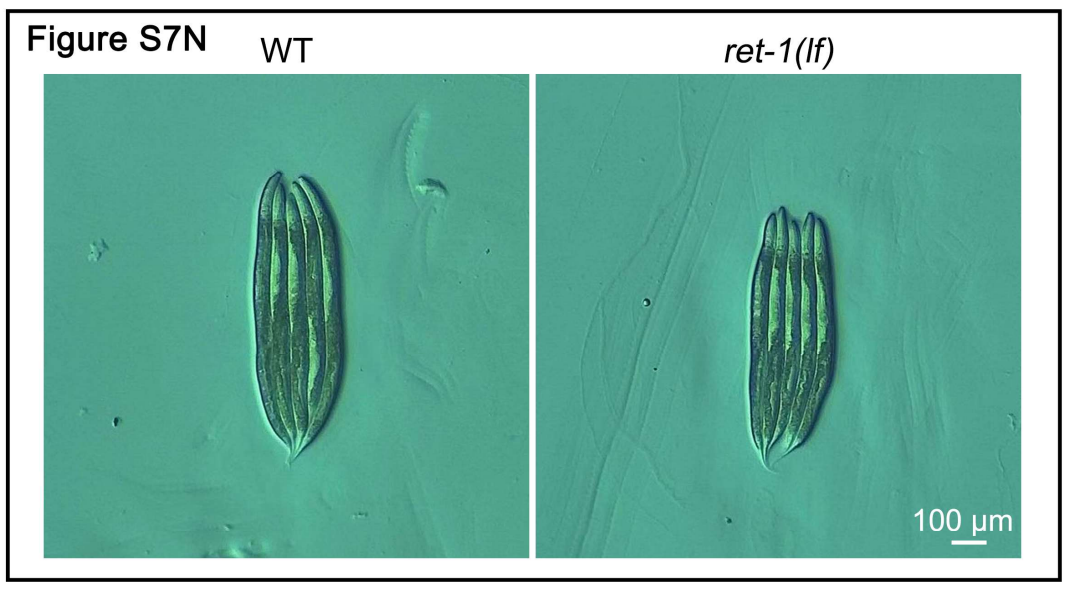

Raw data 17. Raw data of microscope images. Related to Figure S7.
