# Supplementary material for: Pericentrosomal Redistribution of the Endoplasmic Reticulum Ensures Organelle Symmetric Inheritance and Mitotic Progression
Source: Adv Sci (Weinh). 2026 Jun 22;13(51):e76193. doi: 10.1002/advs.76193 (PMC13337110; doi:10.1002/advs.76193)
Supplement: Supplementary file 2 [file ADVS-13-e76193-s002.docx]

Supporting Information

**Pericentrosomal Redistribution of the Endoplasmic Reticulum Ensures Organelle Symmetric Inheritance and Mitotic Progression**

Xiangyu Xu, Yalin Liu, Rongyi Wang, Wenwen Xu, Hao Shi, Ning Huang, Junlin Teng*, Jin Meng*, Pengli Zheng*, Jianguo Chen*

**Figure S1. ER-shaping proteins redistribute during mitosis. Related to Figure 1.**

(A) Representative images of RTN4 (green, tubular ER protein) and CLIMP63 (magenta, ER sheet protein) in interphase HeLa cells labeled with the indicated antibodies and DAPI (blue, DNA). Perinuclear (1) and peripheral regions (2) (outlined) are enlarged on the right. Scale bar, 10 μm.

(B) Representative images of ER protein distributions in metaphase HeLa cells labeled with an anti-α-tubulin (magenta) antibody and DAPI (blue). For tubular ER proteins (RTN4, RTN3, and REEP5), ER sheet proteins (CLIMP63, KTN1, and P180), and ER marker protein Calnexin, cells were immunolabeled with the appropriate antibodies. For pan-ER labeling, HeLa cells stably expressing the ER marker oxStayGold-KDEL were used. Maximal-intensity projections of *z*-stacks are shown. Pericentrosomal regions (spindle poles; outlined) are enlarged on the right. Scale bar, 10 μm.

(C, D) Representative images of the indicated ER protein (green) distributions in prophase, prometaphase, and metaphase COS7 (C) or RPE1 (D) cells labeled with an anti-α-tubulin (magenta) antibody and DAPI (blue). Scale bars, 10 μm.

(E, F) Time-lapse images of HeLa cells stably expressing 3×Flag-mEmerald-RTN4B (E, green) or the ER marker oxStayGold-KDEL (F, green). The cells also stably expressed H2B-mScarlet (cyan) and were stained with SiR-Tubulin (magenta). The time of nuclear envelope breakdown was set to zero; relative time (mm: ss) is shown. See also Video S1. Scale bars, 10 μm.

**Figure S2. RTN4 relocalization drives the tubularization of the pericentrosomal ER in early mitosis. Related to Figure 2.**

(A) The prometaphase HeLa cell selected for FIB-SEM imaging in Figure 2A. The outlined region was subjected to FIB-SEM. Scale bar, 5 μm.

(B) Serial sections of electron microscopy (EM) images of interphase HeLa cells. Arrowheads: centrosomes; arrows: nuclear envelope. ER elements are shown in red. Scale bar, 1 μm.

(C) Western blot of lysates from the indicated wild-type (WT) or knockout (KO) cells. GAPDH served as the loading control.

(D) Representative images of ER morphology in interphase HeLa cells: WT, single knockouts (RTN4 KO, RTN3 KO, and REEP5 KO), and double knockouts (RTN4/RTN3 DKO and RTN4/REEP5 DKO). Cells were immunolabeled for Calnexin (green). Scale bar, 10 μm.

(E) Prometaphase HeLa cells analyzed by EM in Figures 2H (1#) and S2F (2#). Scale bar, 10 μm.

(F) Representative EM images of WT and RTN4 KO HeLa cells in prometaphase, showing centrosomes (magenta) and pericentrosomal ER (white). Scale bar, 1 μm. Additional correlated examples are shown in Figure 2H.

(G) Quantification of the lengths of pericentrosomal ER elements in Figures 2H and S2F, with at least 128 ER elements counted per condition. Mann–Whitney test; *P* values are shown.

(H) Representative images of the distribution of the ER marker mCherry::SP12 (white) in interphase embryos from wild-type (WT) and *ret-1* loss-of-function (lf) mutant worms. *ret-1(lf)* indicates *ret-1(gk242)*. Scale bar, 10 μm.

(I) Representative images of mCherry::SP12 (white) distribution in mitotic embryos from WT and *ret-1(lf)* worms. Outlined regions are enlarged on the right. White arrows indicate pericentrosomal regions. Scale bar, 10 μm.

(J, K) Quantification of pericentrosomal fluorescence intensity (J) and pericentrosomal/cortical fluorescence-intensity ratio (K) of mCherry::SP12 in mitotic embryos (the AB cell) from WT and *ret-1(lf)* worms. 30 embryos per condition from three experiments were analyzed in (J) and (K). Data are mean ± s.d. Two-tailed unpaired Student’s *t*-test; *P* values are shown.

**Figure S3. RTN4 is phosphorylated during mitosis. Related to Figure 3.**

(A) Western blot of lysates from the indicated asynchronous cells and nocodazole (Noco)- or STLC-arrested prometaphase cells. Cyclin B1 is a marker for M phase. GAPDH served as the loading control.

(B) The three major isoforms of mammalian RTN4 share an identical C-terminal RHD region, of which RTN4A is the longest, containing a unique region of 800 amino acids.

(C) Western blot of RTN4 in wild-type (WT) and RTN4 knockout (KO) HeLa cells detected with two anti-RTN4 antibodies. Long exposure (l.e.) images of RTN4A (region 1) and RTN4C (region 2) are shown below. One anti-RTN4 antibody (Novusbio, NB100-56681; left) targets an N-terminal cytoplasmic epitope, whereas the other (Proteintech, 10740-1-AP; right) recognizes the C-terminal RHD region.

(D) HeLa cells transfected with 3×Flag-RHD were synchronized to prometaphase and subjected to western blotting.

(E) The 20 serine residues in the 1–185-aa N-terminal cytoplasmic region of RTN4B are shown in green.

(F) RTN4 phosphorylation sites in MS analysis. For the endogenous assay, lysates from asynchronous and nocodazole-arrested mitotic HeLa cells were immunoprecipitated with an anti-RTN4 antibody. For the exogenous assay, lysates from asynchronous and STLC-arrested mitotic HeLa cells stably expressing 3×Flag-mEmerald-RTN4B were immunoprecipitated with anti-Flag M2 affinity gels. The enriched endogenous and exogenous RTN4 protein samples were then analyzed by MS. Phosphorylation sites identified by MS are all located in the N-terminal region of RTN4B and are shown as red dots.

(G) Nocodazole-arrested mitotic HeLa cells were treated with CDK1 inhibitors (RO3306 or BMS-265246) and analyzed by western blot with the indicated antibodies.

(H) Coomassie Brilliant Blue staining of purified 3×Flag-mEmerald-RTN4B WT and 6A mutant from HeLa cells.

(I, J) Representative images (I) and quantification (J) of pericentrosomal RTN4 distribution in prometaphase HeLa cells treated with DMSO or BMS-265246. Cells were immunolabeled for RTN4 (green) and α-tubulin (magenta) in (I). DNA was stained with DAPI (blue). Maximal-intensity projections of z-stacks are shown. Pericentrosomal regions (outlined) are enlarged on the right. Dashed circles outline the positions of the centrosomes. Scale bar, 10 μm. *n* = 3 independent experiments, with at least 31 cells analyzed per condition for (J).

(K, L) Western blot (K) and representative images (L) of WT or RTN4 KO HeLa cells stably expressing 3×Flag-mEmerald-RTN4B WT, 6A or 6D (green). GAPDH served as the loading control. Cells were immunolabeled for γ-tubulin (magenta) in (L). DNA was stained with DAPI (blue). Pericentrosomal regions (outlined) are enlarged. Scale bar, 10 μm.

(M) Representative images of ER distribution and spindle positioning in RTN4 KO metaphase HeLa cells stably expressing 3×Flag-mEmerald-RTN4B WT, 6A or 6D (cyan). Cells were stained with DAPI (blue) and immunolabeled for Calnexin (yellow) and α-tubulin (magenta). Scale bar, 10 μm.

**Figure S4. RTN4 mitotic relocalization requires cytoplasmic dynein. Related to Figure 4.**

(A) Microtubule co-sedimentation assays of asynchronous and nocodazole-arrested mitotic HeLa cells. Pellet (P) after incubation at 37 °C: microtubule-bound fraction; Supernatant (S): unbound fraction. The 4 °C incubation served as a microtubule-free control.

(B, C) Representative images (B) and quantification (C) of pericentrosomal RTN4 (green) intensity in HeLa cells treated with DMSO, STLC, or nocodazole. Scale bar, 10 μm. *n* = 3 independent experiments, with at least 33 cells analyzed per condition in (C).

(D) Representative images of RTN4 (green) distribution in prometaphase and metaphase HeLa cells treated with DMSO or the dynein inhibitor dynarrestin. Scale bars, 10 μm.

(E) Western blot analysis of cells transfected with negative control (NC) or p150^Glued^ siRNA. GAPDH served as the loading control.

(F, G) Representative images (F) and quantification (G) of pericentrosomal RTN4 (green) distribution in prometaphase HeLa cells transfected with NC or p150^Glued^ siRNA. Pericentrosomal regions (outlined) are enlarged on the right. Dashed circles outline the positions of the centrosomes. Scale bar, 10 μm. *n* = 3 independent experiments, with at least 42 cells analyzed per condition in (G).

(H) Lysates from mitotic HeLa cells stably expressing 3×Flag-mEmerald-RTN4B were immunoprecipitated with anti-Flag M2 affinity gels, and the interaction was evaluated by western blot.

(I) Representative images of mitotic HeLa cells stably expressing Rab proteins (cyan), including mEmerald-Rab1A, mEmerald-Rab1B, mEmerald-Rab5A, mEmerald-Rab7A, mEmerald-Rab11B, and mEmerald-Rab25. Cells were stained with DAPI (blue) and immunolabeled for RTN4 (yellow) and α-tubulin (magenta). Scale bars, 10 μm.

Data in (C) and (G) are presented as mean ± s.e.m. across replicates. Statistical tests were two-tailed unpaired Student’s *t*-test. *P* values are shown.

**Figure S5. Rab11 mediates pericentrosomal redistribution of RTN4 during early mitosis. Related to Figure 4.**

(A) Representative images of interphase HeLa cells expressing the indicated optogenetic system constructs in the dark or after 10 min of illumination with blue light. Cells were immunolabeled with anti-RTN4 (green) and anti-HA (magenta) antibodies. The peripheral regions of the cells (outlined) are enlarged on the right. Scale bar, 10 μm.

(B) Lysates from HEK293T cells co-transfected with mEmerald-RTN4B and the vector, 3×Flag-Rab11A, 3×Flag-Rab11B, or 3×Flag-Rab25 were immunoprecipitated with anti-Flag M2 affinity gels. The samples were analyzed by western blotting with the indicated antibodies.

(C) Coomassie Brilliant Blue staining of purified GST-Rab11A from Rosetta (DE3) cells.

(D) STLC-arrested mitotic HeLa cells treated with DMSO or the CDK1 inhibitor RO3306 were subjected to immunoprecipitation with anti-GFP nanobody agarose beads, and the interaction was evaluated by western blot.

(E) Lysates from STLC-arrested mitotic HeLa cells expressing 3×Flag-FIP3 and stably expressing mEmerald-Rab11A and 2×Strep-HA-RTN4B were immunoprecipitated with Strep-Tactin XT resins, and the interaction was evaluated by western blot.

(F) Lysates from STLC-arrested mitotic HeLa cells stably expressing mEmerald-Rab11A WT, S25N, or Q70L were immunoprecipitated with anti-GFP nanobody agarose beads, and the interaction was evaluated by western blot.

(G, H) Representative images (G) and quantification (H) of the distribution of RTN4 and Rab11A mutants during mitosis. HeLa cells stably expressing the indicated mEmerald-Rab11A constructs (cyan) were stained with DAPI (blue) and immunolabeled for RTN4 (yellow) and α-tubulin (magenta). Scale bars, 10 μm. *n* = 3 independent experiments, with at least 35 cells analyzed per condition in (H). Data are presented as mean ± s.e.m. across replicates. Statistical tests were one-way ANOVA. *P* values are shown.

**Figure S6. RTN4 redistribution contributes to symmetric organelle partitioning. Related to Figure 5.**

(A) Representative images of organelle (green) distributions in prophase HeLa cells. Markers used: anti-Lamp1 for lysosomes, anti-TOM20 for mitochondria, anti-EEA1 for endosomes, and anti-PEX14 for peroxisomes. Pericentrosomal regions (outlined) are enlarged on the right. Scale bar, 10 μm.

(B) Schematic representation of the optogenetic system for transporting and repositioning RTN4. Upon blue-light (470 nm) illumination, iLID-mEmerald-RTN4 binds to the active KIF1A motor, activating microtubule plus-end-directed transport of RTN4.

(C, D) Representative images of interphase (C) and metaphase (D) HeLa cells expressing the indicated optogenetic system constructs in the dark or after 10 min of illumination with blue light. Cells were immunolabeled for TOM20 (mitochondria, magenta; top) or LAMP1 (lysosomes, magenta; bottom). Maximal-intensity projections of *z*-stacks are shown. The white dashed line shows the equatorial plate in (D). Scale bars, 10 μm.

(E, F) Representative images (E) and quantification (F) of the partitioning of the lysosome marker GFP::RAB-7 (green) in anaphase embryonic cells from wild-type (WT) and *ret-1* loss-of-function (lf) mutant worms. *ret-1(lf)* indicates *ret-1(gk242)*. White dashed circles in the first column outline the daughter cells, and those in the second column outline the chromosome regions (mCherry::H2B, yellow) in (E). Scale bar, 10 μm. At least 24 embryos per condition from three experiments were analyzed in (F). Data are mean ± s.d. Two-tailed unpaired Student’s *t*-test; *P* values are shown.

**Figure S7. RTN4 regulates metaphase spindle dynamics. Related to Figure 6.**

(A) Representative images of astral microtubules contacting the cell cortex in HeLa cells stably expressing 3×Flag-mEmerald-RTN4B WT or 6A (green). Cells were immunolabeled for α-tubulin (white). DNA was stained with DAPI (blue). White arrows indicate astral microtubules contacting the cell cortex. Scale bar, 10 μm.

(B) Quantification of the ratio of astral microtubule contacts with the cell cortex on the two sides of the spindle pole. *n* = 3 independent experiments, with at least 57 cells analyzed per condition.

(C) Schematic illustration of the method for quantifying metaphase spindle positioning in the *x-y* plane. The ratio of the distances from the two spindle poles to the cell cortex is defined as the distance ratio. The smaller the distance ratio, the greater the degree of spindle deviation.

(D) Western blot of wild-type (WT) and RTN4 knockout (KO) U2OS cells. GAPDH served as the loading control.

(E, F) Representative images (E) and quantification (F) of spindle positioning in the *x-y* plane in WT and RTN4 KO metaphase U2OS cells. Cells were immunolabeled for Calnexin (green) and α-tubulin (magenta). Scale bar, 10 μm. *n* = 3 independent experiments, with at least 48 cells analyzed per condition in (F).

(G, H) Representative images of astral microtubule (G) and cortical NUMA (H) distributions in WT and RTN4 KO metaphase HeLa cells. Cells were immunolabeled for α-tubulin (G) or NUMA (H). White arrows indicate lateral contacts between astral microtubules and the cell cortex in (G) or NUMA accumulation on the cell cortex in (H). Scale bars, 10 μm.

(I) Schematic illustration of the spindle orientation measurement along the *z*-axis. The spindle tilt angle α was calculated as arctan (*a/b*), where *a* and *b* represent the vertical and horizontal distances between the two spindle poles, respectively, in the 3D projection.

(J) Representative images of the *z*-axis orientation of the spindle in WT and RTN4 KO metaphase HeLa cells. The spindle orientation in the *x-z* plane is shown at the bottom. Cells were stained with DAPI (blue) and immunolabeled for γ-tubulin (green) and α-tubulin (magenta). Scale bar, 10 μm.

(K) Quantification of the *z*-axis orientation of the spindle in WT and RTN4 KO metaphase HeLa cells. *n* = 3 independent experiments, with at least 30 cells analyzed per condition.

(L) Representative images of WT and *ret-1* loss-of-function (lf) mutant worms at 49 h after egg-laying. *ret-1(lf)* indicates *ret-1(gk242)*. Scale bar, 500 μm.

(M) Proportion of worms at the four larval stages (L1–L4) in (L). *n* = 4 experiments, with at least 646 animals counted per condition.

(N, O) Representative images (N) and quantification (O) of body length of WT and *ret-1(lf)* worms at 65 h after egg-laying. Scale bar, 100 μm. 60 worms per condition from three experiments were analyzed in (O). Data are mean ± s.d.

Data in (B), (F), (K) and (M) are presented as mean ± s.e.m. across replicates. Statistical tests were one-way ANOVA (F), two-tailed unpaired Student’s *t*-test (B, K, O), and Chi-square test (M). *P* values are shown.

**Video S1**. RTN4B and pan-ER dynamics during mitosis, related to Figure S1E and S1F. Maximum-intensity projection time-lapse of HeLa cells stably expressing 3×Flag-mEmerald-RTN4B (green, A; 2 min per frame) or the ER marker oxStayGold-KDEL (green, B; 2 min 12 s per frame). The cells also stably expressed H2B-mScarlet (cyan) and were stained with SiR-Tubulin (magenta). Relative time (h: mm: ss) is shown. Scale bar, 10 μm.

**Video S2**. 3D reconstruction of pericentrosomal and peripheral ER from FIB-SEM data in a prometaphase HeLa cell, related to Figure 2A. Pericentrosomal ER (red) and peripheral ER (blue) were 3D-rendered from FIB-SEM images using Amira. Centrosomes are marked in yellow. Scale bar, 1 μm.

**Video S3.** Mitotic duration of wild-type (WT), RTN4 knockout (KO), and RTN4/RTN3 double-knockout (DKO) HeLa cells, related to Figure 6F. Maximum-intensity projection time-lapse of the indicated HeLa cells stably expressing H2B-mScarlet (white) at 1 min 30 s per frame. WT, (A); KO, (B); DKO, (C). The time of nuclear envelope breakdown was set to zero; relative time (h: mm: ss) is shown. Scale bar, 10 μm.
